# Cytotoxic CD4^+^ T cells: origin, biological functions, diseases and therapeutic targets

**DOI:** 10.1038/s41392-025-02533-z

**Published:** 2026-03-09

**Authors:** Longyong Lai, Shuan Ran, Yuan Li, Jikai Cui, Xi Zhang, Jizhang Yu, Yanqiang Zou, Cheng Zhou, Jiahong Xia, Jie Wu

**Affiliations:** 1 Department of Cardiovascular Surgery, Union Hospital, Tongji Medical College, Huazhong University of Science and Technology, Wuhan, China; 2 Center for Translational Medicine, Union Hospital, Tongji Medical College, Huazhong University of Science and Technology, Wuhan, China; 3 Institute of Translational Medicine, Tongji Medical College, Huazhong University of Science and Technology, Wuhan, China; 4 Hubei Key Laboratory of Biological Targeted Therapy, Union Hospital, Tongji Medical College, Huazhong University of Science and Technology, Wuhan, China; 5 Key Laboratory of Organ Transplantation, Ministry of Education; NHC Key Laboratory of Organ Transplantation; Key Laboratory of Organ Transplantation, Chinese Academy of Medical Sciences, Wuhan, China

**Keywords:** Lymphocytes, Adaptive immunity

## Abstract

Cytotoxic CD4^+^ T lymphocytes are a unique subset of CD4^+^ T cells characterized by their cytokine secretion and cytolytic activity. Unlike traditional CD4^+^ helper T cells, cytotoxic CD4^+^ T lymphocytes exhibit similar cytotoxicity to that of CD8^+^ T cells, revealing unexpected plasticity in CD4^+^ T-cell functions. This flexibility suggests that CD4^+^ T cells can transform and acquire effector functions beyond their conventional functions, emphasizing their importance in various immune responses. Despite the identification of cytotoxic CD4^+^ T lymphocytes decades ago, recent advancements have broadened our understanding of their phenotypic diversity, transcriptional regulation, differentiation pathways, and functional roles. Cytotoxic CD4^+^ T lymphocytes play pivotal roles across a broad spectrum of diseases, including cancer, infectious diseases, autoimmune disorders, and cardiovascular diseases. They typically mediate strong inflammatory responses and kill target cells through cytotoxicity, playing a crucial role in maintaining immune homeostasis. In this review, we synthesize current findings on cytotoxic CD4^+^ T lymphocytes, emphasizing their origin, biomarkers, regulatory molecules, and biological functions. Additionally, we focus on their pathological roles in the progression of various diseases and examine how cytotoxic CD4^+^ T lymphocytes contribute to disease development and progression. We also provide a comprehensive summary of therapeutic strategies targeting cytotoxic CD4^+^ T cells and review the associated clinical trial data, aiming to propose new strategies for disease management through the targeting of cytotoxic CD4^+^ T lymphocytes.

## Introduction

T cells are essential components of the adaptive immune system, mediating defense against pathogens and tumors while contributing to immune regulation. They are broadly classified into CD4⁺ and CD8⁺ subsets according to the type of major histocompatibility complex (MHC) molecules they recognize. CD8⁺ T cells typically engage MHC class I molecules and directly eliminate infected or malignant cells, whereas CD4⁺ T cells recognize MHC class II molecules and function primarily through cytokine secretion and immune coordination; hence, they are termed helper T (Th) cells.^1,2^ CD4^+^ and CD8^+^ T cells interact closely with other immune cells, including dendritic cells (DCs), macrophages, and B cells, to coordinate a robust immune response. This cooperation facilitates the formation of immunological memory and helps maintain immune homeostasis within the body.^3^

However, a subset of CD4⁺ T cells with cytotoxic potential, known as cytotoxic CD4⁺ T lymphocytes (CD4⁺ CTLs), possesses the ability to kill MHC class II–expressing target cells directly, thereby blurring the traditional dichotomy between helper and cytotoxic T-cell functions. Notably, CD4⁺ CTLs can compensate for exhausted CD8⁺ T cells during chronic infections and exhibit robust proinflammatory cytokine production reminiscent of Th1 responses.^4,5^ Owing to these characteristics, CD4^+^ CTLs are now recognized as a double-edged sword in immune regulation. On the one hand, they play a crucial role in eliminating infected or malignant cells, particularly in chronic infections or tumors where CD8^+^ T cells might fail to act effectively because of exhaustion or suppression. On the other hand, their cytotoxic activity can lead to tissue damage in autoimmune diseases, such as systemic lupus erythematosus (SLE) or rheumatoid arthritis (RA), where they mistakenly target host tissues.^6,7^ Additionally, recent studies have confirmed the pathological roles of CD4^+^ CTLs in a variety of diseases, including cancer,^8,9^ infectious diseases,^10,11^ cardiovascular diseases,^12–14^ and metabolic disorders.^15,16^ These findings further emphasize the importance of CD4^+^ CTLs in both immune surveillance and pathological conditions, highlighting their potential as therapeutic targets for a broad range of diseases.

Here, we aim to summarize key information regarding the history, origin, biological functions, and other relevant aspects of CD4^+^ CTLs. We specifically focus on reviewing and analyzing the latest research findings concerning the roles, mechanisms, and potential therapeutic value of these cells in various diseases. Additionally, we compile targeted therapeutic strategies aimed at CD4^+^ CTLs and associated clinical research progress, with the goal of providing an integrated perspective that may inform future research and clinical applications.

## Research history and milestone events of CD4^+^ CTLs

Approximately four decades ago, C. Feighery and P. Stastny first reported that a subset of cytotoxic lymphocytes could recognize cells expressing HLA-D region molecules, resulting in target cell lysis and subsequent death. This was the earliest documented study on CD4^+^ CTLs.^17^ In 1984, these cytotoxic CD4^+^ T cells were successfully identified in in vitro cultures following a mixed lymphocyte reaction.^18^ However, owing to the extended culture conditions, these findings were initially dismissed as experimental artifacts rather than authentic physiological phenomena. Subsequent studies using murine influenza models provided compelling evidence supporting the existence of this MHC class II-restricted cytotoxic T-cell subset.^19,20^ These studies further demonstrated that CD4^+^ CTLs mediate target cell destruction through mechanisms that are fundamentally similar to those employed by CD8^+^ CTLs. Since then, research efforts have been dedicated to elucidating the origin, functional properties, and physiological relevance of CD4^+^ CTLs, further illuminating their roles in immune regulation and disease pathogenesis.

In vitro studies by Chang et al. were the first to demonstrate that Th1 cells can mediate the direct cytotoxic killing of antigen-presenting cells (APCs), suggesting that this mechanism may function as a negative feedback loop in immune responses, limiting excessive inflammation.^21^ This discovery was groundbreaking because it challenged the traditional view that Th1 cells primarily function as helper cells, directing other immune cells, such as cytotoxic CD8^+^ T cells, rather than engaging in direct cytotoxicity themselves. Later, Lancki et al. reported that certain Th2 cell subsets can also express granzymes and perforin, which allows them to lyse nucleated target cells.^22^ Additional research revealed the presence of cytotoxic subpopulations in other CD4^+^ T-cell subsets, including Th0,^23^ T follicular helper (Tfh),^24^ and even regulatory T (Treg) cells.^25^ These findings imply that these cells could represent a distinct differentiation state of CD4^+^ T cells rather than being confined to a specific subset and highlight the plasticity and functional versatility of CD4^+^ T cells. Notably, these cells exhibit unique surface marker characteristics. Studies have indicated that this population typically lacks expression of the costimulatory molecule CD28 but preferentially expresses killer activation receptors (KARs). This distinctive phenotypic feature suggests potential functional or developmental connections with natural killer (NK) cells,^26^ providing valuable insights into the interplay between adaptive and innate immunity and urging a reassessment of the coordination mechanisms underlying immune responses. These findings explore the origin and physiological functions of CD4^+^ CTLs while also shifting the focus of research toward how the plasticity of CD4^+^ T cells contributes to their diverse functions and their potential roles in various diseases.

As previously mentioned, CD4^+^ CTLs were initially validated in a murine influenza model. Subsequent studies revealed that these cells expand in various other infectious diseases, particularly chronic viral infections, where they play a critical role in eliminating infected cells.^27–29^ Their ability to effectively target infected cells provides an important backup mechanism in these contexts, particularly when viral persistence challenges immune control. In 1996, Schmidt et al. were the first to identify the clonal expansion of CD4^+^ CTLs in patients with RA, demonstrating their infiltration into synovial tissues and their long-term persistence, thus revealing their key role in the pathogenesis of RA.^30^ These findings suggest that CD4^+^ CTLs play crucial roles not only in responding to infections but also in driving pathological autoimmune responses, which are traditionally thought to be mediated by CD8^+^ CTLs. Weyand et al. subsequently investigated the relationship between CD4^+^ CTLs and aging and reported that these cells have an extended lifespan, secrete high levels of IFN-γ and IL-2, and contribute to an immune environment skewed toward autoreactivity.^31^ This observation provides a new perspective on how age-related immune dysfunction can promote chronic inflammation. In the field of cardiovascular diseases, Liuzzo et al. were the first to report the presence of CD4^+^ CTLs in patients with cardiovascular conditions, suggesting that their clonal expansion is linked to persistent antigenic stimulation.^32^ The chronic activation of these cells may contribute to the pathogenesis of atherosclerosis and other cardiovascular conditions by promoting inflammation in blood vessels. Moreover, Xie et al. reported that naive tumor/self-specific CD4^+^ T cells can naturally differentiate into CD4^+^ CTLs in melanoma, leading to tumor regression and depigmentation.^33^ This discovery illustrates a potential mechanism by which the immune system can directly target and combat cancer through the activation of CD4^+^ CTLs, offering new insights into cancer immunotherapy. Understanding the molecular mechanisms underlying their differentiation and function in different pathological contexts remains a critical focus of ongoing research. Further exploration of these mechanisms will not only help clarify the unique roles of CD4^+^ CTLs across various diseases but also provide insights into their potential therapeutic applications.

In recent years, the rapid advancement of technologies such as flow cytometry and single-cell omics has revolutionized our ability to conduct in depth, high-resolution analyses of specific cell populations. These technologies have provided unprecedented insights into the heterogeneity of immune cells, offering a detailed understanding of their functional states and mechanisms of action. In terms of development, Patil et al. utilized single-cell differential gene expression analysis to demonstrate that the T_EMRA_ subset (effector memory T cells expressing CD45RA) exhibited an upregulation of genes linked to cytotoxicity and costimulatory functions.^34^ These findings not only suggest that these cells may serve as progenitors of CD4^+^ CTLs but also emphasize the plasticity of memory T-cell subsets, further highlighting the complexity and adaptability of the immune system in response to various challenges. Similarly, Oja et al. reported that the transcription factor Hobit is expressed at elevated levels in CD4^+^ CTLs, which not only highlights its potential as a specific biomarker for this subset but also opens new avenues for the targeted identification of these cells.^35^ Moreover, Devarajan et al. demonstrated that this population, which lacks costimulatory molecules, can develop independently of antigen presentation in response to signals triggered by ongoing infections, with support from IL-15.^36^ These findings significantly deepen our understanding of the intricate mechanisms that govern the differentiation, activation, and functional dynamics of CD4^+^ CTLs.

The functions of immune cells are intricately tied to their tissue localization, a factor that critically influences their immune responses. Historically, the precise identification of CD4^+^ CTL tissue localization has been hindered by technological constraints and the complexity of these cells. However, the rapid advancement of single-cell technologies has provided unprecedented clarity, enabling researchers to probe the niches of CD4^+^ CTLs more effectively. Tanemoto et al. discovered clusters of CD4^+^ CTLs in the human intestinal tract, more recently.^37^ This discovery not only deepened our understanding of mucosal immune responses but also opened new avenues for exploring tissue-specific immune functions. Furthermore, Liang et al. identified CD4^+^ CTLs in the tonsils, a major lymphoid organ, emphasizing their importance in both peripheral and central immune responses.^38^ Previous studies have suggested that CD4^+^ CTLs infiltrate tumor tissues in cancer patients, contributing to the formation of the tumor microenvironment (TME). However, the precise mechanisms underlying this process remain to be fully elucidated. Sultan et al. further expanded our understanding by revealing the dual role of CD4^+^ CTLs in antitumor immunity. These cells not only target and kill tumor cells but also exert an immunosuppressive effect by depleting DCs, thereby hindering the overall immune control of the tumor.^9^ This dual functionality highlights the complex role that CD4^+^ CTLs play in shaping the immune landscape of cancer. These studies highlight the dynamic role of CD4^+^ CTLs in maintaining immune homeostasis and demonstrate their multifunctionality across diverse tissue environments.

The clinical application of CD4^+^ CTLs has also made groundbreaking progress in recent years. Melenhorst et al. employed long-lasting CD19-targeting chimeric antigen receptor (CAR) T cells to treat two leukemia patients, in whom highly activated CD4^+^ T-cell populations were identified and became the predominant CAR T-cell population in the later stages of treatment. Single-cell profiling revealed that these long-lived CD4^+^ CAR-T cells not only exhibited cytotoxic characteristics but also displayed sustained functional activation and proliferation, suggesting that compared with CD8 + CAR-T cells, CD4 + CAR-T cells may represent a superior therapeutic strategy for long-term treatment.^39^

**Fig. 1:**
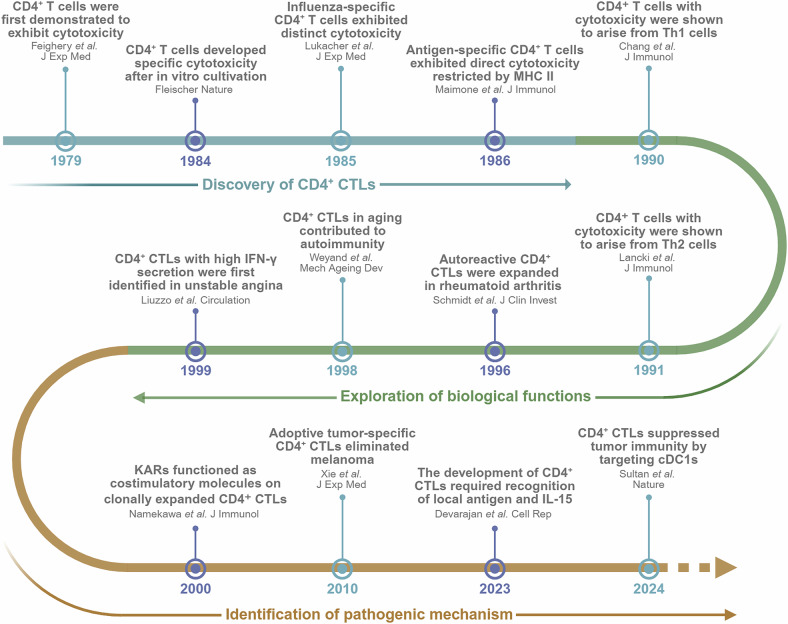
Research history and milestone events of CD4^+^ CTLs. This timeline highlights the significant discoveries and advancements in the study of CD4^+^ CTLs. The research history of CD4^+^ CTLs can be broadly divided into three phases: the discovery of CD4^+^ CTLs, the exploration of their biological functions, and the identification of their pathogenic mechanisms. The early recognition of cytotoxicity in CD4^+^ T cells began in 1979, with foundational studies establishing their potential to acquire cytotoxic functions. Research has focused on uncovering their origins, phenotypes, functions, and pathological mechanisms in various diseases. Ongoing studies continue to expand our understanding of the roles of CD4^+^ CTLs in immunity, disease progression, and potential therapeutic applications. MHC major histocompatibility complex, Th helper T, IFN-γ interferon-γ, KARs killer activation receptors, IL-15 interleukin-15, cDC1s type 1 conventional dendritic cells. Created at https://BioRender.com

In summary, these milestone discoveries in CD4^+^ CTL research have consistently deepened and broadened our understanding of this unique T-cell subset. Their involvement in immune responses—not only as cytotoxic agents but also in modulating the immune environment—offers a distinct perspective on immune cell therapy. Ongoing research into CD4^+^ CTLs promises to unlock new therapeutic strategies, offering hope for more effective and targeted treatments in the near future (Fig. 1).

## Origins of CD4^+^ CTLs

### Hypothesis on the origin of CD4^+^ CTLs

Current research presents two prominent hypotheses regarding the origin of CD4^+^ CTLs. One hypothesis is that CD4^+^ CTLs may originate from NK cell precursors, given that these cells express several receptors from the killer Ig-like receptor (KIR) family, which are typically found on NK cells, in addition to the TCR. Alternatively, CD4^+^ CTLs could directly arise from other CD4^+^ T cells, representing a terminally differentiated state. Despite these two competing theories, the exact origin of CD4^+^ CTLs remains uncertain and is likely influenced by various immune environments and pathological conditions.

Some researchers have proposed that CD4^+^ CTLs may originate from NK cell precursors. These cells are endowed with various receptors, such as killer immunoglobulin-like receptors (KIRs) and killer cell-activating receptors (KARs), both of which are members of the immunoglobulin superfamily. NK cells, as innate immune effectors, rely heavily on KIRs and KARs to distinguish between healthy and infected or transformed cells, providing a first line of defense. This unique feature enables CD4^+^ CTLs to recognize MHC class I molecules on target cells as well. CD4^+^ CTLs exhibit diverse and dynamic KIR gene expression patterns, which evolve sequentially following TCR rearrangement. The aberrant expression of KIRs in CD4^+^ CTLs is closely linked to their cytotoxic activity, providing a critical mechanism for their involvement in immune responses.^40^ For example, KIR2DS2 specifically interacts with MHC class I-presented human heat shock protein 60 (hHSP60), triggering cytotoxicity in CD4^+^ CTLs. Interestingly, TCR stimulation by hHSP60 alone does not induce a cytotoxic response, underscoring the specificity of CD4^+^ CTL activation, which is KIR dependent.^41^ This highlights the importance of coreceptor signaling in fine-tuning immune responses. Furthermore, KARs play an essential role in modulating CD4^+^ CTL functionality by enhancing their proliferative response to TCR-mediated activation without significantly altering their cytotoxic functions.^26^ Additionally, the aberrant expression of KARs in the absence of KIRs and MHC I molecules can result in the expansion of autologous reactive CD4^+^ CTL clones.^42^ The interplay between KARs, KIRs, and MHC I molecules in CD4^+^ CTLs forms a critical regulatory network. This network not only fine-tunes the cytotoxic potential of CD4^+^ CTLs but also connects the adaptive immune system with the innate immune system.

Key markers typically associated with NK cells, such as natural killer group 2 member A (NKG2A) and member D (NKG2D), are also expressed on the surface of CD4^+^ CTLs.^43–45^ The presence of these markers highlights the increasing recognition of the functional similarities between CD4^+^ CTLs and NK cells. NKG2A, a member of the C-type lectin receptor family, pairs with CD94 to form a heterodimer, acting as an inhibitory receptor that plays a critical role in regulating the cytotoxic activity of CD4^+^ CTLs.^44,46^ In contrast, NKG2D, a prominent member of the killer cell-activating receptor (KAR) family, enhances cytotoxicity and cytokine production in CD4^+^ CTLs. Upon ligand binding, NKG2D not only promotes cell proliferation but also ensures cell survival, which is essential for sustaining the immune response over time. NKG2D^+^ CD4^+^ T cells express key cytotoxic factors, including perforin, granzyme B, and Fas ligand (FasL). These factors enable them to effectively target and eliminate Treg cells in an NKG2D-NKG2DL-dependent manner.^43^ These findings suggest that CD4^+^ CTLs not only share functional similarities with NK cells but also may have a common origin, raising intriguing questions about the differentiation of immune cells and the plasticity of immune responses.

While substantial evidence supports the idea that CD4^+^ CTLs are closely associated with NK cells, the absence of classical NK cell markers, such as CD16 and CD56, indicates that CD4^+^ CTLs belong to the same lineage as conventional CD4^+^ Th cells. CD4^+^ T cells are activated upon interaction with peptide-MHC class II complexes, which are presented by APCs, accompanied by additional costimulatory signals and cytokine signaling. This interaction triggers downstream signaling cascades that drive the differentiation and functional specialization of CD4^+^ T cells. Upon activation, CD4^+^ T cells differentiate into several distinct subsets, including Th1, Th2, Th17, and Treg cells,^2^ each of which are characterized by unique surface molecule expression profiles, specific cytokine production patterns, and key transcription factors. Some researchers have proposed that CD4^+^ CTLs represent a novel subset within the broader category of CD4^+^ T cells. These researchers argue that, like other CD4^+^ T-cell subtypes, CD4^+^ CTLs should be identifiable on the basis of distinct markers or transcription factors.^45^ However, identifying specific markers for CD4^+^ CTLs remains challenging. While progress has been made in characterizing some of their functional properties, a reliable set of biomarkers to distinguish CD4^+^ CTLs from other T-cell subsets is still lacking. A recent study revealed that a subset of human former Treg (exTreg) cells exhibited cytotoxicity, which was characterized by the expression of CD3, CD4, CD16, and CD56. This combination of markers is commonly found on both T cells and NK cells, suggesting that exTreg cells may possess features of both adaptive and innate immune cells. However, bulk RNA sequencing analysis of sorted exTreg cells revealed that these cells were best classified as inflammatory or cytotoxic CD4^+^ T cells rather than as NK cells or traditional Treg cells, but these cells did not express specific markers.^47^ The lack of specific markers emphasizes the need for further research to delineate the precise nature of these cells and their role in immune regulation and disease.

Rather than data arising from NK cell precursors, accumulating data increasingly support the notion that CD4⁺ CTLs are a specialized subset within the CD4⁺ T-cell family. Recent studies have traced their origin to conventional CD4⁺ Th cells, including Th0,^23^ Th1,^21^ Th2,^22^ Tfh,^24^ and Treg^25^ cells, with Th1-like cells being the most prevalent source. This differentiation process reveals the adaptive nature of the immune system, where conventional helper T cells can acquire novel, effector-like properties in response to environmental cues. This differentiation trajectory now represents the dominant hypothesis, particularly supported by advances in single-cell transcriptomics and lineage-tracing techniques. Recent studies have identified distinct effector and precursor populations of CD4⁺ CTLs within the CD45RA-expressing effector memory CD4⁺ T-cell subset, characterized by elevated IL-7R expression and substantial clonal expansion during disease progression.^34^ IL-7, a key cytokine for T-cell development and survival, also promotes Th1 differentiation and enhances IFN-γ production.^48^ The upregulation of IL-7R in CD4⁺ CTLs may thus serve as both a marker and a driver of their differentiation, expansion, and functional specialization, suggesting potential utility in clinical identification. Single-cell TCR sequencing of bone marrow–derived CD4⁺ T cells from patients with multiple myeloma revealed significant clonal overlap between CD4⁺ CTLs expressing terminal differentiation markers and both Th1 and Th1-like CTL populations. Trajectory analysis further delineated a continuous differentiation pathway in which CD4^+^ CTLs originate from Th1 cells through an intermediate Th1. CTL state. Moreover, the marked enrichment of TCR signaling–related genes within both the CD4⁺ CTL and Th1 clusters supports the hypothesis that chronic antigenic stimulation drives the differentiation of CD4⁺ T cells into cytotoxic effectors.^49^ Building upon these findings, pseudotime trajectory analysis further revealed that CD4⁺ CTLs are predominantly positioned at later pseudotime stages, indicating clear temporal separation from noncytotoxic clusters. These findings suggest that CD4^+^ CTLs constitute a terminally differentiated subset that has undergone extensive differentiation and functional specialization in response to chronic antigenic stimulation. CD4^+^ CTLs exhibit significant expansion in supercentenarians, accompanied by a pronounced shift in T cells toward more differentiated states. The increased differentiation of T cells in these individuals may represent an adaptive strategy to sustain immune responses against infections and tumors over an extended lifespan.^50^

Interestingly, CD4^+^ CTLs have the ability to establish a distinct proinflammatory microenvironment, which plays a key role in immune modulation. Through the secretion of various cytokines, such as IFN-γ and TNF-α, CD4^+^ CTLs promote the proliferation and activation of conventional Th cells. Recent evidence indicates that when Th cells acquire cytotoxic features, they can profoundly influence the proliferation, differentiation, and phenotype of neighboring conventional Th cells. For example, these cytotoxic Th cells can stimulate Th17 polarization, a process critical for inflammatory responses and autoimmunity. Furthermore, within this microenvironment, CD4^+^ CTLs acquire resistance to Treg cells and the ability to suppress potentially pathogenic Th cells, collectively facilitating the accumulation of CD4^+^ CTLs in pathological states.^51^

Consequently, some researchers argue that CD4^+^ CTLs may not be easily distinguishable or identifiable via traditional marker proteins or transcription factors, as is typically the case for other T-cell subsets. This poses a significant challenge in both basic and clinical immunology, as it limits the ability to identify and study these cells specifically. Therefore, their identification may require a functional approach that focuses on their cytotoxic activity and the specific cytokines they produce rather than relying solely on surface markers.

### Biomarkers of CD4^+^ CTLs

Currently, there is no single, universally accepted marker that can be exclusively used to identify CD4^+^ CTLs. This presents a significant challenge in the study and clinical targeting of these cells. Several studies have utilized a combination of various surface markers and transcription factor expression patterns to define CD4^+^ CTLs. However, owing to the inherent heterogeneity within this subset, not all CD4^+^ CTLs express the same set of markers. Indeed, both the markers and the functions of CD4^+^ CTLs can vary significantly depending on their developmental stage, the specific immune microenvironment they inhabit, and the disease states in which they are involved. Understanding this heterogeneity is essential for developing precise methods to identify and target CD4^+^ CTLs in various immune contexts.

As previously discussed, CD4^+^ CTLs can arise from other classic CD4^+^ Th-cell subsets, and as a result, they share several common surface markers with these classical CD4^+^ T cells. These markers, such as CD3, CD4, and ICAM-1, are integral to the physiological functions of T-cell signaling, activation, and chemotaxis, which are crucial for T-cell-mediated immune responses.^52^ However, under certain pathological conditions, CD4^+^ CTLs typically exhibit characteristics associated with other T-cell subsets. For example, a study revealed that in patients with type 2 diabetes, the genes encoding the γδ TCR constant chains TRGC2 and TRDC were significantly enriched within a cluster of CD4^+^ CTLs.^53^ These genes are typically associated with γδ T cells, which are known for their ability to recognize nonpeptide antigens and provide rapid responses during infection. Moreover, CD4^+^ CTLs found in intraepithelial lymphocytes (IELs) of the intestine have been shown to express CD8αα homodimers, which are typically associated with CD8^+^ T cells, further demonstrating the plasticity and adaptability of CD4^+^ CTLs in different immune environments.^54^ This finding underscores the dynamic nature of CD4^+^ CTLs and their ability to adapt to different immune environments in response to various diseases.

Like that of CD4^+^ Th cells, the activation of CD4^+^ CTLs relies on the collaboration of several signaling pathways. These pathways include TCR-mediated recognition of antigen-presenting molecules, costimulatory signals, and cytokine signaling, all of which are critical for the initiation of immune responses. CD4^+^ CTLs recognize MHC class II molecules via their TCRs, which enables them to support CD8^+^ CTLs by enhancing their cytotoxic function, providing cytokine support, and ensuring the maintenance of an optimal immune microenvironment. However, CD4^+^ CTLs are predominantly enriched in the T_EMRA_ subset, which downregulates key costimulatory receptors such as CD27 and CD28. This subset is considered a highly differentiated population of T cells that have undergone extensive antigenic exposure, reflecting their terminal differentiation status. Nevertheless, CD4^+^ CTLs within the T_EMRA_ subset retain their cytotoxic activity, allowing them to respond rapidly to previously encountered pathogens or tumor cells, thereby demonstrating the long-term immune surveillance and memory capabilities of CD4^+^ CTLs.^34^ In earlier studies of CD4^+^ CTLs, these cells were often identified by their CD4^+^ CD28^-^ phenotype.^14,55^ This phenotype was initially thought to be a hallmark of fully differentiated, cytotoxic CD4^+^ T cells, distinguishing them from conventional CD4^+^ T cells that typically express CD28. However, recent studies have suggested that the loss of CD28 expression may not be a definitive marker for CD4^+^ CTLs, as certain stimuli, such as IL-12, can rescue CD28 expression even in senescent CD4^+^ T cells.^56^ These findings underscore the need for further investigation into the dynamic regulation of costimulatory molecules and their role in the differentiation and activation of CD4^+^ CTLs. Furthermore, several signaling receptors, including OX40, 4-1BB, and CRTAM, play crucial roles in the development of the cytotoxic functions of CD4^+^ CTLs. These receptors, in combination with CD4-TCR signaling, help promote the differentiation and activation of CD4^+^ CTLs, enabling them to perform their cytotoxic functions more efficiently.^57–60^

In contrast to CD4^+^ Th cells, the most distinguishing feature of CD4^+^ CTLs is the expression of various cytotoxic molecules, including GZMA, GZMB, GZMK, GNLY, PRF1, TNF-α, IFN-β, TRAIL, FAS, and CD107a, similar to those found in CD8^+^ CTLs.^34,50,53,61^ The similarity between CD4^+^ CTLs and CD8^+^ CTLs highlights their convergent evolution, as both subsets share the key cytotoxic machinery essential for immune defense. These cytotoxic molecules are critical for the effector functions of CD4^+^ CTLs, enabling them to directly kill infected or tumor cells, further distinguishing them from their helper counterparts. Recent advancements in single-cell transcriptomic analysis have provided compelling evidence for the expression of these cytotoxic markers in CD4^+^ CTLs, further validating their distinct role in immune responses.^62,63^ The ability to identify these markers at the single-cell level allows for a more precise characterization of CD4^+^ CTLs. This molecular profiling highlights the unique functional characteristics of CD4^+^ CTLs. These cells are not simply passive responders to immune signals. Instead, they actively engage in immune defense by acquiring cytotoxic functions that are typically associated with CD8^+^ T cells.

Generally, factors that are expressed in NK cells and associated with cytotoxic activity can also serve as useful markers for characterizing CD4^+^ CTLs. For example, NK cell-associated surface receptors such as KIR2DS2, NKG2A, and NKG2D can also be found on the surface of CD4^+^ CTLs.^41,43,44^ These surface receptors enable CD4^+^ CTLs to recognize not only antigens presented by MHC class II molecules but also a broader range of pathogen-associated signals, increasing their responsiveness to various threats. Furthermore, these receptors play crucial roles in the fine-tuning of CD4^+^ CTL activation, ensuring that these cells can effectively carry out their cytotoxic functions while maintaining immune homeostasis. In addition, CD4^+^ CTLs express CD11b and CD57, both of which are typically found on the surface of NK cells but not on conventional CD4^+^ T cells.^64,65^ Moreover, single-cell transcriptomic analysis revealed that NKG7, which is always expressed by NK cells, is also specifically enriched in CD4^+^ CTLs, concomitant with transcripts for granzyme B, perforin and granulysin in cells that display high cytotoxic potential.^34^ The coexpression of these cytotoxic markers further underscores the ability of CD4^+^ CTLs to engage in targeted cell killing, much like NK cells do. This molecular profiling not only aids in identifying CD4^+^ CTLs but also highlights the link between CD4^+^ CTLs and NK cells, bridging the gap between innate and adaptive immunity.

**Fig. 2:**
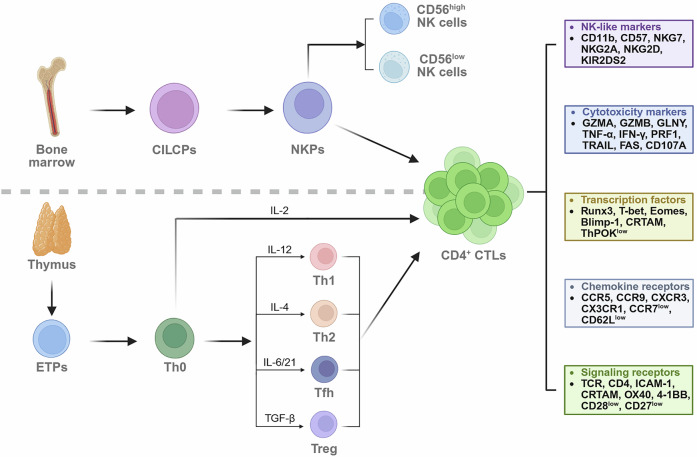
Origin, differentiation pathways and biomarkers of CD4^+^ CTLs. There are currently two main hypotheses regarding the origin of CD4^+^ CTLs: one hypothesis suggests that they may originate from natural killer (NK) cell precursors, whereas the other hypothesis proposes that they arise from other helper CD4^+^ T cells. Phenotypically, CD4^+^ CTLs share similarities with both of these cell types, as they express T-cell-related signaling molecules and NK-like markers. Additionally, certain transcription factors, cytotoxic molecules, and chemokine receptors can help identify these cells. However, owing to the heterogeneity of CD4^+^ CTLs and the lack of specific markers, their identification still relies on the combination of multiple markers. ETPs early thymic progenitors, CILCPs common innate lymphoid cell precursors, NKPs natural killer cell precursors, Tfhs T follicular helper cells, Tregs regulatory T cells, TGF-β transforming growth factor beta. Created at https://BioRender.com

Chemokine receptors play pivotal roles in guiding CD4^+^ CTLs to sites of tissue damage or infection, where they can quickly respond and contribute to immune responses. Several chemokine receptors, such as CCR5, CXCR3, CX3CR1, and CCR9, have been used to define CD4^+^ CTLs, enabling CD4^+^ CTLs to perform their cytotoxic and regulatory functions with precision.^35,66,67^ Importantly, CXCR6 has been identified as a critical receptor that helps define distinct therapeutic subtypes of CD4^+^ CTLs. This receptor is particularly important in the context of adoptive cell transfer therapy for pediatric B-ALL, where it plays a key role in determining the efficacy of the therapeutic approach.^68^ In contrast, the expression of lymph node homing receptors such as CD62L and CCR7 is downregulated in CD4^+^ CTLs.^69^ This downregulation indicates the transition of CD4^+^ CTLs into highly differentiated effector cells, which no longer need to circulate through lymphoid tissues for activation. These findings also highlight their progression toward a terminal differentiation state, reflecting the adaptation of CD4^+^ CTLs to their effector functions in peripheral tissues. These markers collectively aid in distinguishing CD4^+^ CTLs in research, but identifying specific markers that are exclusive to CD4^+^ CTLs remains a major focus and challenge for future studies (Fig. 2).

### Signaling and transcriptional network of CD4^+^ CTLs

The differentiation and function of CD4^+^ CTLs are tightly regulated by a complex interplay between extracellular signaling molecules and key transcription factors. Extracellular mediators are crucial in activating CD4^+^ CTLs and guiding their differentiation into effector cells. These signals are integrated at the cellular level to orchestrate the functional specialization of CD4^+^ CTLs in immune responses. Furthermore, various transcription factors play key roles in regulating gene expression programs that define the identity, differentiation, and cytotoxic capacity of CD4^+^ CTLs. The coordinated action of extracellular signals and transcriptional regulation ensures that CD4^+^ CTLs can effectively perform their immune functions, contributing to both the eradication of pathogens and the maintenance of immune homeostasis.

#### Extracellular mediators

T-cell activation generally depends on two distinct signals, which are essential for initiating an effective immune response. The first signal arises when the TCR interacts with a specific peptide that is presented by an MHC class II molecule on the surface of an APC.^70^ This interaction is essential for the initial recognition of the antigen by the T-cell and for the subsequent activation of the T cell, which triggers downstream immune processes. The affinity between the TCR and the peptide antigen, as well as the antigen dose, are critical factors that regulate the strength and duration of signal activation. These factors subsequently influence the development of cytotoxic functions in T cells.^71,72^ Together, these factors determine the efficiency and specificity of the immune response, which directly impacts the overall effectiveness of the immune system.

When the TCR engages with the peptide antigen presented by MHC molecules on APCs, a complex cascade of events is triggered. This leads to the spatiotemporal reorganization of the TCR, integrins, costimulatory receptors, and other signaling molecules, which then form a specialized structure known as the immunological synapse.^73^ This process facilitates stable and sustained contact between the T-cell and the APC, ensuring that immune signals are effectively transmitted. Furthermore, the formation of immunological synapses activates critical T-cell signaling pathways, which are necessary for initiating and amplifying the immune response.^74^ In addition, cytotoxic immunological synapses also play a role in triggering the apoptosis of Fas-expressing target cells by translocating FasL molecules from the lysosome to the cell membrane, as well as in the directional release of cytotoxic granules.^75,76^ Unlike the long-lasting and stable immunological synapses observed in conventional CD4^+^ Th cells, CD4^+^ CTLs form more transient and dynamic synapses, resembling those of CD8^+^ CTLs.^77^ This difference in synapse stability may reflect the distinct functional requirements of CD4^+^ CTLs, which need to rapidly exert their cytotoxic effects rather than maintain prolonged interactions with target cells. However, CD4^+^ CTLs are more prone to disrupting their own synapses, which can result in the escape of cytotoxic molecules from the synaptic interface.^78^ This may be one of the reasons why CD4^+^ CTLs exhibit less cytotoxic efficacy than CD8^+^ CTLs do and may also contribute to the spread of inflammation and bystander killing.

Notably, CD4^+^ CTLs typically express low levels of CD28, which is a key receptor required for the second signal in T-cell activation. Despite their low CD28 expression, CD4^+^ CTLs can still receive crucial second signals through alternative costimulatory pathways, especially under various pathological conditions. These alternative pathways allow CD4^+^ CTLs to bypass the traditional costimulatory requirement, enabling them to remain functionally competent in environments where conventional activation signals may be disrupted. For example, in patients with giant cell arteritis, CD4^+^ CTLs highly express NKG2D, a receptor that recognizes MHC class I chain-related gene A (MICA) enriched in the temporal arteries. This interaction between NKG2D and MICA enhances the costimulatory activity of CD4^+^ CTLs, further amplifying their immune response.^79^ In patients with melanoma, the activation of SLAMF7 has been shown to significantly increase the cytotoxicity of tumor-specific CD4^+^ T cells. This receptor contributes to the activation and functional priming of CD4^+^ CTLs, enabling them to target and eliminate tumor cells more effectively.^80^ Moreover, in acute coronary syndrome patients, CD4^+^ CTLs show high expression levels of OX40 and 4-1BB, with their ligands not only present in plaques but also expressed on monocytes in circulation, collectively promoting the establishment of cytotoxic functions.^81^ This expression pattern collectively promotes the establishment of cytotoxic functions in CD4^+^ CTLs, further enhancing their immune responses in several diseases.

The specificity of TCR binding ensures that the response is directed against cells presenting the corresponding antigen, thus facilitating a targeted immune response. Notably, emerging evidence suggests that T cells can acquire cytotoxic functionality through exposure to proinflammatory cytokines (e.g., IL-2, IL-12, and IL-15) independently of canonical antigen recognition.^82–84^ This antigen-nonspecific activation pathway may enable rapid and broad-spectrum immune responses, particularly in scenarios requiring immediate defense against infections or tumors without prior antigen priming. However, whether CD4^+^ CTLs can mount analogous responses remains incompletely understood. Current evidence suggests that the activation of CD4⁺ CTLs is predominantly TCR dependent but requires the involvement of multiple cytokines to achieve full functional activation.

A special cytokine microenvironment is essential for unlocking the full cytotoxic potential of CD4^+^ CTLs, as these cytokines play a central role in driving the activation, differentiation, and function of these cells. Among the pivotal cytokines, IL-2 is indispensable for the regulation of immune responses, especially in the expansion and differentiation of effector T cells. Moreover, IL-2 is instrumental in inducing peripheral tolerance, primarily by promoting the function and expansion of Tregs.^85^ The concentration of IL-2 and the high-affinity IL-2 receptor are essential for inducing the Th1 phenotype and the expression of cytotoxic molecules in CD4^+^ T cells in vivo. Furthermore, IL-2 induces FasL/Fas-mediated cytotoxicity in a dose-dependent manner. This cytotoxic response is antigen independent and not restricted by MHC molecules.^86^ IL-7, another crucial cytokine, plays a fundamental role in the differentiation and survival of naive T cells, ensuring that these cells can respond to future antigenic challenges. Moreover, IL-7 is essential for the generation and maintenance of memory T cells, which are pivotal for long-term immunity and rapid responses to reinfection. Similarly, in CD4^+^ CTLs, IL-7 regulates their proliferation, likely through the activation of the JAK-STAT signaling pathway, ensuring that CD4^+^ CTLs can expand in response to immune challenges.^87^ Furthermore, IL-15 is essential for regulating various aspects of T-cell biology, including activation, effector function, tissue residency, and senescence. It acts as a key driver in initiating TCR-independent activation of T cells, allowing these cells to become functionally active even in the absence of direct antigen recognition. In addition, IL-15 increases the expression of CD69, a marker of T-cell activation, and stimulates the production of IFN-γ, which is critical for their cytotoxic functions. In addition to these effects, IL-15 facilitates the differentiation of CD4^+^ Th cells into CD4^+^ CTLs, thereby increasing the pool of cytotoxic T cells capable of responding to infections or tumors.^36,88^

In summary, the coordinated interaction of various extracellular mediators collectively facilitates the establishment of the cytotoxic phenotype in CD4^+^ CTLs. The intricate network of cytokines, receptors, and signaling molecules ensures that CD4^+^ CTLs can rapidly respond to infections, tumors, and other immune challenges.

#### Key transcription factors

The integration and interpretation of numerous cellular and environmental factors are orchestrated by transcription factors. Various transcriptional regulators have been identified as key players in shaping the differentiation program of CD4^+^ CTLs, guiding their development from naive T cells to fully differentiated cytotoxic T cells. Understanding the molecular mechanisms by which these transcriptional regulators operate is essential for unraveling the full potential of CD4^+^ CTLs in immune responses.

The differentiation of CD4^+^ and CD8^+^ T cells in the thymus is tightly regulated by ThPOK and Runx3, with ThPOK promoting the development of CD4^+^ T cells and Runx3 driving the differentiation of CD8^+^ T cells. Notably, each transcription factor inhibits the expression of the other, ensuring that the development of one T-cell subset does not interfere with the other, maintaining the proper balance between CD4^+^ and CD8^+^ T cells in the immune system.^89^ Postthymic CD4^+^ T cells typically maintain ThPOK expression, which helps preserve lineage stability by inhibiting Runx3.^90,91^ The ectopic expression of ThPOKs in CD8^+^ T cells disrupts their normal differentiation and functional programming by downregulating the expression of key molecules such as Eomes, IFN-γ, granzyme B, and perforin. This results in a marked reduction in their cytotoxic activity.^92,93^ Conversely, following TCR signaling activation, Runx3 induces the expression of IFN-γ and upregulates the expression of granzyme B. Subsequently, Runx3 collaborates with the transcription factors Eomes and T-bet to further increase the production of cytotoxic molecules, even within CD4^+^ T cells.^94,95^ This process is a key step in the differentiation of CD4^+^ T cells into CD4^+^ CTLs, enabling them to acquire the capacity to directly target and eliminate infected or tumor cells.

Interestingly, multiple studies have shown that the upregulation of Runx3 in CD4^+^ CTLs under various pathological conditions is closely associated with the acquisition of cytotoxicity. In the small intestine, a subset of CD4^+^ IELs expressing CD8αα can downregulate the expression of ThPOK and upregulate Runx3 expression in response to dietary and microbial stimuli. This adjustment enhances their cytotoxic potential, allowing them to function as effector cells capable of combating pathogens and maintaining immune homeostasis within the gut.^37,96,97^ This adaptive change underscores the remarkable plasticity of CD4^+^ T cells in different immune environments. During human cytomegalovirus (CMV) infection, even though ThPOK expression remains intact, CD4^+^ CTLs exhibit a transcriptional profile that is enriched in genes typically associated with CD8^+^ T cells. Prolonged activation of naive CD4^+^ T cells with Th1-polarizing cytokines induces perforin-dependent cytotoxic activity, which is dependent on Runx3 and constrained by ThPOK.^61,98^ This adaptability highlights the dynamic nature of T-cell differentiation in response to persistent viral infections. Importantly, Runx3 is indispensable for the differentiation of CD4^+^ CTLs. Studies have shown that the deletion of Runx3 in differentiated CD4^+^ T cells effectively prevents the induction of CD4^+^ CTLs, blocking their ability to acquire cytotoxic functions. However, this deletion does not affect the differentiation of Th1 cells.^99^ These findings underscore that the coordinated action of these two transcription factors is crucial in determining the functional capabilities of CD4^+^ CTLs. Furthermore, they demonstrated the remarkable plasticity of CD4^+^ and CD8^+^ T-cell lineage identities, suggesting that T cells are not strictly confined to a single functional role. Instead, T cells can adapt and modify their functional roles on the basis of environmental factors, signaling cues, and activation conditions.

T-bet influences the secretion of IFN-γ and the expression of perforin and granzyme B genes in CD8^+^ CTLs. Additionally, T-bet coordinates various signaling pathways that direct the development and differentiation of CD4^+^ CTLs, ensuring that these cells acquire the necessary characteristics to perform their immune functions effectively.^100^ T-bet is particularly important as a master transcription factor for committing T cells to the Th1 lineage. Its expression is tightly regulated by upstream signaling pathways, including the IL-2 pathway and the STAT2-dependent type I interferon signaling pathway.^101,102^ In the absence of STAT2, a critical component of the type I interferon signaling pathway, the expression of both T-bet and granzyme B is significantly reduced, reflecting the importance of this signaling cascade in immune responses.^103^ Furthermore, CD4^+^ T cells that lack T-bet exhibit a significant reduction in granzyme B production, emphasizing the critical role that T-bet and associated signaling pathways play in the generation and activation of CD4^+^ CTLs.^104^ In addition, T-bet can induce the expression of Runx3 in CD4^+^ T cells, which plays a vital role in further regulating the expression of critical cytotoxic molecules, including FasL and perforin.^100,105^ Through the combined action of T-bet, Runx3, and other signaling pathways, Th1 cells acquire the ability to kill target cells, making them crucial components of the immune defense system.

Similarly, Eomes, another key transcription factor in the T-box family, collaborates with T-bet to regulate the differentiation and cytotoxic function of CD4^+^ CTLs. Signaling pathways activated by costimulatory molecules such as CRTAM, OX40 (CD134), and 4-1BB (CD137) serve as upstream signals to promote Eomes expression in CD4^+^ CTLs.^58–60^ Additionally, ThPOK acts as a negative regulator of Eomes, and the suppression of ThPOK expression has been shown to lead to an increase in Eomes levels. ThPOK typically plays a crucial role in maintaining the identity of CD4^+^ T cells, and its inhibition facilitates the expression of Eomes. This, in turn, ensures that CD4^+^ CTLs acquire cytotoxic characteristics to effectively coordinate the overall immune response.^106^ Eomes has also been shown to induce the expression of the immunoregulatory cytokine IL-10, which plays a crucial role in maintaining immune homeostasis. By promoting IL-10 production, Eomes helps modulate the immune response, preventing excessive inflammation and tissue damage.^107,108^ The overexpression of Eomes in Th2 effector cells enhances the mRNA expression of perforin and granzyme B, thereby endowing Th2 cells with cytotoxic properties.^106^ This dual function of Eomes, in both enhancing cytotoxicity and promoting regulatory cytokine production, underscores its complex role in immune regulation.

In CD4^+^ T cells, the development of cytotoxic capabilities is also dependent on Blimp1, which promotes the binding capacity of T-bet to cytotoxicity-related gene promoters and increases its cytolytic potential.^109,110^ Blimp1 is a key regulator of Th2 lineage commitment, promoting the differentiation of Th2 cells while simultaneously limiting the differentiation of Th1, Th17, and Tfh cells. It also coordinates with other transcription factors to regulate the expression of IL-2, IL-21 and IL-10 in CD4^+^ T cells.^111^ The expression of Blimp1 in CD4^+^ T cells is primarily induced by upstream signals from the IL-2 and JAK/STAT signaling pathways, with IL-2 playing a crucial role, particularly through the activation of STAT5. These signals collectively activate Blimp1, which then drives the differentiation and cytotoxic functions of CD4^+^ CTLs, enhancing their capacity to participate in immune responses.^112^ IL-2 can relieve the antagonistic effect of Bcl6 on Blimp-1, thus enhancing the cytotoxic potential of CD4^+^ CTLs.^111^ Single-cell transcriptomic analysis has shown that CD4^+^ CTLs exhibit significantly higher expression levels of Blimp1 than other Th cell subsets do.^113^ This differential expression not only helps to distinguish CD4^+^ CTLs at the molecular level but also provides insights into the unique transcriptional program driving their cytotoxic differentiation. Moreover, Blimp1 is instrumental in driving the production of granzyme B, along with IL-2, and regulates the differentiation of CD4^+^ CTLs, a process that can be suppressed by Treg cells.^102^ In humans, the transcription factor homolog of Blimp1 in T cells, known as Hobit, plays a crucial role in identifying CD4^+^ CTLs. Hobit^+^ CD4^+^ T cells share many characteristics with Hobit^+^ CD8^+^ T cells, including the expression of key cytotoxic molecules, T-bet, and CX3CR1. This similarity highlights the functional overlap between CD4^+^ and CD8^+^ CTLs, suggesting that these cells may share similar differentiation pathways and immune roles.^35^

Upon activation, CRTAM, a surface receptor containing two Ig-like domains, is quickly and transiently expressed on NK cells and CD8^+^ T cells. It plays a crucial role in the adhesion, interaction, and migration of these cells by interacting with its ligand, Nectin-like 2.^114^ Promoter analysis of CRTAM has demonstrated that its expression is regulated by two key transcription factors, AP-1 and ZEB1. AP-1 serves as a positive regulator, promoting CRTAM expression, whereas ZEB1 acts as a negative regulator, inhibiting CRTAM expression.^115,116^ However, the precise mechanisms that regulate CRTAM expression during in vivo immune responses are still not fully understood. These processes may be influenced by various factors, including the mode of T-cell stimulation and the strength of the signaling pathways involved.^45^ Studies have shown that partially activated CD4^+^ T cells express CRTAM and that only CRTAM^+^ CD4^+^ T cells can differentiate into fully functional CD4^+^ CTLs.^58,110^ The ability of CRTAM to induce CD4^+^ CTL features upon overexpression depends on its cytoplasmic tails, suggesting that the cytoplasmic region of CRTAM likely acts as a key signaling hub that activates downstream molecular pathways involved in CD4^+^ CTL differentiation.^57^ Furthermore, when CRTAM-activated CD4^+^ T cells are cultured with IL-2, they express high levels of Eomes and efficiently generate CD4^+^ CTLs, indicating that CRTAM^+^ T cells may serve as precursors to CD4^+^ CTLs.^58^ Additionally, ectopic expression of CRTAM in CD4^+^ T cells can induce the production of IFN-γ and upregulate the expression of cytotoxicity-related genes. This enhances the cytotoxic capabilities of CD4^+^ T cells, thereby contributing to their effector functions.^117^ CRTAM-induced changes in T-cell polarity help these cells acquire specialized roles in immune responses, highlighting its importance in shaping T-cell effector functions.

Additionally, several other key molecules, such as T-cell factor 1 (TCF-1),^118^ cytotoxic T lymphocyte-associated protein 4 (CTLA-4),^24,119^ Aiolos (Ikaros zinc finger transcription factor),^120^ histone deacetylases 1 and 2 (HDAC1 and HDAC2, respectively),^121^ and IL-18R/MyD88 signaling,^122^ among others, can also impact the differentiation process of CD4^+^ CTLs. In subsequent sections, we delve into the mechanisms through which these molecules influence CD4^+^ CTL functions in various pathological conditions.

**Fig. 3:**
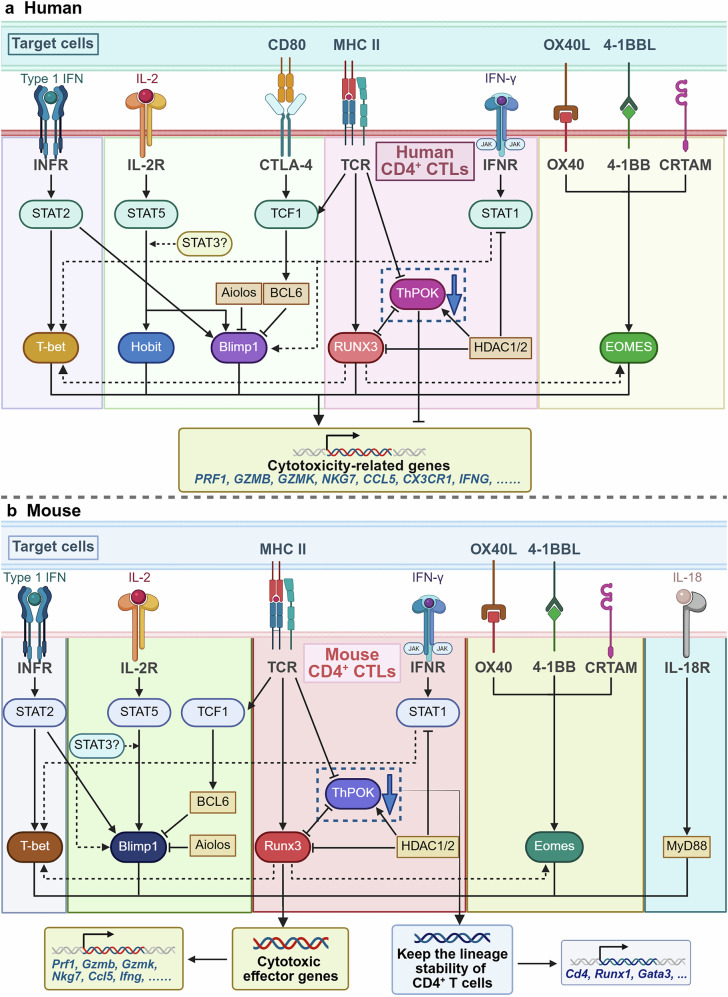
Signaling pathways and transcriptional regulatory network of CD4^+^ CTL differentiation. The differentiation of CD4^+^ CTLs is regulated by a complex network of signaling pathways and transcription factors in both humans (**a**) and mice (**b**). The balance between ThPOK and Runx3 is a key factor regulating the cytotoxicity of CD4^+^ CTLs. TCR signaling increases the level of Runx3 in CD4^+^ T cells, leading to the suppression of ThPOK, which maintains the stability of the CD4^+^ T-cell lineage. Runx3 is considered a critical regulator of cytotoxic gene expression in CD8^+^ T cells, as it can induce the expression of IFN-γ and upregulate the expression of granzyme B. Subsequently, Runx3 works in concert with Eomes and T-bet to further promote the production of cytotoxic molecules, even in CD4^+^ T cells. Additionally, multiple transcription factors and signaling pathways collectively contribute to the differentiation and functional development of CD4^+^ CTLs. Created at https://BioRender.com

In conclusion, the regulation of CD4^+^ CTL fate is governed by a highly complex and dynamic network of transcriptional interactions. Multiple factors—including persistent antigen stimulation, activation of TCR signaling, and cytokine cues within the local microenvironment—collectively influence the acquisition of the CD4⁺ CTL phenotype. Under various pathological conditions, factors such as aging, CMV serostatus, metabolic reprogramming, and exposure to microbiota-derived signals have also been shown to shape the differentiation and functional specialization of CD4⁺ CTLs, contributing to their pronounced heterogeneity. These diverse signals converge on core transcriptional and signaling pathways, ultimately determining the differentiation trajectory, effector function, and long-term persistence of CD4⁺ CTLs in response to distinct immunological challenges.^12,50,123–125^ The ongoing exploration of these networks is essential for understanding how CD4^+^ CTLs contribute to immune responses and how their functions can be modulated for therapeutic purposes (Fig. 3).

## Biological functions of CD4^+^ CTLs

**Fig. 4:**
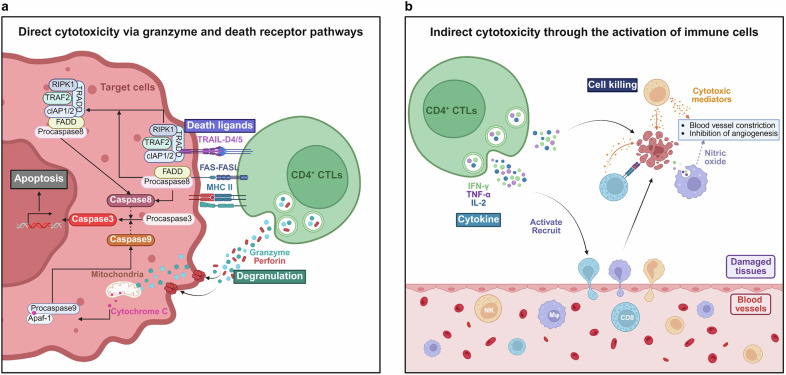
Biological functions of CD4^+^ CTLs. CD4^+^ CTLs exhibit both direct (**a**) and indirect (**b**) cytotoxic capabilities, similar to those of CD8^+^ CTLs and natural killer (NK) cells. Direct cytotoxicity involves two main mechanisms for inducing target cell death: the cytotoxic granule pathway and the death ligand pathway. Both pathways ultimately activate caspase-3, leading to apoptosis. In addition to direct cytotoxicity, CD4^+^ CTLs also secrete inflammatory cytokines such as IFN-γ and TNF-α, which recruit and activate other immune cells, including NK cells, macrophages, and CD8^+^ T cells. These recruited immune cells contribute to target cell death and mediate blood vessel constriction and the inhibition of angiogenesis by producing cytotoxic mediators and nitric oxide. Created at https://BioRender.com

CD4^+^ CTLs generally share functional and transcriptional characteristics with CD8^+^ CTLs and NK cells, exhibiting direct cytotoxic activities more akin to these effector cells than to the classical helper functions typically associated with CD4^+^ T cells. A distinguishing feature of CD4^+^ CTLs is their MHC II-restricted cytotoxicity, setting them apart from conventional CD4^+^ Th cells, which typically operate through the MHC II pathway to provide helper functions. Their cytotoxic mechanisms involve two primary pathways: the cytotoxic granule pathway and the death ligand pathway (Fig. 4).

The cytotoxic granule pathway involves the release of cytotoxic granules from CD4^+^ CTLs, which contain perforin and granzymes. This pathway is regarded as central to the cytotoxic function of CD4^+^ CTLs, enabling them to directly kill target cells through the delivery of these powerful effector molecules. A cytotoxic immunological synapse is formed between CD4^+^ CTLs and their target cells, where the TCR is centrally located within the synaptic structure.^126^ The polarized cytotoxic granules are strategically positioned at the plasma membrane in the secretory domain, which is adjacent to the central cluster of TCR molecules. Upon receiving specific signaling cues, the cytotoxic granules are rapidly released into the synaptic cleft, ensuring a high local concentration of cytotoxic proteins at the site of target cell interaction. This localized release minimizes the risk of collateral damage to surrounding healthy cells, ensuring that only the targeted infected or malignant cells are affected.^127^

Perforin oligomerizes and forms pores on the target cell membrane, allowing the proapoptotic granzymes to enter the target cell cytoplasm, where they cleave critical substrates to initiate apoptotic cell death.^128^ Granzyme B targets two key early molecules: BH3-interacting domain death agonist and procaspase-3. A truncated BH3-interacting domain death agonist disrupts the outer mitochondrial membrane, leading to the release of the proapoptotic factor cytochrome c. Once released from the intermembrane space, cytochrome c binds to APAF1 and recruits procaspase-9, forming the apoptosome. Within the apoptosome, caspase-9 is autocatalytically activated, and this activation leads to the cleavage and activation of caspase-3, which then drives the execution phase of apoptosis.^129,130^ These perforin^+^ and granzyme^+^ CD4^+^ T cells are essential in antiviral and tumor immunity and function by directly eliminating target cells. However, their cytotoxic response is generally slower than that of classic cytotoxic lymphocytes, such as CD8^+^ CTLs, highlighting differences in the kinetics of their immune response.^80,131,132^ Cytokines such as IL-2, IL-7, IL-15, and IL-18 can increase the CD4^+^ CTL response by reinforcing their commitment to the cytotoxic phenotype and upregulating mRNAs for perforin, granzymes A and B, interferon-γ, and Fas ligand.^91,103,122,133^ Recent studies have shown that VEGF-A enhances CD4^+^ CTL cytotoxicity through the VEGF-R1/VEGF-R2/AKT/mTOR signaling pathway, offering insights for novel treatment targets for CD4^+^ CTL-related disorders.^134^

Notably, perforin expression differs between CD4^+^ and CD8^+^ CTLs. In CD4^+^ CTLs, perforin expression is activation dependent, meaning that it is induced during immune responses. In contrast, memory CD8^+^ CTLs constitutively express perforin, which allows them to maintain their cytotoxic function even without continuous activation.^135^ Additionally, compared with CD4^+^ CTLs, CD8^+^ CTLs have a greater ability to produce granzyme B. However, this enhanced granzyme B production is more tightly regulated in CD8^+^ CTLs, ensuring that their cytotoxic activity is controlled and does not cause excessive damage to surrounding healthy tissues.^136^

The death ligand pathway includes both Fas/FasL-mediated apoptosis and TRAIL-mediated apoptosis. FasL expressed on the surface of CD4^+^ CTLs binds to Fas on the surface of target cells, activating an intracellular signaling cascade that involves the Fas-associated death domain and caspase-8. This cascade leads to the activation of caspase-3, ultimately inducing apoptosis in the target cell.^137,138^ In addition to Fas/FasL interactions, TRAIL expressed on the surface of CD4^+^ CTLs binds to TRAIL receptors on target cells. This interaction initiates a similar death signaling complex, which also leads to the activation of caspase enzymes and induces apoptosis in target cells. This alternative pathway enhances the cytotoxic capabilities of CD4^+^ CTLs, especially those of cells resistant to the Fas/FasL pathway.^139^ Interestingly, various subsets of CD4^+^ Th cells, which are traditionally known for their helper functions, also possess the potential to exhibit cytotoxic activity. This cytotoxic potential is particularly enhanced when Fas expression on target cells is induced following specific interactions with these Th cells.^140^ In various cancers and chronic infections, the death ligand pathway is a crucial mechanism through which CD4^+^ CTLs carry out their cytotoxic functions. The costimulatory receptor 4-1BB, when targeted with an agonistic antibody, can provide critical signals that reinforce the commitment of CD4^+^ T cells to their cytotoxic phenotype, thereby promoting their ability to kill infected or tumor cells directly.^4,141^

Interestingly, the cytotoxic mechanisms of CD4^+^ CTLs vary not only across different virus infections and types of malignancies but also within a single experimental model. For example, in an influenza virus model, the concentration of IL-2 and the antigen dose play key roles in determining whether CD4^+^ CTLs exhibit perforin-mediated or FasL-mediated cytotoxicity.^103^ This variability reflects the adaptability of CD4^+^ CTLs in response to diverse immune challenges, making them a versatile component of the immune system capable of tailoring their responses on the basis of the specific nature of the threat.

In addition to their cytotoxic functions, CD4^+^ CTLs also play crucial roles in shaping the inflammatory environment by secreting proinflammatory cytokines. Upon migrating to sites of inflammation, CD4^+^ CTLs secrete a broad spectrum of proinflammatory cytokines, including IL-2, IFN-γ, TNF-α, granulocyte‒macrophage colony‒stimulating factor (GM‒CSF), and macrophage colony‒stimulating factor (M‒CSF).^142–144^ These cytokines play crucial roles in coordinating the immune response, ensuring the effective recruitment and activation of other immune cells, such as NK cells, macrophages, and CD8^+^ CTLs, to sites of infection, injury, or inflammation. These cells subsequently contribute to target cell death, as well as blood vessel constriction and the inhibition of angiogenesis, by releasing cytotoxic mediators and nitric oxide.^145–147^ This action not only helps eliminate infected or malignant cells but also modulates the vascular response to injury or inflammation. Through these complex mechanisms, CD4^+^ CTLs not only indirectly exert cytotoxic effects but also play a vital role in regulating the immune response to inflammatory stimuli.

Moreover, despite expressing typical death-inducing receptors, CD4^+^ CTLs exhibit resistance to apoptosis regulated by Treg cells, which may contribute to the prolonged survival and persistence of these cells.^51,148^ This resistance to apoptosis in CD4^+^ CTLs could be a result of dysregulation of proapoptotic signaling pathways. The restoration of these signals, such as proapoptotic proteins and apoptotic receptors, would allow CD4^+^ CTLs to regain their typical sensitivity to apoptosis.^149^ In addition to the loss of CD28 receptor expression, CD4^+^ CTLs also fail to express the CD40 ligand (CD40L), which is crucial for initiating the signaling required for B-cell activation. As a result, CD4^+^ CTLs are unable to provide the necessary helper signals for B cells to produce antibodies effectively, limiting their role in humoral immunity.^31^

These functional characteristics distinguish CD4^+^ CTLs from conventional CD4^+^ Th cells and CD8^+^ T cells and suggest that this cell population may perform certain specialized physiological functions in vivo. To facilitate a clearer understanding of their unique properties, we compiled a comparative summary that systematically highlights the key differences in phenotypic markers, effector functions, transcriptional profiles, and immunological roles among CD4⁺ CTLs, conventional CD4⁺ Th cells, and CD8⁺ CTLs (Table 1).^150,151^ However, because their antigen recognition is less precise than that of CD8^+^ CTLs, the risk of unintended cell death is increased. This potential risk is thought to be controlled under normal physiological conditions through tight regulation of CD4^+^ T-cell development, preventing them from triggering harmful immune responses that could lead to immunopathology.^4,152^ In healthy young individuals, CD4^+^ CTLs typically make up a small fraction of the total CD4^+^ T-cell population, accounting for only 0.1% to 2.5% of the total population. However, as previously mentioned, under pathological conditions, CD4^+^ CTLs can undergo substantial clonal expansion, which allows for a more robust immune response.^34,153^ The exact distribution of CD4^+^ CTLs in healthy individuals remains unclear. In certain studies, CD4^+^ CTLs have been observed in the small intestinal lamina propria of healthy individuals, indicating that these cells may play a role in mucosal immunity and tissue surveillance.^37^ Recent studies have also isolated CD4^+^ CTLs from human peripheral blood and tonsils, although these cells are primarily found outside of germinal centers.^38^ However, more research is needed to fully understand the distribution of CD4^+^ CTLs in other tissues, as their roles in various organs remain largely unexplored.

**Table 1:** Characteristic features of CD4^+^ CTLs

Features	CD4^+^ CTLs	Conventional CD4^+^ T cells	CD8^+^ CTLs	References
Surface markers	CD4^+^, CD27/CD28^-^, NK-like receptors^+^, CD107a^+^, CRTAM^+^, CX3CR1^+^, CCR7^-^, CD62L^-^, CD40L^-^, ICOS^+^	CD4^+^, CD27/CD28^+^, CD107a^-^, CX3CR1^-^, CCR7^+/-^, CD62L^+/-^, CD40L^+^, ICOS^high^	CD8^+^, CD27/CD28^+/-^, NK-like receptors^+^, CD107a^+^, CRTAM^+^, CCR7^+/-^, CD62L^+/-^, ICOS^low^	^2,52,150^
Transcription factors	Runx3^high^, ThPOK^low^, T-bet^+^, Blimp1^+^, Hobit^+^, Eomes^+^	ThPOK^high^, Runx3^low^, T-bet^+^ (Th1), GATA3^+^ (Th2), RORγt^+^ (Th17), Bcl6^+^ (Tfh)	Runx3^high^, ThPOK^low^, T-bet^+^, Blimp1^+^, Eomes^+^	^35,50,57,89^
MHC restriction	MHC II restriction	MHC II restriction	MHC I restriction	^2^
Cytotoxic molecules	Perforin, Granzyme B, FasL, TRAIL	Not typically secreted	Perforin, Granzyme B, FasL, TRAIL	^23,45^
Cytotoxic capacity	Moderate cytotoxicity, more sustained, common in chronic diseases or as a complement to CD8+ CTLs’ function	Weak or absent	Strong cytotoxicity, prone to exhaustion in chronic infections or cancer	^4,151^
Cytokine profile	IFN-γ, TNF-α, IL-2, M-CSF, GM-CSF	IFN-γ, TNF-α, IL-2 (Th1); IL-4, IL-5, IL-13 (Th2); IL-17 (Th17); IL-4, IL-21 (Tfh)	IFN-γ, TNF-α, IL-2	^142,143^
Proliferative potential	Moderate, less robust than CD8⁺ CTLs, but more sustained in chronic responses	High, capable of robust clonal expansion upon antigen stimulation	Strong proliferative burst during acute responses, but susceptible to exhaustion in chronic settings	^2,4^
Sensitivity to apoptosis	Low, relatively resistant to apoptosis, especially in chronic inflammation	High, sensitive to activation-induced cell death	High, prone to apoptosis and functional exhaustion under persistent antigen stimulation	^149^
Treg suppression sensitivity	Low; relative resistance to Treg-mediated suppression due to CD28 loss and reduced expression of pro-apoptotic molecules	High, readily suppressed by Tregs to maintain immune tolerance	Low, relative resistance to Treg-mediated suppression, with context-dependent modulation	^51,148^
Ability to provide help signals to B cells	Limited, linked to CD40L deficiency	Strong, particularly Tfh subset, via IL-21, IL-4, CD40L, essential for germinal center reactions	Minimal, generally lack direct B cell help capacity	^31^

Compared with those in young individuals, CD4^+^ CTLs characterized by the expression of NKG2D, granzyme B, and perforin are significantly enriched in elderly individuals and are associated with cognitive impairment in older individuals.^154,155^ Importantly, a remarkable expansion of these cells is observed in supercentenarians, in whom CD4^+^ CTLs account for 15 to 35% of the total CD4^+^ T-cell population. Recent studies have shown that CD4^+^ CTLs can directly target and eliminate senescent cells by recognizing reactivated CMV antigens. This ability highlights their role in immune surveillance, particularly in removing aged or damaged cells, and suggests their involvement in age-related immunity.^156^ Therefore, these unique lymphocytes may be crucial adaptations for achieving extraordinary longevity, as they play a significant role in maintaining immune responses to infections and diseases, contributing to immune system resilience over time.^50,157^

## CD4^+^ CTLs in diseases

**Fig. 5:**
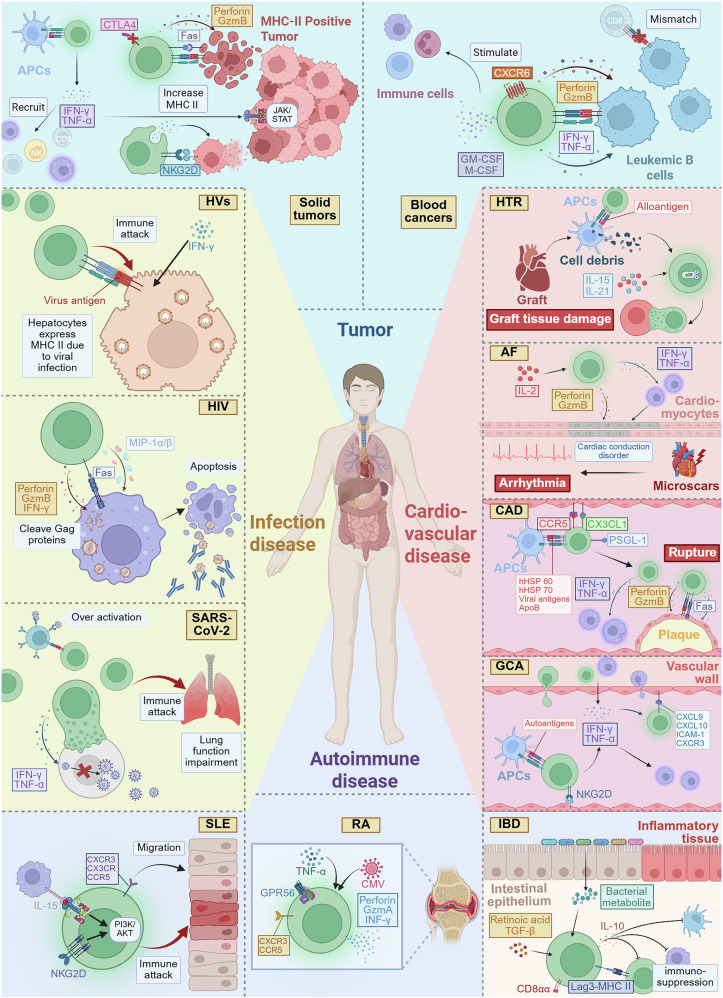
CD4^+^ CTLs in various diseases. CD4^+^ CTLs play pivotal roles in various disease contexts. In cancer, CD4^+^ CTLs assist the immune system in recognizing and eliminating tumor cells, particularly those expressing MHC class II molecules, as these cells often evade clearance by CD8^+^ CTLs through immune escape. The unique feature of CD4^+^ CTLs helps fill this functional gap. In infectious diseases, CD4^+^ CTLs serve as a key defense mechanism against both viral and bacterial pathogens, using their cytotoxic capabilities to clear infected cells and help control the spread of infection. However, in autoimmune diseases, the activation of CD4^+^ CTLs can become dysregulated, leading to excessive tissue damage and inflammation, thus worsening the progression and severity of the disease. In cardiovascular diseases, the activation of CD4^+^ CTLs may lead to immune-mediated myocardial or vascular tissue damage, resulting in functional abnormalities and pathological damage. MICA MHC class I chain-related protein A, GM-CSF granulocyte‒macrophage colony‒stimulating factor, M-CSF macrophage colony‒stimulating factor, GPR56 G protein‒coupled receptor 56, HVs hepatitis viruses, HIV human immunodeficiency virus, SARS-CoV-2 severe acute respiratory syndrome coronavirus 2, HTR heart transplant rejection, AF atrial fibrillation, CAD coronary artery disease, GCA giant cell arteritis, SLE systemic lupus erythematosus, RA rheumatoid arthritis, IBD inflammatory bowel disease. Created at https://BioRender.com

Recent studies have increasingly recognized the multifaceted role of CD4^+^ CTLs in the pathogenesis and progression of various diseases. These cells are involved not only in tumors but also in a variety of infectious diseases, autoimmune disorders, and cardiovascular diseases. CD4^+^ CTLs contribute to immune responses, influencing disease outcomes through their cytotoxic activities and immune modulation. This section explores the diverse contributions of CD4^+^ CTLs across these diseases, highlighting their complex functions in immunity and disease progression (Fig. 5).

### Tumors

#### Solid tumors

Tumors can be defined as complex, systemic diseases that not only affect the site of tumor origin but also involve a wide array of systemic factors, including immune responses, metabolic changes, and inflammation.^158^ Solid tumors and hematological cancers exhibit distinct physical and physiological characteristics, which results in fundamentally different pathogeneses and therapeutic approaches.^159^ Solid tumors, defined as malignant neoplasms of nonlymphatic origin, develop within deep tissue sites and interact dynamically with their surrounding environment, collectively known as the TME. The TME, which comprises immune cells, cancer-associated fibroblasts, endothelial cells, and other stromal elements, plays a pivotal role in tumor progression and immune evasion. Its cellular composition and functional state are shaped by the tumor’s tissue of origin, intrinsic cancer cell features, disease stage, and patient-specific factors.^160^ Immune cells within the TME perform dual physiological functions: on the one hand, they can actively surveil and eliminate newly transformed tumor cells through immune monitoring; on the other hand, influenced by the TME, they may also foster an immunosuppressive environment that enables tumor escape.^161,162^ A deeper understanding of the interactions between the immune system and the TME can provide valuable insights into the development of novel therapeutic strategies for combating cancer.

Compared with that of infectious diseases, the role of CD4^+^ CTLs in tumor immunity has not been fully recognized and appreciated until recently. The first study in 1999 demonstrated that antigen-specific Th1 and Th2 cells, in collaboration with CD8^+^ CTLs, were able to clear established tumors in ovalbumin-specific T-cell receptor transgenic mice, showing their cytotoxic potential.^163^ Subsequent studies revealed that tumor-specific CD4^+^ T cells could eliminate tumors that were resistant to CD8-mediated rejection, highlighting their potent tumor-clearing capabilities.^164^ In 2008, Hunder and colleagues developed an in vitro method to isolate and expand autologous CD4^+^ T-cell clones specific to the melanoma-associated antigen NY-ESO-1.^165^ This method led to sustained clinical remission in patients, underscoring the clinical potential of CD4^+^ CTLs as a therapeutic strategy. Later, in a mouse melanoma model, adoptive transfer of CD4^+^ T cells expressing a transgenic T-cell receptor specific for the melanoma antigen TRP-1 resulted in unique cytotoxic activity and the eradication of established melanoma.^33,166^ In addition to melanoma, CD4^+^ CTLs have also been identified in various other solid tumors, including non-small cell lung cancer,^167^ colorectal cancer,^168^ bladder cancer,^169^ oral squamous cell carcinoma,^170^ and breast cancer.^171^ Collectively, these studies affirm the critical role of CD4^+^ CTLs in the immune response against solid tumors.^172^ Further research is needed to better understand the role of CD4^+^ CTLs in the TME and their impact on cancer immunotherapy.

CD4^+^ CTLs recognize and kill tumor cells primarily through two mechanisms. The most important mechanism involves MHC II-dependent recognition and killing of target cells. Tumor cells can evade CD8^+^ CTLs by downregulating MHC I molecules,^173^ but recent studies have shown that MHC II molecules can be ectopically expressed in the TME of certain tumors, where they retain antigen-presentation activity.^174,175^ This may serve as a compensatory mechanism for the downregulation of MHC I. In patients who have undergone radiation therapy, radiation can also upregulate the expression of MHC class II molecules and death receptors such as FAS and DR5 on the surface of tumor cells. These changes increase the immunogenicity of tumor cells, increasing their susceptibility to recognition by CD4⁺ CTLs. Upon activation, they contribute to the improved efficacy of radiotherapy by secreting Th1-type cytokines, directly lysing irradiated tumor cells, and promoting epitope spreading.^176^ The activation of CD4⁺ T cells within the TME is essential for initiating effective tumor rejection. Successful antitumor responses require the local presentation of MHC class II-restricted antigens in the TME. Notably, even in tumors that do not intrinsically express MHC class II molecules, both spontaneous and immunotherapy-induced tumor regression have been shown to depend on the coordinated activity of tumor antigen-specific CD8⁺ and CD4⁺ T cells.^177,178^

However, owing to the heterogeneity of solid tumors, MHC II-dependent recognition is not always effective. In some cases of head and neck squamous cell carcinoma and pancreatic ductal adenocarcinoma, even after IFN-γ-induced surface expression of MHC II, the TCRs of CD4^+^ T cells are unable to recognize tumor cells expressing the source protein. These findings suggest that CD4^+^ CTLs may utilize other mechanisms for recognition. In fact, CD4^+^ CTLs can also upregulate NKG2D and kill tumor cells through the NKG2D-MICA/B pathway, compensating for the elimination of tumors that do not express MHC II molecules.^5^ Furthermore, some studies have reported that CD4^+^CD8dim CTLs, which express low levels of CD8, can kill tumor cells in an MHC I-dependent manner.^110,179^ However, the evidence for this is limited, and further research is needed to confirm these findings.

Upon the initiation of recognition signals, cytokines such as IL-2 and IFN-γ can subsequently activate JAK, which in turn activates effector molecules, such as STAT1 and STAT5, thereby regulating the function, chemotaxis, and cytotoxicity of CD4^+^ CTLs.^180,181^ Among these, HDAC1 and HDAC2 serve as key negative regulators of STAT1 activation in CD4^+^ CTLs. The absence of these enzymes can promote CD4^+^ Th cell differentiation into CD4^+^ CTLs by upregulating RUNX3, which is associated with an increase in CD8 gene expression and cytotoxic transcriptional profiles.^121,182^ In murine models of colorectal cancer, CD4⁺ CTLs also exhibit elevated expression of the *Il12rb1* gene and acquire cytolytic activity through activation of the IL-12/IL-27 signaling pathway.^183^ Moreover, in non-small cell lung cancer, effective antitumor responses mediated by CD4⁺ CTLs require tumor-intrinsic expression of MHC class II and IL-15. IL-15 within the tumor microenvironment promotes the cytolytic capacity of CD4⁺ CTLs by activating the AKT–FOXO1–T-bet signaling pathway. This cytokine also induces the expression of SLC7A5, a solute carrier transporter, in a manner dependent on AKT signaling. Inhibition of SLC7A5 reverses the effects of IL-15 on CD4⁺ CTL function, suggesting a key role for metabolic support in sustaining their cytotoxic activity.^184^ Additionally, SLAMF7 has been found to be upregulated in CD4^+^ CTLs in bladder cancer and melanoma, partially regulating their cytotoxicity. This may represent a compensatory function of costimulatory signaling under tumor conditions.^80,169^

The role of CD4^+^ CTLs in tumor immunity has garnered significant attention, particularly regarding their ability to directly eliminate tumor cells. CD4^+^ CTLs primarily exert cytotoxic effects through the release of cytotoxic granules and death ligands. Within the TME, these cells can also effectively kill tumor cells through both direct contact and paracrine mechanisms, which do not rely on MHC II. One of the key mechanisms involves the indirect killing of target cells through an IFN-γ-dependent pathway. Unlike cytotoxicity confined to lytic granules within immunological synapses, IFN-γ has a broader influence, working alongside proinflammatory cytokines such as TNF-α and IL-2 to recruit and activate additional lymphocytes from the bloodstream, thus strengthening the immune response against tumors.^185^ Additionally, IFN-γ can directly exert cytotoxic or inhibitory effects on tumor cells, controlling tumor growth. It can also limit angiogenesis in the tumor bed, reducing nutrient and oxygen supplies.^186,187^ Furthermore, IFN-γ facilitates widespread and sustained cytokine signaling that alters both the local and distant TME, favoring broad tumor control.^188^ Evidence of CD4^+^ CTLs in various solid tumors has been confirmed through techniques such as single-cell RNA sequencing and flow cytometry, which show the expression of granzyme, perforin, and other lysis-related markers.^119,167–171^ In a study of bladder cancer, the TME was found to harbor diverse subsets of CD4⁺ CTLs with heterogeneous expression profiles of cytolytic genes, including GZMA, GZMB, GZMK, and PRF1. Notably, approximately half of these cells exhibit a polyfunctional phenotype and coexpress key effector cytokines such as IFN-γ and TNF-α.^169^ These observations highlight the potential immunological significance of CD4⁺ CTLs in shaping antitumor responses through both cytotoxic and inflammatory pathways. Additionally, cytotoxic lymphocytes may kill tumor cells through other mechanisms, such as necroptosis, pyroptosis, and ferroptosis, providing further explanation for non-MHC II-dependent cytotoxicity.^189^

Within the TME, interactions between CD4⁺ CTLs and other immune cell populations critically influence the strength and quality of antitumor responses. As previously discussed, CD4⁺ CTLs can secrete a broad spectrum of proinflammatory cytokines that promote the recruitment of effector cells such as macrophages and NK cells into the TME. In addition, tumor-infiltrating, cancer-specific exhausted CD8⁺ T cells are characterized by high expression of CXCL13, a chemokine that facilitates the recruitment of Tfh-like CD4⁺ CTLs and B cells. These interactions contribute to the formation of tertiary lymphoid structures within tumors, which are associated with improved antitumor immunity. Exhausted CD8⁺ T cells and Tfh-like CD4⁺ CTLs appear to cooperate synergistically to promote tumor control.^190,191^ Although CD4⁺ CTLs are generally more resistant to Treg-mediated suppression than are conventional CD4⁺ T cells, Tregs within the TME can still inhibit the acquisition of cytotoxic functions by CD4⁺ T cells through interference with IL-2–Blimp-1 signaling, thereby maintaining an immunosuppressive environment. While this regulation may prevent excessive immune activation, it can also facilitate tumor immune escape.^102^ Importantly, CD4^+^ CTLs do not always play a positive role in tumor immunity by eliminating tumors. Under certain pathological conditions, some cytotoxic regulatory T cells selectively target and kill MHC-II-presenting tumor antigen-bearing conventional DCs, thereby suppressing antigen presentation and impairing effective immune priming.^9,192^

Given the diverse roles of CD4^+^ CTLs in the development and progression of solid tumors, increasing evidence suggests their significant correlation with patient prognosis. In mismatch repair-proficient colorectal cancer, the infiltration of CD4^+^ GzmB^+^ T cells in the tumor core is positively associated with patient prognosis and serves as an independent protective factor for both overall survival and disease-free survival in treatment groups.^193^ Similarly, in melanoma, breast cancer, and head and neck cancer, the frequency of CD4^+^ CTLs, which are marked as CXCL13^+^, is correlated with overall survival, highlighting their potential prognostic value.^194,195^ In breast cancer patients, Th1 cells expressing granzyme B strongly predict chemotherapy survival rates and presurgical response and are also linked to higher rates of pathological complete response.^196^ In advanced colorectal cancer, downregulation of Th1-mediated immune responses and cytotoxicity-related genes has been associated with poor prognostic CD4 gene signatures. These findings underscore the critical role of CD4^+^ CTLs in tumor immunity and their potential as valuable biomarkers for patient prognosis.^197^

In addition, CD4^+^ CTLs, such as CTLA-4 and programmed death-ligand 1 (PD-L1) blockade, can enhance the efficacy of immune checkpoint therapy (ICT) in patients with tumors, with a significant correlation observed between their presence and improved patient survival rates.^166,198^ Intratumoral CD4⁺ CTLs exhibit high expression of either programmed cell death protein 1 (PD-1) or CD85j, with these inhibitory receptors generally expressed in a mutually exclusive manner. Simultaneous blockade of PD-1 and CD85j led to a substantial increase in both the frequency and effector function of CD4⁺ CTLs, providing strong support for dual checkpoint inhibition as a means to potentiate CD4⁺ CTL–based antitumor immunity.^184^ Moreover, CD4^+^ CTLs can complement the functionality of ICT in specific contexts. Defective antigen presentation via MHC class I molecules and disruption of IFN-γ signaling represent two major mechanisms by which tumors acquire resistance to ICT. In such settings, alternative immunotherapeutic approaches, such as adoptive transfer of autologous tumor-infiltrating CD4⁺ CTLs, are being explored as potential strategies to overcome ICT resistance. Loss of β2-microglobulin (B2M) is a hallmark of impaired MHC I expression. In mismatch repair–deficient colorectal cancers characterized by low B2M levels, increased infiltration of CD4⁺ T cells has been observed, and these tumors retain responsiveness to ICT in a CD4⁺ T-cell–dependent manner.^199^ Interestingly, in melanoma, CD4⁺ CTL–mediated cytotoxicity remains effective despite B2M deficiency or IFN-γ signaling impairment. This antitumor activity depends on the expression of CIITA, the master transcriptional regulator of the MHC class II antigen presentation pathway.^200^ Collectively, these findings suggest that leveraging tumor-specific CD4⁺ CTLs may represent a viable strategy to overcome ICT resistance. Understanding the multifaceted role of CD4^+^ CTLs in solid tumors can offer new insights and strategies for the diagnosis and treatment of these cancers.

#### Hematological cancers

Hematological cancers, also referred to as blood cancers, arise from the abnormal growth and differentiation of blood cells, leading to the disruption of normal hematopoiesis and the development of myeloid and lymphoid malignancies. These cancers can affect all systems and organs, and their incidence has been steadily increasing over recent decades.^201^ Hematological cancers primarily include leukemia, multiple myeloma, and lymphoma, which are characterized by high molecular heterogeneity, posing significant challenges for the development of universal treatment strategies and personalized therapies.^202^ These malignancies typically originate from lymphoid cells, their precursors, or myeloid precursors. Although these cells generally express MHC class I molecules, their expression is often altered or downregulated to evade immune clearance mediated by CD8^+^ CTLs.^203^ Notably, certain hematological cancer cells, such as those in diffuse large B-cell lymphoma, can constitutively express MHC class II molecules. Research has shown that the expression levels of MHC class II molecules, rather than MHC class I molecules, can effectively predict the prognosis and treatment outcomes of patients with hematological cancers.^204,205^

In fact, CD4^+^ CTL populations with antileukemia functions have been isolated and identified in patients with various hematological cancers, such as acute myeloid leukemia, chronic myeloid leukemia, acute lymphoblastic leukemia, diffuse large B-cell lymphoma, Hodgkin lymphoma, and multiple myeloma.^68,205–210^ These CD4^+^ CTLs are activated not only by direct recognition of antigens on the surface of hematologic tumor cells but also through recognition of tumor antigens processed by DCs. Studies have shown that DCs engineered to express leukemia-associated antigens can stimulate and activate CD4^+^ T cells in vitro. This method does not rely on specific predefined MHC class II restricted elements and may serve as a novel and universal therapeutic strategy.^211,212^ Moreover, MICA is specifically expressed in the CD34^+^ progenitor cells of CML patients but not in those of healthy individuals. MICA can bind specifically to NKG2D and activate immune responses, and CD4^+^ CTLs, which highly express NKG2D, may assist in the recognition of tumor cells via this pathway.^213^ Notably, enforced expression of IL-10 confers a type 1 regulatory T-cell phenotype and cytotoxic function to human CD4^+^ T cells. However, in leukemia, these cells kill bone marrow cells in an MHC class I dependent, antigen-independent manner.^214^ These findings suggest that CD4^+^ CTLs may possess more complex recognition mechanisms.

Like other diseases, CD4^+^ CTLs directly kill leukemia cells through cytotoxic granules and death receptors, and they can also trigger systemic immune responses by secreting cytokines.^215,216^ Notably, in leukemia, these cells can secrete IFN-γ, GM-CSF and M-CSF to stimulate the generation and activation of granulocytes and macrophages from hematopoietic stem cells.^142,209^ In pediatric B-cell acute lymphoblastic leukemia, CXCR6 can be used to identify CD4^+^ CTLs, which exhibit both cytotoxic activity against leukemia cells and the ability to remodel the TME to enhance antitumor immunity.^68^

Donor lymphocyte infusion and allogeneic hematopoietic stem cell transplantation are commonly used to treat hematologic malignancies. The mature T cells within the graft mediate graft-versus-leukemia (GvL) responses by directly killing leukemia cells, thereby controlling the disease. However, these T cells may also attack normal recipient cells, leading to severe acute graft-versus-host disease (GVHD). Therefore, it is crucial to develop strategies that enhance GvL responses while minimizing the incidence of acute GVHD to improve therapeutic outcomes.^217^ Current studies have shown that systemic CD28 blockade can reduce acute GVHD after allogeneic SCT while preserving protective GvL responses. The absence of CD28 is a distinctive feature of CD4^+^ CTLs, which suggests that these cells play a pivotal role in mediating the effects of GvL after transplantation.^218,219^ This unique immune phenotype allows CD4^+^ CTLs to maintain potent antitumor activity without triggering excessive GVHD, highlighting their potential in clinical therapies. These physiological functions may depend on IL-2, IL-15, IL-10, and EOMES.^220^ The use of IL-2/IL-15 agonists, such as NL-201, can stimulate conventional CD4^+^ Th cells to express granzyme B, thereby enhancing long-term control of myeloma in mouse models posttransplantation.^221^ Moreover, enhancing MHC class II antigen presentation and inhibiting the JAK pathway can further reduce the harmful effects of GVHD, indicating that these two pathways are critical in the functional development of CD4^+^ CTLs.^221,222^ High expression of granzyme B in CD4^+^ T cells is positively correlated with an increased risk of relapse after allogeneic stem cell transplantation in patients with hematologic malignancies, and it can predict both short-term and long-term clinical outcomes. This discovery provides an important biomarker for evaluating treatment efficacy and prognosis in clinical settings.^223,224^

Hematological cancers have long been at the forefront of immunotherapy applications in cancer treatment, and targeting CD4^+^ CTLs represents a novel approach for treating these malignancies.^225^ Current immunotherapy strategies, such as the combination of nilotinib and PD-L1 inhibitors, have been shown to effectively activate leukemia-specific CD4^+^ T-cell clones with a cytotoxic phenotype, promote their expansion, and reduce the expression of exhaustion markers, thereby inducing a more potent GvL response.^210,226^ These findings highlight the pivotal role of CD4^+^ CTLs in controlling hematological malignancies and underscore their therapeutic potential. Additionally, the design of T cells with specific reactivity has emerged as a promising therapeutic strategy. For example, Type 1 regulatory T cells induced by forced expression of IL-10 effectively eliminate a large proportion of primary acute myeloid leukemia cells, although their sensitivity to immune clearance varies among different leukemic cells.^208^ Furthermore, ex vivo-expanded polyviral-specific cytotoxic lymphocytes have demonstrated the ability to trigger a GvL response against refractory acute lymphoblastic leukemia cells via cross-reactivity without inducing GVHD, highlighting their unique advantage in immunotherapy.^227^ Chimeric antigen receptor (CAR) T cells have been identified as a treatment option for patients with refractory or relapsed hematological cancers. Notably, CD4^+^ CTLs play a critical role in CAR-T-cell therapy, particularly in maintaining long-term immune protection. While CD8^+^ CAR-T cells are highly effective at killing target cells, they are more susceptible to activation-induced cell death, which limits their persistence. In contrast, cytotoxic CD4^+^ CAR-T cells rapidly expand in vivo after infusion and maintain a polyclonal population, providing long-lasting protection.^39,228–230^ Future research will continue to explore therapeutic strategies targeting CD4^+^ CTLs to improve immunotherapy outcomes, particularly in the treatment of hematological cancers.

### Infectious diseases

#### Hepatitis B viruses

Despite the availability of safe and effective vaccines for more than two decades, hepatitis B virus (HBV) infection remains a significant global public health concern. Acute HBV infection is a self-limiting disease, and in most cases, individuals can effectively clear the virus within six months of initial exposure through an immune response.^231^ However, if the immune system fails to eliminate the virus completely, persistent viral replication in the liver can lead to chronic HBV infection, a major cause of viral hepatitis. Chronic viral hepatitis significantly increases the risk of liver fibrosis and cirrhosis, ultimately predisposing individuals to hepatocellular carcinoma.^232^ The immunopathogenesis of viral hepatitis is largely immune-mediated, and the outcome of HBV infection is primarily determined by the strength and efficacy of adaptive immune responses, particularly those involving effector T cells and neutralizing antibodies.^233^ Given the critical role of the immune system in viral clearance and disease progression, a comprehensive understanding of HBV-specific immune mechanisms is essential for developing more effective therapeutic and management strategies.

CD8^+^ T cells are the primary effector cells responsible for viral clearance and disease pathogenesis during acute HBV infection.^234^ However, as the disease progresses to chronic HBV infection, CD8^+^ T cells fail to mediate a prolonged and sustained immune response, resulting in T-cell dysfunction and a reduction in their numbers.^235^ In contrast, CD4^+^ CTLs, which are continuously activated by persistent antigen signals, emerge during chronic infection and play a crucial role in controlling persistent viruses, compensating for the exhaustion of CD8^+^ CTLs.^4,151^ Hepatocytes typically express low levels of MHC I molecules and do not constitutively express MHC II molecules, limiting interactions with T cells.^236^ However, in the presence of hepatitis virus and IFN-γ stimulation, hepatocytes can express MHC II molecules and acquire antigen-presenting functions, thereby activating specific CD4 T lymphocytes.^237^ The level and duration of MHC II expression may be correlated with disease outcome.^238,239^ HBV-infected hepatocytes treated with the MHC II transactivator can regulate the ERK pathway to restrict HBV transcription and partially mediate the antiviral effects of IFN-γ.^240^ In fact, single-cell RNA sequencing has revealed that the emergence of CD4^+^ CTLs in the liver tissue of chronic hepatitis B patients is associated with a functional cure, confirming the critical role of this cell subset in long-term disease control.^62^

CD4^+^ CTLs are regulated by various factors during HBV infection, which in turn affects their activation, expansion, and cytotoxicity. IL-26, which is elevated in autoimmune hepatitis, has been shown to promote T-cell activation and cytotoxic capacity.^241^ On the other hand, IL-27R signaling suppresses the cytotoxic activity of innate cytotoxic lymphocytes and is associated with advanced stages of hepatocellular carcinoma (HCC) and poor survival rates.^242^ Given that CD4^+^ CTLs share a similar transcriptome with NK cells, they may also be regulated by this pathway. B- and T-lymphocyte attenuators, which are inhibitory immune checkpoint molecules, are upregulated in HBV-associated acute-on-chronic liver failure, where they activate the PI3K‒Akt pathway to inhibit CD4^+^ T-cell activation, proliferation, and cytokine production while promoting apoptosis.^243^ Additionally, HBs can trigger the expression of CCR4 on T cells. These CCR4^+^ cells are known to produce excess immunoregulatory cytokines, such as IL-4, IL-5, IL-10, and TGF-β1, which exhibit immune-suppressive functions. Notably, CCR4 blockade enhances T-cell proliferation and increases the frequency of CD107a^+^ cells and perforin production in CD4^+^ CTLs.^244^ These results provide insight into the mechanisms by which the immune system is suppressed during HBV infection and suggest potential avenues for therapeutic intervention.

CD4^+^ CTLs in viral hepatitis exhibit a phenotype characteristic of terminally differentiated effector cells, notably CD27^-^ and CD28^-^. Furthermore, these cells highly express cytotoxic molecules such as perforin, granzyme, CD107a, IFN-γ, and TNF-α, which suggests their potential role in disease progression.^62,245^ Interestingly, studies have shown that in chronic HBV infection, CD4^+^ CTLs are predominantly from the CD69^-^ subset, with NKG2D^+^ and CD56^+^ populations showing increased levels of perforin and granzyme B, indicating that they may be activated in an innate-like manner.^246^ Like CD8^+^ CTLs, CD4^+^ CTLs may influence disease progression through both cytolytic and noncytolytic mechanisms. On the one hand, CD4^+^ CTLs can directly kill infected cells, promoting viral egress and subsequent binding with neutralizing antibodies, which may accelerate fibrosis and hepatitis.^247,248^ On the other hand, through the secretion of antiviral cytokines that disrupt the HBV lifecycle, they can suppress HBV gene expression and replication in hepatocytes, providing an alternative and potentially more effective means of viral clearance than direct cytotoxicity. In chronic HBV infection, TNF-α may exacerbate liver injury in patients with HBV recurrence, whereas IFN-γ is linked to HBV clearance. The transition of CD4 T cells that produce HBV-specific TNF-α to those that produce HBV-specific IFN-γ could be advantageous for HBV viral clearance.^27,249^

CD4^+^ CTLs are closely associated with multiple liver function indicators and disease progression in HBV infection, highlighting their potential diagnostic and therapeutic value. The number of CD4^+^ CTLs is correlated with elevated aspartate aminotransferase levels, decreased platelet counts, and fibrosis in elderly individuals, which may be a contributing factor to the more severe course of viral hepatitis in this population.^245^ Furthermore, in patients with HBV-related acute-on-chronic liver failure, NK cells exhibit impaired cytotoxic and metabolic functions, whereas CD4^+^ CTLs are highly activated and are considered a marker of patient recovery.^250^ In HBV-related hepatocellular carcinoma, the absence of perforin and granzyme A and B expression in CD4^+^ CTLs is associated with increased mortality and shorter survival times, and their cytotoxic activity is regulated by local Treg cells, suggesting that they may serve as potential prognostic markers.^251^ The development of HBV vaccines based on CD4^+^ CTLs could represent a novel therapeutic approach. In a mouse hepatocellular carcinoma model, a dendritic cell vaccine fused with HCC cells was able to activate CD4^+^ CTLs in an MHC II-dependent manner, conferring potent cytotoxicity and inhibiting tumor formation.^252,253^ These preclinical studies provide reliable evidence for further exploration of its feasibility in humans.

#### Human immunodeficiency virus (HIV)

HIV remains a major global public health issue, continuing to spread across all countries worldwide, claiming more than 40 million lives to date. Although antiretroviral therapy has drastically improved patient outcomes by maintaining viral suppression and enhancing immune function, it is important to acknowledge that a definitive cure has yet to be achieved. These findings emphasize the need for continued research into novel therapeutic approaches, particularly those that target the reservoir of latent viruses, which remains a significant barrier to curing HIV.^254^

HIV primarily targets human CD4^+^ T lymphocytes, as these cells express the main receptors CCR5 and CXCR4 for HIV entry.^11,255^ Once inside cells, HIV RNA is reverse transcribed into DNA and integrated into the host genome, establishing latent infection in resting memory CD4^+^ T cells.^256^ In addition to CD4^+^ T cells, monocytes, macrophages, and DCs also act as key HIV reservoirs, playing vital roles in innate immune responses, viral persistence, and the activation of adaptive immunity.^257–259^ These cells can process and present antigens to CD4^+^ T cells via MHC II, either through self-infection or by engulfing infected cells.^260^ Furthermore, activated CD4^+^ T cells can also express MHC II, which is commonly used as an activation marker for recently divided human effector CD4^+^ T cells.^261^ These cells can even present antigens such as HIV gp120 on their surface molecules, thereby activating other CD4^+^ T cells.^262^ Although CD4^+^ T cells are considered the main targets of HIV infection, they can also contribute to the immune response through various activation pathways, highlighting their complex role in the virus lifecycle.

Decades ago, CD4^+^ CTLs were found to exist in HIV infection and to exhibit cytotoxic activity.^11,263^ Early studies on HIV-infected patients identified a subpopulation of CD4^+^ CTLs expressing perforin. These cells begin clonal expansion early during HIV infection, exhibit a terminally differentiated phenotype (CD27^-^ and CD28^-^), and are thought to be associated with the inflammatory state of the disease.^151^ Transcriptomic analysis of CD4^+^ CTLs revealed high expression of cytotoxic genes such as granzymes A, B, K, perforin, IL-7R, CD57, and CD161, as well as the coexpression of the transcription factors Eomes and T-bet. Their cytotoxicity resembled that of CD8^+^ T cells and NK cells, but their transcriptional profile differed from that of Th1 cells, indicating that CD4^+^ CTLs may represent a distinct subset under certain pathological conditions.^264,265^ Their cytotoxic activity relies on IL-2 and MHC II molecules.^266^ IL-15 has been shown to activate the STAT5 pathway, enhancing CD4^+^ T-cell responses to HIV, as well as antigen presentation and CD8^+^ T-cell cytotoxicity. This pathway may also regulate CD4^+^ CTLs, given their similarities to CD8^+^ T cells and interactions with Th cells.^267^ Like CD8^+^ CTLs, CD4^+^ CTLs control viremia through cytolytic activity during the acute phase of HIV infection.^265^ In addition to directly eliminating infected T cells and macrophages, granzymes also play a role in targeting and cleaving HIV Gag proteins, contributing to improved immune control of the infection.^268,269^ In addition to the granzyme and death receptor pathways, nonspecific, calcium-dependent cytotoxicity mediated by CD4^+^ T cells and cells expressing envelope glycoproteins also participates in the early control of HIV infection.^270^ In addition to direct cytolysis, CD4^+^ CTLs secrete antiviral cytokines, such as IFN-γ and TNF-α, which enhance antigen presentation and strengthen HIV-specific immune responses.^271^ Moreover, CD4^+^ CTLs can secrete β-chemokines, such as MIP-1α and MIP-1β, which mediate noncytolytic inhibition of HIV replication by blocking HIV binding and entry into target cells.^272,273^ This review emphasizes the potential for clinical application while acknowledging the challenges in translating these findings into effective treatments.

The role of CD4^+^ CTLs in HIV infection, whether they lead to positive or negative outcomes, remains an area of ongoing debate. Recent studies have confirmed, through both in vitro and in vivo experiments, that CD4^+^ CTLs help clear the virus by directly killing infected cells.^4^ In the early stages of acute HIV infection, the expansion of Granzyme A^+^ HIV-specific CD4^+^ CTLs is thought to assist in controlling viral replication and is strongly associated with slower disease progression and better clinical outcomes.^265^ In individuals who have not received antiviral treatment but are able to control viral viremia, persistent expansion of HIV-1-specific CD4^+^ CTLs is observed, and these cells play a crucial role in virus clearance by secreting interferon-γ and antiviral β-chemokines. Furthermore, the breadth and intensity of the HIV-specific CD4^+^ T-cell response remain significantly stable over time, suggesting a potential role for these cells in chronic infection and long-term viral clearance.^274^

However, HIV-1-specific CD4^+^ T cells are preferentially targeted by HIV, and high viral loads significantly impair their ability to clear the virus.^275^ Transcriptomic analyses have also revealed a greater proportion of CD4^+^ CTLs in HIV-1-infected CD4^+^ T cells, indicating that these cells serve as a primary latent viral reservoir. More importantly, these cells express BCL2 and SERPINB9, which prevent apoptosis and facilitate the persistent survival of infected CD4^+^ CTLs, thereby possibly contributing to the persistence of the virus and disease progression.^276^ On the other hand, single-cell analysis revealed that during chronic HIV infection, CD4^+^ CTLs predominantly accumulate in the colon and blood rather than in the lymph nodes, which are key sites of HIV replication. They survive long-term through pathways associated with CD120b/TNFR2, leading to gut microbiota dysregulation and potentially contributing to the pathogenesis of gut-associated HIV-1.^266,277^ In lymph nodes, CD4^+^ CTLs typically lack effector functions, such as cytolytic activity and IFN-γ and β-chemokine expression, and exhibit intrinsic deficiencies in the formation of stable immunological synapses. These findings suggest that CD4^+^ CTLs in lymph nodes have limited cytotoxic potential and immune surveillance capabilities, thereby restricting their ability to fully clear HIV.^278^ Therefore, further in-depth research is needed to explore the role and underlying mechanisms of this cell population in the progression of HIV infection.

The dual role of CD4^+^ CTLs in HIV infection continues to fuel debate over whether strong activation of CD4^+^ T cells should be induced during treatment. Some early HIV vaccine candidates have shown the ability to induce CD4^+^ CTLs, which can lyse HIV-1-expressing target cells.^279,280^ However, the moderate efficacy of these vaccines indicates the need for improvements to enhance immune responses. In addition to vaccines, immunotherapy represents a promising strategy for HIV treatment. CCR5-edited CD4^+^ T cells have been shown to increase HIV-specific immunity, leading to temporary viral control in some individuals.^281^ Moreover, HIV-resistant and HIV-specific CAR-modified CD4^+^ T cells have been found to inhibit HIV replication in vitro and eliminate infected cells. Importantly, CAR-modified CD4^+^ T cells also increase the persistence and effectiveness of HIV-specific CAR-modified CD8^+^ T cells expressing the CD28ζ ICD in vivo, highlighting the therapeutic potential of engineered CD4^+^ T cells.^282^ However, further research is necessary to confirm their long-term efficacy and safety.

#### SARS-CoV-2

The coronavirus disease 2019 (COVID-19) pandemic, caused by severe acute respiratory syndrome coronavirus 2 (SARS-CoV-2), has had a devastating impact worldwide, infecting over a billion people and resulting in nearly 7 million deaths globally.^283^ SARS-CoV-2 infection progresses through three stages: nasal, pulmonary, and systemic. As the disease progresses, patients may experience worsening respiratory distress, muscle pain, and fever, eventually progressing to acute respiratory distress syndrome accompanied by multiorgan dysfunction due to the activation of the coagulation cascade.^284^ In the early stages of SARS-CoV-2 infection, a robust innate and adaptive immune response is triggered, limiting viral replication and aiding in the identification and elimination of infected cells. As a result, most individuals can fully recover without the need for hospitalization. However, for some individuals, severe infection with SARS-CoV-2 can lead to immune dysfunction, compromising both innate and adaptive immunity.^285^ Given the crucial role of cellular immunity in antiviral defense, abnormalities in T-cell function—such as excessive or insufficient activation, low-affinity TCRs, and delayed T-cell responses—can result in poor prognosis.^286–288^

Owing to advances in single-cell technology, a clear understanding of the immune landscape of SARS-CoV-2 was gained early in the pandemic. In the initial stages of infection, CD4^+^ CTLs can be detected in the blood of patients. These cells express high levels of markers related to activation and cytotoxicity, including CD25, CD38, CD69, CD194, CD279, CTLA-4, IFN-γ, granzyme B, and perforin, indicating the initiation of T-cell responses.^289,290^ These CD4^+^ CTLs also express transcripts for various chemokines (such as XCL1, XCL2, CCL3, CCL4, and CCL5), which may be linked to the pathogenesis of the disease and require further validation through animal models and large-scale cohort studies.^286^

The activation of CD4^+^ CTLs depends on APCs in SARS-CoV-2. After infection or vaccination, spike antigens are taken up by DCs, macrophages, and B cells, processed, and presented to CD4^+^ T cells through MHC class II molecules.^291,292^ IL-7 may regulate the proliferation and function of CD4^+^ CTLs after activation. Recombinant IL-7 treatment in severe patients can increase lymphocyte counts without triggering a cytokine storm or worsening lung function.^293^ Activated CD4^+^ CTLs destroy virus-infected cells by producing perforin and granzymes, which are crucial for controlling SARS-CoV-2 and preventing the virus from persistently infecting the nasal cavity.^294^ However, if the target of cytotoxicity is immune cells, the result may be the opposite. Studies have shown that in SARS-CoV-2-infected patients, the proportion of cytotoxic Tfh cells increases, whereas that of regulatory T cells decreases. Cytotoxic Tfh cells may kill B cells and inhibit germinal center responses, which could be linked to the B-cell response defects observed in some severe patients.^286^ Notably, recent multiomics analyses of granzyme K^+^ and granzyme B^+^ CD4^+^ CTLs in various disease contexts have identified a potential link between the secretion of granzyme K and the complement activation pathway, suggesting a novel mechanism of cytotoxicity regulation.^295^ In addition to direct cytotoxicity, the secretion of various cytokines, including IFN-γ, TNF-α, and IL-2, also plays an important role in the participation of CD4^+^ CTLs in the anti-SARS-CoV-2 immune response. These cytokines not only enhance inflammatory responses but also directly inhibit the replication of SARS-CoV-2.^296^ However, excessive inflammation can trigger a cytokine storm, leading to adverse clinical outcomes. Therefore, early and balanced T-cell responses are crucial for the effective treatment of SARS-CoV-2.

The strength of T-cell immunity largely determines the outcome of SARS-CoV-2 infection, with an appropriate T-cell response being closely associated with disease control. In individuals with a serum IgG response to SARS-CoV-2, the accumulation of CD4^+^ CTLs indicates a strong link between the IgG response and T-cell reactivity to the virus.^290^ During recovery, an increase in CD4^+^ CTLs suggests that CD4^+^ T-cell-mediated immune protection can persist for six months or more following natural infection.^297^ Furthermore, an increase in SARS-CoV-2-specific neutralizing antibody titers is linked to specific CD4^+^ CTLs, helping alleviate the disease burden.^298^ However, in many infected individuals, excessive accumulation of CD4^+^ CTLs may indicate overactivation of the immune response, which is usually associated with more severe clinical outcomes. In severe and critical COVID-19 survivors, an increase in CD4^+^ T cells after discharge represents prolonged immune activity, which is correlated with poorer lung function, particularly a decrease in total lung capacity, suggesting that immune dysregulation plays a key role in long-term lung recovery.^299^ Moreover, activated CD4^+^ CTLs are significantly increased in severe SARS-CoV-2 infections, indicating that hyperactivated CD4 + T-cell responses could lead to host tissue damage and exacerbate infection.^300^ In addition, delayed early innate immune responses, abnormal expression of IL-17a and IFN-γ, and overexpression of CD38 on CD4^+^ and CD8^+^ T cells suggest excessive T-cell activation.^301^ These factors are important prognostic markers for the severity and mortality of SARS-CoV-2 infections.

Vaccination has proven effective in preventing severe disease and death following SARS-CoV-2 infection. Existing vaccines, including viral vector vaccines, recombinant protein-adjuvanted vaccines, and mRNA vaccines, induce both cellular and humoral immune responses, including Th cell and germinal center reactions, promoting the formation of memory cells and significantly enhancing vaccine efficacy.^302^ Encouragingly, these vaccines stimulate CD4^+^ CTLs that express various proinflammatory and cytotoxic genes, which help mediate memory responses from T cells and B cells, providing protection.^303–305^ The spike protein is the key for SARS-CoV-2 binding to the host cell receptor and is the primary antigenic target recognized by the immune system. While genetic variants of the spike protein can evade neutralizing antibodies, spike-specific T cells typically maintain immunity against SARS-CoV-2 variants. Vaccines have been shown to induce CD4^+^ CTL responses targeting the S1 and S2 regions of the spike protein, with increased responses observed in recovering individuals. This partially explains hybrid immunity and protection against breakthrough infections.^306,307^ Moreover, both natural infection and mRNA vaccination induce specific memory CD4^+^ T cells, which long-term alter their transcriptional and epigenetic landscapes. Even nearly two years after memory formation, these memory CD4^+^ T cells remain enriched with transcripts related to cytotoxicity and interferon responses.^308^ Additionally, in recovery-phase patients, the accumulation of receptor-binding domain-specific memory CD4^+^ T cells is associated with high expression of T-bet, granzyme, and perforin. Targeting these CD4^+^ T-cell epitopes may serve as a new focus for vaccine development.^298^

For immunocompromised individuals, such as those with cancer, HIV, or solid organ transplants, SARS-CoV-2-specific immune responses may be impaired, leading to poor viral replication control. These high-risk groups need special consideration in clinical practice and vaccine strategy design.^309,310^ Fortunately, current studies confirm the efficacy of this vaccine in immunosuppressed populations. For multiple myeloma patients, vaccination induces a robust immune response with a significant expansion of CD4^+^ CTLs.^311^ In lymphoma patients treated with anti-CD20 therapy, a third dose of the mRNA vaccine enhances CD4^+^ Th1^+^ T-cell responses but has little effect on CD8^+^ T-cell responses.^312^ In HIV patients, vaccination does not diminish T-cell responses to HIV-infected cells, and these individuals do not show increased susceptibility to SARS-CoV-2 compared with healthy individuals.^313,314^ These findings highlight the essential role of CD4^+^ CTLs in vaccine-mediated protection and could lead to improved vaccine strategies.

In addition to vaccines, several novel immunotherapies may provide new treatment options for severe SARS-CoV-2 infection. The adoptive transfer of ex vivo-expanded SARS-CoV-2-specific cytotoxic lymphocytes may aid in the recovery of immunocompromised patients. Preclinical studies have shown that SARS-CoV-2-specific CD4^+^ T cells dominate the final product and exhibit specificity for viral hemagglutinin and cytolytic activity.^315^ Recent studies reported a cytokine capture system that stimulates peripheral blood mononuclear cells from recovering SARS-CoV-2-infected donors with viral peptides and identifies IFN-γ^+^ CD4^+^ T cells via flow cytometry. The selected SARS-CoV-2-specific CD4^+^ CTLs displayed a diverse T-cell receptor repertoire, with increased gene expression related to T-cell function, interleukins, pathogen defense, and tumor necrosis factor pathways, demonstrating their potential therapeutic value.^316^ While significant progress has been made in the development of CD4^+^ CTL-based therapies, further research is necessary to establish their clinical feasibility and to determine optimal treatment strategies.

### Autoimmune diseases

#### Systemic lupus erythematosus

SLE is a complex chronic autoimmune disease characterized by systemic and organ-specific manifestations driven by widespread immune dysregulation. It can affect multiple organs—including the skin, joints, heart, kidneys, and nervous system—resulting in highly heterogeneous clinical presentations that complicate diagnosis and treatment.^317^ Inflammation in SLE is generally systemic, and conditions once considered unrelated to immune dysfunction—such as atherosclerosis and arrhythmias—have been found to be strongly correlated with SLE due to underlying systemic immune dysregulation.^318,319^ The pathogenesis of SLE is driven by a complex interaction of genetic predispositions and environmental factors, which disrupt immune homeostasis, triggering autoimmunity against nucleic acids and related proteins and resulting in tissue-damaging inflammation. The clonal expansion of autoreactive T cells is a defining feature of SLE, as these cells are directly involved in modulating inflammatory responses and assisting B cells in the production of autoreactive antibodies.^320^ However, the exact mechanisms of disease development, particularly the role of immune cells in pathological processes, remain to be fully elucidated.

T-cell dysfunction is a hallmark of SLE and affects both CD4^+^ Th cells and CD8^+^ CTLs. CD4^+^ Th cells primarily function by secreting cytokines, which help orchestrate the immune response by modulating the activity of other immune cells. IL-2, a key growth and proliferation factor for T cells, is predominantly produced by CD4^+^ Th cells and is significantly reduced in SLE patients. IL-2 is not only a necessary growth factor for both CD4^+^ Th and CD8^+^ CTLs but also directly involved in cell death induced by T-cell activation. Deficiencies in both IL-2 production and receptor expression result in a significant imbalance in T-cell populations and function, leading to profound immune dysfunction.^318,320^ IL-10, a key anti-inflammatory cytokine produced mainly by Tregs, plays a pivotal role in dampening the immune response by inhibiting MHC class II expression on antigen-presenting cells and suppressing the activation and proliferation of other T cells. However, IL-10 also facilitates the differentiation of activated B cells into plasma cells, thereby driving antibody responses outside germinal centers and contributing to extrafollicular immune responses.^321^ Elevated levels of IL-10 are observed in the serum of SLE patients, and specific blockade of IL-10 has been shown to inhibit B-cell responses in SLE mouse models.^322,323^ The imbalance in cytokine secretion by CD4^+^ Th cells reflects the progression of SLE. With respect to CD8^+^ CTLs, while increased numbers of GZMH^+^ CD8^+^ CTLs have been observed in SLE patients, their cytotoxic potential is markedly reduced, as evidenced by lower levels of granzyme B and perforin production.^324^ This decline in cytotoxic activity hampers their ability to efficiently clear autoreactive B cells, thereby contributing to the persistence of autoimmune responses. Both perforin-mediated and Fas ligand-mediated cytotoxicity are crucial for controlling autoreactive B cells. Enhancing the function of CD8^+^ CTLs could suppress autoreactive B cells and limit the disease progression of SLE.^325,326^ The dysfunctions in both CD4^+^ Th and CD8^+^ CTLs collectively contribute to the pathogenesis and complications of SLE, underscoring the pivotal role of T cells in this disease.

Flow cytometry and single-cell multiomics analyses have revealed that CD8⁺ T cells in SLE patients tend to acquire a terminally differentiated and exhausted phenotype, indicative of impaired cytotoxic functionality.^7,327^ This dysfunction is likely driven by chronic antigen exposure and the immunosuppressive effects of Treg cells. In contrast, CD4⁺ CTLs have demonstrated resistance to Treg-mediated suppression in vitro and are capable of maintaining durable cytotoxic characteristics even under IL-10 stimulation.^51,328^ Under these conditions, CD4⁺ CTLs may compensate for dysfunctional CD8⁺ T cells in controlling autoreactive B cells, as they also possess cytolytic capacity. On the one hand, B cells, as potential APCs, express MHC class II molecules, which enable their recognition and elimination by CD4^+^ CTLs. However, several studies have noted that MHC II-mediated interactions between B cells and CD4^+^ T cells may inadvertently promote B-cell activation, proliferation, and differentiation, potentially exacerbating autoimmune responses and accelerating the progression of SLE.^329,330^ This highlights the complex role of MHC II in immune regulation, as it may both support immune defense and contribute to pathological conditions. On the other hand, CD4^+^ CTLs typically respond to the elevated levels of interferon present in SLE, which is a hallmark feature of the disease, by undergoing expansion.^7^ Interestingly, these cells are also major sources of IFN secretion. The production of IFN in CD4^+^ CTLs is likely driven by the activation of STAT4 and IL-12 signaling, which play central roles in promoting the differentiation and activation of Th1 cells.^331^ This process creates a positive feedback loop, where the secretion of IFN both enhances the cytotoxic potential of CD4^+^ CTLs and further fuels the inflammatory response in SLE.

Indeed, multiple studies have demonstrated the expansion of CD4^+^ CTLs in the serum of SLE patients, with single-cell resolution providing a clearer understanding of their characteristics. These expanded CD4^+^ CTLs exhibit classic cytotoxic markers, including elevated perforin and granzyme expression. Additionally, these cells are characterized as CD57^+^ CD26^-^ CD28^-^, indicating a highly differentiated effector population, as expected.^332^ These cytotoxic CD4^+^ T cells are actively engaged in the chronic inflammatory process observed in inactive SLE patients, reflecting the role of CD4^+^ CTLs in maintaining the inflammatory state. Like CD8^+^ CTLs, CD4^+^ CTLs play a critical role in the pathological processes of SLE. These cells exhibit a dual function that is highly dependent on the specific targets they engage. When CD4^+^ CTLs target autoreactive B cells, they help to resolve the autoimmune response by eliminating these cells, thereby mitigating disease progression.

However, more frequently, CD4^+^ CTLs may mediate cytotoxicity toward the body’s own tissues, contributing to the pathological tissue damage characteristic of SLE. This process is intricately linked to the expression of specific chemokine receptors. Compared with healthy controls and patients in remission, active SLE patients show marked upregulation of the surface expression of chemokine receptors, particularly CXCR3, CX3CR1, CCR2 and CCR5, on CD4^+^ CTLs.^333,334^ The upregulation of these receptors, which may be driven by high levels of IFN-α produced by plasmacytoid dendritic cells stimulated via TLR9, is significantly positively correlated with the systemic lupus erythematosus disease activity index.^335^ These chemokines interact with their respective ligands, thereby mediating the systemic migration and infiltration of CD4^+^ CTLs. For example, in the skin tissues of SLE patients, there is a notable increase in the expression of CCL2 and CCR2. This upregulation promotes the recruitment and infiltration of CD4^+^ CTLs, contributing to skin-related inflammation and damage, which is a common manifestation of SLE.^334,336^ In the glomeruli of SLE patients with proliferative lupus nephritis, elevated CX3CL1 expression has been observed, suggesting that CD4^+^ CTLs may migrate via the CX3CR1‒CX3CL1 axis.^337^ This upregulation reflects the aberrant immune activation characteristic of SLE and may be linked to the systemic inflammatory damage associated with the disease.

Nevertheless, recent studies have indicated that the direct cytotoxic effects of CD4^+^ CTLs in SLE may be somewhat restricted. Despite the elevated expression of perforin in CD4^+^ CTLs, no direct correlation has been identified between the levels of PRF1 transcripts or the corresponding protein and SLE disease activity.^338^ This observation suggests the involvement of alternative mechanisms or signaling pathways that may contribute to the progression of SLE beyond the classical cytotoxic pathway mediated by perforin. Research has demonstrated that CD4^+^ CTLs retain the capacity to produce IFN-γ, a key proinflammatory cytokine. This cytokine production is positively correlated with both disease activity and the proliferative capacity of these cells, suggesting that it may play a central role in driving disease progression in SLE.^339^ The pathogenic potential of CD4^+^ CTLs is driven by the interplay between the IL-15/IL-15R signaling pathway and the NKG2D/DAP10 signaling axis, which may open new avenues for therapeutic interventions.^88,340^

Given the multifaceted roles of CD4^+^ CTLs in SLE, a growing body of research has increasingly emphasized their potential as both diagnostic markers and therapeutic targets for this disease. Numerous studies have demonstrated a strong correlation between the frequency and functional phenotype of CD4^+^ CTLs and the systemic lupus erythematosus disease activity index. Elevated frequencies of CD4^+^ CTLs are often associated with an active autoimmune inflammatory response, serving as an indicator of disease activity. Additionally, CD4^+^ CTLs are valuable for predicting damage to vital organs, including the skin, kidneys, and cardiovascular system. Their chemotactic ability allows them to migrate to specific tissues, where they can contribute to the pathological damage observed in these organs.^327,334,335,340,341^ In light of these findings, numerous clinical and preclinical studies are actively exploring strategies to target CD4^+^ CTLs in the context of SLE. The cytotoxicity of CD4^+^ CTLs is regulated by the transcription factor ZEB2. Calcineurin inhibitor treatments have been shown to modify the cytotoxic phenotype and gene expression profiles associated with B-cell helper activity, providing a means to adjust their functional behavior.^342^ Furthermore, vitamin D has been shown to promote the expansion of peripherally induced Treg cells and the production of Th2 cytokines, which may influence the proliferation of CD4^+^ CTLs and help regulate immune balance.^343^ IL-15 plays a critical role in upregulating NKG2D expression and, together with the NKG2D signaling pathway, modulates the PI3K/AKT pathway, which is vital for CD4^+^ CTL survival and function. PI3K inhibitors have demonstrated substantial therapeutic potential in various lupus mouse models, effectively inhibiting the proliferation and cytotoxic functions of CD4^+^ CTLs.^88^ In addition, SLAMF7 has been demonstrated to restore the function of defective effector CD8^+^ T cells in SLE, and it can also enhance the cytotoxicity of CD4^+^ CTLs in tumor models, indicating promising therapeutic potential.^80,344^ These emerging therapeutic strategies targeting CD4^+^ CTLs provide valuable insights into potential treatments for SLE and other diseases associated with immune dysfunction.

#### Rheumatoid arthritis

RA is a chronic systemic autoimmune disease that primarily affects synovial joints, causing persistent inflammation and progressive joint damage. Typical symptoms include joint pain, swelling, and stiffness, which may lead to functional disability if not promptly treated. Early diagnosis is critical for preventing irreversible damage but remains challenging because of the insidious onset and symptom variability of this disease.^345^ Although the specific cause of RA remains unclear, the complex interplay among genetic predispositions, environmental factors, and dysregulated immune responses contributes to the immune system’s misidentification and attack of normal joint tissues. Anti-citrullinated protein antibodies (ACPAs) serve as specific biomarkers for RA and have been shown to be correlated with the severity and progression of the disease.^346^ ACPAs are produced as a result of an aberrant immune response to a variety of citrullinated proteins, such as fibrin, vimentin, type II collagen, and histones, which are found in various tissues throughout the body. The interaction between genetic predispositions and environmental triggers plays a crucial role in determining the reactivity of these autoantibodies to citrullinated antigens, which is central to the pathogenesis of RA.^347^ A number of citrullinated neoantigens are capable of activating MHC class II-dependent T cells, which in turn help B cells generate more ACPAs. These findings underscore the pivotal role of CD4^+^ T-cell subsets in driving the pathogenesis of RA.^348,349^

In RA patients, CD4^+^ T cells often display hyperproliferation and imbalanced proportions, contributing significantly to the pathological processes associated with the disease. These T cells may be preferentially activated in RA, particularly in individuals carrying certain HLA-DRB1 alleles, which contain a five-amino acid sequence linked to an increased risk of developing RA. In addition, citrullinated peptides are more likely to be presented by HLA-DRB1 risk alleles, further contributing to the immune activation observed in RA.^350^ CD4^+^ T cells in RA patients often display a defect in glycolytic flux, leading to the accumulation of NADPH (the reduced form of nicotinamide adenine dinucleotide phosphate) and the consumption of reactive oxygen species. This metabolic shift promotes the differentiation of CD4^+^ T cells into Th1 and Th17 subsets, both of which are recognized as pathogenic T-cell populations in RA joints, thereby driving a strong inflammatory phenotype.^123^ These CD4^+^ T cells play pivotal roles in sustaining chronic synovitis and facilitating the production of autoantibodies, making them key contributors to the immune dysregulation observed in RA.

Several decades ago, early studies reported the expansion of autoreactive CD4^+^ CTLs in RA patients.^30^ Initially, these cells proliferate in peripheral blood and express elevated levels of perforin and granzyme but lack key markers such as CD7, CD28, and CD40L, suggesting their specialized role in cytotoxicity rather than in B-cell help.^30,351^ Subsequent investigations revealed that these cells are more abundant in synovial fluid than in peripheral blood, indicating their likely involvement in the joint damage characteristic of RA.^352^ ACPA levels in RA patients correlate with the frequency of perforin^+^ CD4^+^ T cells in synovial joints, likely due to the membrane-lytic activity of perforin and the membrane attack complex, which induces significant citrullination.^353,354^ Additionally, Gzma has been shown to promote osteoclastogenesis, which contributes to the development of inflammatory arthritis in mouse models.^355^ These findings suggest potential mechanisms by which CD4^+^ CTLs may contribute to the pathogenesis of RA. In addition to their cytotoxic functions similar to those of CD8⁺ CTLs, their predominant contribution to rheumatoid arthritis appears to lie in their potent proinflammatory activity. Upon activation, these cells produce large amounts of GM-CSF, TNF-α, and IFN-γ, which drive local inflammation and promote autoreactive immune responses, highlighting their central role in disease amplification.^356^ However, the antigen specificity of CD4^+^ CTLs in RA remains a contentious issue. CMV infection has been implicated in driving the expansion of CD4^+^ CTLs in RA, which correlates with an increased risk of cardiovascular mortality, suggesting that CMV may serve as a critical antigen source in this disease.^12^ KARs are also critical molecules that facilitate the proliferation of CD4^+^ CTLs. The activation of KAR signaling induces the phosphorylation of several cellular targets, which amplifies the proliferative response to TCR-mediated stimulation, although it does not augment the cytotoxic capacity of CD4^+^ CTLs.^26,357^

Following signal activation, various regulatory molecules are involved in the transcriptional regulation and expression of cytotoxicity-related genes within CD4^+^ CTLs. Cytokines such as IL-2, IL-15, IL-21, and IL-23, along with costimulatory molecules such as SLAMF4 and 4-1BB—known to be essential for the development and maintenance of CD4⁺ CTLs—have been found to be elevated in the synovial fluid and peripheral blood of patients with RA, particularly during the active disease phase. This cytokine-rich microenvironment may create favorable conditions that support the differentiation and functional programming of cytotoxic CD4⁺ T cells in situ, potentially facilitating their expansion and pathogenic activity.^358–362^ Notably, a distinct subset of CX3CR1⁺ CD4⁺ CTLs selectively infiltrates inflamed synovial tissues in late-onset RA, and their frequency is positively correlated with disease onset and severity.^363^ These findings suggest that the CX3CR1–CX3CL1 chemotactic axis may play a pivotal role in mediating the recruitment and retention of CD4⁺ CTLs within the joint microenvironment. Despite accumulating evidence supporting their presence and potential activity, the precise role of CD4⁺ CTLs in RA pathogenesis remains to be fully elucidated, and further research is needed to better understand their function in RA progression.

Owing to the functional characteristics of CD4^+^ CTLs, they have been linked to the progression of RA. The detection of markers related to CD4^+^ CTLs, such as CX3CR1 and GZMB, has potential for assisting in the early diagnosis and monitoring of RA, facilitating timely therapeutic interventions.^363,364^ Moreover, these cells are particularly valuable in predicting the onset of cardiovascular complications in RA patients, which are a common cause of morbidity in this population. RA patients face an increased risk of cardiovascular diseases (CVDs), such as myocardial infarction, heart failure accompanied by left ventricular dilation, coronary artery inflammation, and stroke. The heightened risk is believed to arise from the combined effects of conventional cardiovascular risk factors and RA-specific factors, such as chronic inflammation and disease activity.^365^ Research has demonstrated that the expansion of CD4^+^ CTLs in RA patients, triggered by CMV infection and TNF-α, is linked to a greater risk of cardiovascular mortality.^12,366^ Additionally, the ongoing expansion of CD4^+^ CTLs in RA patients correlates with early atherosclerotic changes, including endothelial dysfunction and carotid artery wall thickening, which are more pronounced in these patients than in those without such expansion.^367^ Histological examination of cardiac tissues from RA patients with CVD revealed significant inflammatory infiltration in the myocardium, pericardium, and valves, with CD4^+^ CTLs being a key component, underscoring their substantial role in cardiovascular pathology.^368^

Given the compelling evidence linking CD4^+^ CTLs to both RA progression and cardiovascular complications, understanding their precise role and mechanisms of action will be crucial for developing targeted therapies to mitigate these effects. Abatacept, a CTLA4Ig fusion protein, works by binding to CD80/CD86 on the surface of APCs, thereby preventing their activation through costimulatory signals and consequently reducing the number of circulating CD4^+^ CTLs.^369^ This intervention has been linked to a decrease in disease activity in patients with RA, suggesting a potential pathway to alleviate this condition. TNF-α is a pivotal cytokine that triggers inflammatory responses and plays a central role in mediating local bone destruction in RA.^370^ TNF-α blockade therapy has been shown to partially reverse CD28 deficiency in CD4^+^ cells of RA patients, thereby restoring their normal functions. This, in turn, reduces the risk of myocardial infarction and enhances vascular compliance.^367^ A substantial number of RA patients with disabilities have regained the ability to work after starting anti-TNF-α treatment, further emphasizing the robust therapeutic benefits of this approach.^371^ Moreover, CX3CL1/fractalkine is a chemokine expressed on synovial cells and endothelial cells in the synovial tissue of RA patients. Its receptor, CX3CR1, is highly expressed on CD4^+^ CTLs and mediates their migration to synovial tissue. Clinical trials utilizing an antibody targeting human fractalkine have demonstrated some efficacy in treating RA patients over a 24-week period.^363,372^ These findings suggest that targeting CD4^+^ CTLs and their associated pathways holds promising potential for the treatment of RA and its cardiovascular complications, paving the way for future therapeutic innovations.

#### Inflammatory bowel disease

Inflammatory bowel disease (IBD) refers to a group of chronic gastrointestinal disorders that are mediated by the immune system and primarily include ulcerative colitis (UC) and Crohn’s disease (CD). A prominent feature of IBD patients is the disruption of intestinal immune homeostasis, which is closely associated with dysbiosis and the breakdown of the intestinal barrier. Innate immune cells produce inflammatory cytokines and other mediators, which activate inflammatory T cells and their related proinflammatory cytokines, leading to their accumulation in intestinal tissues and resulting in chronic and persistent intestinal tissue damage.^373,374^ Given the central role of the immune system in the pathogenesis of IBD, immune cell-targeted immunotherapies show promising preclinical potential.^375^ However, IBD patients typically require lifelong medication to control disease progression, maintain remission, and prevent complications.

Compared with those in healthy individuals, CD4^+^ CTLs accumulate and proliferate in the colon of IBD patients, indicating that these cell subsets may be closely involved in the onset and progression of IBD.^376^ However, unlike the predominantly proinflammatory role of CD4^+^ CTLs in other diseases, these cells exhibit both proinflammatory and anti-inflammatory phenotypes, reflecting their functional complexity at different stages of the disease. The activation and production of Th1 and Th17 cells are considered characteristic features of IBD. In both the human and mouse colon, a group of MHC-II-restricted T cells capable of expressing Th1- and Th17-related cytokines, cytotoxic molecules, and regulators of epithelial homeostasis has been identified.^377^ Single-cell analysis revealed that changes in chromatin accessibility near IBD-associated risk loci and pathogenic Th17 cell-related genes provide new insights into the molecular mechanisms of IBD. Moreover, these pathogenic Th17 cells coexpress transcriptional programs related to both Th1 and cytotoxicity, and their presence is correlated with the severity of IBD.^378^ Additionally, multiparameter flow cytometry and single-cell RNA sequencing analyses demonstrated that CD4^+^ CTLs are the primary source of granzyme B in the colonic tissue of UC patients, exhibiting a markedly “highly inflammatory” phenotype with significant upregulation of several proinflammatory cytokines, such as IFN-γ, TNF, IL-17A, IL-22, and IL-13.^379^ These findings collectively suggest that cytotoxicity may be one of the key factors in the pathogenesis and progression of IBD, as these cells contribute to damage to intestinal epithelial tissues.

Recent studies have identified a population of CD4^+^ CD8αα^+^ T cells that accumulate in the intestines of IBD patients. Despite expressing high levels of IFN-γ and granzyme B, these cells exhibit an immunosuppressive phenotype and are capable of preventing the exacerbation of intestinal inflammation.^380,381^ They originate from mature CD4^+^ T cells and undergo further differentiation through reprogramming by transcription factors.^124^ Compared with that in the intestines of healthy individuals, the proportion of CD4^+^ CD8αα^+^ T cells in the intestinal mucosa of IBD patients is significantly lower. Surprisingly, these cells express many regulatory markers associated with Treg cells and produce immunosuppressive molecules such as IL-10 and Lag3, suggesting a shift toward a proinflammatory immune phenotype in IBD that results in chronic intestinal damage.^37,382^ When these cells are adoptively transferred into Rag2^−^/^−^ immunodeficient mice, they differentiate and expand in the small intestine and other lymphoid tissues, where they exhibit cytolytic activity. Additionally, mice with a greater number of CD4^+^ CD8αα^+^ T cells exhibited a reduced inflammatory phenotype, further indicating that these cells help alleviate intestinal damage by controlling excessive inflammation.^383^ These findings suggest that these cells may play a suppressive role in the inflammatory response, suggesting a novel approach for IBD treatment. Owing to the functional similarities between CD4^+^ CD8αα^+^ T cells and Treg cells, many researchers believe that these cells are derived from Treg cells through further differentiation, despite their expression of various cytotoxic molecules.^25^ These cells exhibit physiological functions that are distinctly different from those of CD4^+^ CTLs, which resemble those of Th1 and Th17 cells, indicating the diversity in the origin and function of CD4^+^ CTLs.

In IBD, while TCR signaling plays a critical role in determining the fate of CD4^+^ CTLs, the source of the antigenic peptides presented remains unclear.^124^ Small intestinal epithelial cells, which normally express MHC class II molecules, may present luminal antigens, particularly those from intestinal IELs and lamina propria lymphocytes, as potential triggers for CD4^+^ T-cell activation.^384^ However, the exact antigen sources and their influence on CD4^+^ CTL differentiation require further clarification. Studies also indicate that, unlike in infectious diseases where CD4^+^ CTL development relies on prolonged antigen stimulation, TCR signaling is a crucial factor in the differentiation of CD4^+^ CTLs. Once differentiated, CD4^+^ CTLs can maintain effector functions even without continuous TCR signaling, a characteristic that may contribute to persistent tissue damage in chronic conditions such as IBD.^385^ Additionally, CD8αα in CD4^+^ CD8αα^+^ T cells can interact with thymic leukemia antigens expressed by intestinal epithelial cells, inhibiting the expansion of CD4^+^ CD8αα^+^ T cells. These findings suggest that antigen abundance and TCR affinity could regulate the formation of CD4^+^ CTLs.^386^

The intestine harbors a large population of microorganisms that interact with the host immune system, and these microorganisms are considered essential factors in the development of precursor T cells. The types and numbers of the gut microbiota can profoundly induce the differentiation and cytotoxicity of CD4^+^ T cells. For example, *segmented filamentous* bacteria promote the differentiation of CD4^+^ T cells into Th17 cells, whereas *Clostridium* facilitates the conversion of naive T cells into Treg cells. *Lactobacillus reuteri* and *Faecalibacterium prausnitzii* have been found to induce the differentiation and cytotoxicity of CD4^+^ CD8αα^+^ T cells in the intestinal epithelium. This process may be related to the production of tryptophan-derived indole compounds by bacteria, which subsequently activate the aryl hydrocarbon receptor in CD4^+^ T cells, leading to the downregulation of Thpok.^125,379,387–389^ Moreover, β-hexosaminidase, a conserved enzyme across commensals of the Bacteroidetes phylum, is a natural ligand for epithelial CD4^+^ T cells and is also considered a driving factor for the differentiation of CD4^+^ CTLs.^383^ These findings highlight the key role of specific microbiota in immune cell differentiation and suggest the potential of microbiota-based therapies in immune modulation. Additionally, commensal and food-derived yeasts have been shown to activate and reactivate CD4^+^ T cells in IBD. This process likely involves homologous antigens and cross-presentation, indicating that both the gut-associated microbiota and dietary microbes may contribute to the chronic activation of CD4^+^ CTL responses in IBD patients.^390^ Although this highlights potential avenues for microbiome-based therapies in IBD, additional research is needed to further elucidate the microbiome’s impact on intestinal immunity.

In addition, factors such as retinoic acid and cytokines can influence the differentiation and function of CD4^+^ CTLs in IBD. Retinoic acid, the main metabolite of vitamin A, not only induces CCR9 expression, promoting the migration of CD4^+^ CD8αα^+^ T cells to the intestinal epithelium but also enhances the TGFβ-mediated induction of Treg cells, counteracting the differentiation of proinflammatory Th17 cells.^389,391^ Therefore, retinoic acid is considered a key regulator of TGF-beta-dependent immune responses and plays an important role in intestinal immune regulation. This regulatory mechanism likely affects transcription factor levels in CD4^+^ T cells, thereby influencing their fate. The addition of TGF-β alone or in combination with retinoic acid can significantly downregulate ThPOK expression in CD4^+^ T cells while increasing the expression of CD8αα, CD103, and Runx3. The loss of ThPOK in activated CD4^+^ T cells suppresses their inflammatory potential and results in the upregulation of CD8^+^ CTLs and NK-related genes, subsequently triggering cytotoxicity and influencing the progression of intestinal inflammation.^97^

Furthermore, many cytokines are thought to play crucial regulatory roles in the establishment of cytotoxicity. IL-4, a hallmark cytokine of Th2 cells, plays an important role in immune regulation and inflammatory responses. After limited and nonrepetitive stimulation with IL-4, CD4^+^ T precursor cells can further differentiate into CD4^+^ CD8αα^+^ T cells. This process depends on the NF-κB–GATA-3 axis and regulates IL-10 secretion, helping maintain the balance of intestinal immunity.^392^ IL-7, along with IL-15, is an important homeostatic cytokine for T cells that is generally considered proinflammatory and has been found to induce cytolytic activity in T cells in the intestine.^393,394^ The combination of retinoic acid and IL-15 can activate DCs with proinflammatory traits through a JNK-dependent pathway, promoting the release of IL-12p70 and IL-23 and driving Th1 immune responses.^395^ However, unexpectedly, in IL-15 knockout mice, CD4^+^ CD8αα^+^ T cells are still present, suggesting that the generation of CD4^+^ CD8αα^+^ T cells is independent of IL-15. Additionally, a significant increase in the frequency of CD4^+^ CD8αα^+^ T cells was observed in IL-7 knockout mice. These findings suggest that IL-7 and IL-15 may serve as negative regulators of CD4^+^ CD8αα^+^ T-cell generation, highlighting the complexity and diversity of cytokine regulation.^396^

In other diseases, CD4^+^ CTLs primarily exert antigen-dependent cytotoxicity to eliminate pathogens or abnormal cells. However, in IBD, CD4^+^ CTLs predominantly exhibit immune quiescence. While they still secrete proinflammatory cytokines such as IFN-γ upon antigen stimulation, they also produce IL-10 to regulate immune responses, attempting to limit inflammation through autocrine mechanisms.^384,397^ IL-10 not only inhibits the aggregation of CD4^+^ CTLs and noncytolytic CD4^+^ T cells but also suppresses excessive proliferation of small intestinal epithelial cells and the expression of stress-induced molecules. More importantly, IL-10 can inhibit CD4^+^ T-cell proliferation and dendritic cell maturation, effectively suppressing T-cell responses and maintaining local immune quiescence in the intestine.^398^ This finding suggests that CD4^+^ CTLs may perform immunosuppressive functions similar to those of Treg cells, contributing to the maintenance of intestinal immune quiescence. However, under conditions of microbial translocation and specific antigen stimulation, CD4^+^ CTLs still exhibit potent cytotoxic and proinflammatory effects, damaging target tissues and recruiting other immune cell types.^54,377,379,399^ Compared with granzyme-negative Th1 cells, the adoptive transfer of granzyme-positive Th1 cells results in significant intestinal inflammation and damage, suggesting that granzyme-positive Th1 cells may be key contributors to IBD-induced colitis.^400^ Overall, these results emphasize the pathogenic potential of CD4^+^ CTLs in IBD and reveal their diverse roles in immune responses. Future research should focus on understanding their dual roles and identifying potential therapeutic targets in IBD.

Owing to the complexity of IBD pathogenesis and the dual role of CD4^+^ CTLs, inducing the right immunosuppressive activity in CD4^+^ T cells remains a key challenge in treatment. The composition, quantity, and metabolic products of the gut microbiota influence local immune responses, making microbiome-based therapies a promising strategy for treating gastrointestinal diseases.^401^ In both healthy individuals and IBD patients, the frequency of *Faecalibacterium prausnitzii*-specific CD4^+^ CTLs correlates with the abundance of *Faecalibacterium prausnitzii* in the gut microbiota, and this frequency is independently linked to disease activity. Treatment with microbiome-based therapies containing *Faecalibacterium prausnitzii* has been shown to reduce disease activity scores, improve colonic epithelial barrier function, and significantly lower proinflammatory cytokine levels. This effect appears to be mediated by a reduction in Treg cell loss in IBD patients, which helps balance proinflammatory and anti-inflammatory cells. Since CD4^+^ CTLs in IBD perform functions similar to those of Treg cells, microbiome therapies might promote their long-term survival and immune regulation.^125^ Monoclonal antibodies targeting proinflammatory cytokines can suppress abnormal immune responses and reduce inflammation, and they have been widely used in autoimmune disease treatment. IL-23-targeting monoclonal antibodies, which have high affinity and stability, have been shown in preclinical studies to inhibit cytokine release from CD4^+^ T cells and suppress systemic Th1/Th17 responses, alleviating the severity of colitis. This makes the use of IL-23-targeting monoclonal antibodies a promising approach for treating IBD.^402,403^ However, the accumulation of CD4^+^ CTLs with a cytotoxic phenotype, rather than a regulatory phenotype, may lead to resistance to cytokine-targeted therapies, such as infliximab and ustekinumab. Therefore, it is crucial to carefully consider the treatment strategy when these therapies are used.^379^ Additionally, combinations of anti-GITR antibodies and CD28 superagonists can induce large numbers of regulatory CD4^+^ CTLs, which secrete IL-10 and reduce both systemic and local inflammation, thus protecting the colon.^404^ However, these preclinical results need to be translated into clinical applications to benefit patients.

### Cardiovascular disease

#### Coronary artery disease

Coronary artery disease (CAD) is a chronic atherosclerotic condition that is inherently inflammatory and presents in various forms, including stable angina, unstable angina, myocardial infarction, and sudden cardiac death. CAD has been established as the leading cause of death worldwide, both in developed and developing nations, contributing significantly to global mortality rates.^405^ Atherosclerosis has been understood as a consequence of endothelial dysfunction driven by several risk factors, including hypertension and dyslipidemia, which facilitate the translocation of lipoproteins across the endothelial barrier into the subendothelial space.^406,407^ This conventional view has been the cornerstone of CAD research for decades, forming the basis for early interventions such as statin therapy and lifestyle modifications. However, the increasing body of evidence indicating the involvement of inflammatory and immune mechanisms suggests that the pathology of CAD is more complex than originally thought. Understanding the intricate interplay between lipid metabolism, vascular health, and immune responses is now critical for advancing therapeutic options and improving patient outcomes in CAD.

Both innate and adaptive immune responses are critically involved in the pathogenesis of atherosclerosis, bridging the gap between traditional cardiovascular risk factors and the onset of arterial dysfunction. Innate immune cells, especially macrophages, are central players in the formation of early atherosclerotic lesions, initiating the inflammatory cascade that drives plaque development. When triggered by factors such as oxidized lipids, macrophages infiltrate the subendothelial space, where they engulf lipids and transform into foam cells, a defining feature of early-stage atherosclerotic plaques.^408,409^ In contrast, adaptive immune responses, particularly those mediated by CD4^+^ T cells, play multifaceted roles in the progression of atherosclerosis, influencing both the inflammatory and reparative aspects of plaque development. Th1 cells, known for their secretion of proinflammatory cytokines, including IFN-γ, IL-2, and TNF-α, can activate macrophages and T cells, amplifying the inflammatory milieu within plaques and contributing to lesion destabilization.^143,410^ Conversely, Th2 cells and Tregs have protective effects by modulating immune responses, helping to limit excessive inflammation and promoting tissue repair within the vascular system.^411–413^ Understanding the interplay between innate and adaptive immunity in atherosclerosis provides insights into potential therapeutic strategies aimed at modulating immune responses to attenuate plaque formation and promote vascular health.

CD4^+^ CTL expansion has been shown in patients with CAD and high-risk individuals, in whom these cells are believed to play a unique role, exhibiting behavior distinct from that of classic T cells—this behavior may help identify them as potential therapeutic targets.^414,415^ In the plaques of CAD patients, monoclonal populations of CD4^+^ CTLs, characterized by the absence of CD28 and exhibiting proinflammatory and cytolytic traits, can be identified, highlighting their potential role in plaque instability and disease progression.^416,417^ Some researchers hypothesize that the expansion of CD4^+^ CTLs in CAD patients is driven by persistent stimulation from autoantigens, potentially contributing to the chronic inflammation observed in these individuals. However, studies have indicated that CD4^+^ CTLs do not respond to well-established self-antigens, such as collagen in rheumatoid arthritis (RA) or oxidized low-density lipoproteins (LDLs) in atherosclerosis, suggesting a unique specificity in their antigen recognition ability. Instead, CD4^+^ CTLs respond to antigens such as hHSP60 and hHSP70, which are found in atherosclerotic plaques, as well as viral antigens.^148,418,419^ Recent studies have revealed that a population of CD4^+^ T cells, which specifically recognize apolipoprotein B, the core protein of LDL cholesterol, exhibit high heterogeneity, plasticity, and cytotoxicity, indicating their potential involvement in both lipid metabolism and the immune response within atherosclerotic lesions.^420^ Nevertheless, the precise antigen specificity of CD4^+^ CTLs remains unclear, warranting further investigation to understand the full scope of their roles in atherosclerosis.

Circulating CD4^+^ CTLs that are recruited to atherosclerotic lesions are thought to play a significant role in both plaque development and rupture, contributing to disease progression and potentially exacerbating the risk of cardiovascular events. CD4^+^ CTLs are preferentially recruited to the most vulnerable regions of atherosclerotic plaques, and their accumulation is positively correlated with the size of the necrotic core, which is a key feature associated with plaque instability and rupture.^421,422^ Compared with conventional CD4^+^ T cells, CD4^+^ CTLs exhibit a marked increase in the expression of chemokine receptors, highlighting their enhanced migratory capacity and ability to localize to areas of active inflammation within plaques. In patients with acute coronary syndrome (ACS), CD28^-^ T cells adhere to immobilized CX3CL1, distinguishing them from CD4^+^ T cells in ACS patients without CD28^-^ T-cell expansion, thereby suggesting a distinct role for these cells in plaque destabilization.^423^ CCR5, a receptor for MIP-1α/CCL3 and RANTES/CCL5, is highly expressed in atherosclerotic lesions, where it plays a key role in facilitating the accumulation of T cells. In vitro studies have demonstrated that IL-12 treatment enhances the chemotaxis of CD4^+^ CTLs and promotes their transendothelial migration toward CCL5, thereby facilitating the infiltration of these cells into atherosclerotic lesions and contributing to plaque progression.^42,424^ Furthermore, P-selectin glycoprotein ligand-1 (PSGL-1) also aids CD4^+^ CTL homing to culprit coronary arteries, exacerbating plaque instability in ACS.^425^ Another study revealed that reactive oxygen species-dependent calcium release plays a pivotal role in regulating β2 integrin activation in CD4^+^ CTLs within atherosclerotic plaques, a critical step for T-cell recruitment. This process is associated with the occurrence of future cardiac events, suggesting a potential pathway for targeted therapeutic intervention.^426^

CD4^+^ CTLs possess a potent arsenal of proinflammatory and cytotoxic weapons, enabling them to intensify local inflammatory responses and directly attack cells within the vascular wall, thereby leading to detrimental effects on atherosclerotic plaque stability. Indeed, studies have shown that CD4^+^ CTLs can directly exert cytotoxic effects on endothelial and smooth muscle cells.^427,428^ CD4^+^ CTLs eliminate target cells via the cytotoxic granule pathway, a process that can be modulated by cytokines such as IL-7 and IL-15, highlighting the immune system’s dynamic control over T-cell activity in vascular environments.^88,429–431^ Moreover, IFN-γ secreted by CD4^+^ CTLs plays a crucial role in recruiting and activating macrophages within atherosclerotic plaques. This activation triggers the release of metalloproteinases, enzymes that degrade extracellular matrix components, leading to endothelial cell damage.^432^ CD4^+^ CTLs also target vascular smooth muscle cells through the death receptor pathway, as these cells in ACS patients express death receptors such as Fas and DR5, increasing their vulnerability to TRAIL-induced apoptosis.^42,433^ These findings offer a molecular framework for understanding how CD4^+^ CTLs contribute to plaque destabilization, shedding light on their pivotal role in the pathogenesis of atherosclerotic cardiovascular events.

Further investigation into the potential of CD4^+^ CTL counts as a predictor for CAD is essential for refining early diagnostic strategies. Elevated levels of CD4^+^ CTLs have been linked to an increased risk of recurrent coronary events. Both population-based prospective case‒control studies and single-cell RNA sequencing analyses have demonstrated a strong association between high CD4^+^ CTL levels and poor prognosis in patients with established CAD, reinforcing the prognostic significance of these cells in cardiovascular disease.^434,435^ Optical coherence tomography (OCT) assessments further highlighted an inverse relationship between the CD4^+^CD28^-^/Treg ratio and fibrous cap thickness. The imbalance between CD4^+^CD28A^+^ and Treg cells may significantly contribute to fibrous cap thinning, thus increasing the likelihood of plaque rupture and subsequent cardiovascular events.^436^ To fully validate the predictive potential of CD4^+^ CTLs, larger prospective cohorts involving diverse populations are essential for confirming their clinical relevance in CAD prognosis.

Targeting CD4^+^ CTLs represents a promising therapeutic strategy for CAD, offering potential benefits by addressing the immune mechanisms involved in plaque instability and progression. Selective TNF-α blockade has been shown to partially reverse CD28 deficiency in CD4^+^ cells, which may reduce the expansion of CD4^+^ CTLs and consequently decrease the risk of CAD. However, comprehensive clinical trials are needed to confirm its long-term benefits and therapeutic efficacy in CAD patients.^366,437^ In addition to their well-known effects on lowering LDL cholesterol and modulating inflammation, statins have demonstrated the ability to inhibit CD4^+^ T-cell-mediated endothelial apoptosis. This occurs by reducing the expression of CD69 and TRAIL through TCR-induced ERK activation, ultimately contributing to a reduction in cardiovascular morbidity and mortality.^438^ Additionally, statins have been shown to downregulate the antiapoptotic protein BCL-2, thereby promoting apoptosis in CD4^+^ CTLs, which may further contribute to the attenuation of inflammation and the immune response in CAD.^439,440^ Moreover, ligands for OX40 and 4-1BB, which are commonly found in atherosclerotic plaques and circulating monocytes, represent viable therapeutic targets. By inhibiting these ligands, it may be possible to reduce CD4^+^ CTL secretion of proinflammatory cytokines such as IFN-γ and TNF-α, as well as perforin release, thus improving survival outcomes in ACS patients.^81^ CD4^+^ CTLs exhibit distinct characteristics, such as defects in molecules that regulate T-apoptosis, which contribute to their resistance to programmed cell death and prolong their activation within the inflammatory microenvironment of CAD. Inhibition of proteasomal activity has been found to restore the levels of the proapoptotic factor Bim, thereby resensitizing CD4^+^ CTLs to apoptosis, which may provide a novel therapeutic approach for modulating immune responses in CAD.^149^ In addition, recent studies have suggested that selective targeting of Kv1.3, a potassium channel expressed in CD4^+^ CTLs, could offer a promising therapeutic avenue for treating ACS, potentially modulating immune cell activity in this disease.^441^ Advances in understanding the functional roles of CD4^+^ CTLs in CAD could pave the way for developing targeted elimination strategies, offering potential for more precise and effective treatments for CAD patients.

#### Atrial fibrillation

Atrial fibrillation (AF) is the most prevalent arrhythmia in clinical practice and is closely linked to increased mortality, stroke risk, and peripheral embolism. Additionally, well-established traditional risk factors, such as arterial hypertension, obesity, diabetes mellitus and genetic factors, have also been confirmed to be significant predisposing factors for AF. Fibrosis is thought to contribute to AF by inducing both electrical and structural remodeling of atrial tissues, further complicating disease pathophysiology. In recent years, accumulating data have strengthened the link between immune factors and the development of AF, indicating that immune-mediated processes could be integral to its pathogenesis and progression.^442,443^

Multiple inflammatory cell types play significant roles in the promotion of fibrosis, with each contributing through distinct mechanisms that exacerbate tissue damage and scar formation. circulating monocytes migrate into the myocardium, where they differentiate into macrophages, a crucial pathogenic cell population that significantly contributes to the development of AF.^444^ These macrophages display a broad range of phenotypes and are involved in various functions, such as the secretion of profibrotic growth factors, proinflammatory cytokines, and matrix remodeling proteases, all of which contribute to the fibrosis process.^443,445^ T cells are also recruited to the site of injury after myocardial injury through cytokine signaling and subsequently differentiate into various subsets, each with distinct roles in modulating the immune response and tissue repair. For example, Th1 cells are among the first T-cell subsets to infiltrate the myocardium, where they exert antifibrotic functions by secreting mediators such as IFN-γ and protein-10 (CXCL10), which counteract the profibrotic effects of TGF-β.^446^ During the chronic phase of myocardial injury, Th2 cells gradually replace Th1 cells as the predominant CD4^+^ T-cell subset in the myocardial tissue. Th2 cells secrete profibrotic mediators, including IL-4 and IL-13, which are known to promote cardiac fibrosis by stimulating the recruitment of monocyte-derived macrophages, which in turn regulate cardiac fibroblasts.^446,447^ Nonetheless, the role of T cells in cardiac fibrosis is multifaceted and requires further investigation to clarify their complex functions and implications in fibrosis progression.

CD4^+^ CTL counts are significantly increased in AF patients, whereas the anti-inflammatory Treg subset is markedly reduced.^448^ The pathological role of CD4^+^ CTLs in AF is linked primarily to cardiac remodeling, which is driven by a variety of mechanisms that exacerbate tissue damage and promote disease progression. CD4^+^ CTLs, which are recruited by IL-2, play a pivotal role in initiating the inflammatory response during the early stages of AF. Owing to the absence of the CD28 surface protein, CD4^+^ CTLs are resistant to both apoptosis and suppression by Tregs, which allows their accumulation over time.^148,449^ These cells secrete inflammatory cytokines such as IFN-γ and TNF-α, which are elevated in AF patients. These cytokines further recruit and activate CD8^+^ T cells, macrophages, and NK cells, amplifying the inflammatory cascade.^450^ Prolonged cardiac inflammation and remodeling lead to sustained interactions between CD4^+^ CTLs and myocardial cell surface proteins, where repeated self-antigen stimulation triggers autoreactivity, causing perforin- and granzyme-mediated cytotoxicity that damages myocardial tissue. This process results in the formation of microscars within myocardial tissue, which disrupts cardiac electrical conduction and may contribute to the initiation of fibrillatory impulses and abnormal electrical activity.^432,449,451^

Multiple population-based observational studies have identified CD4^+^ CTL fractions as independent predictors of the occurrence and prognosis of AF. AF is a serious complication that leads to significant hemodynamic deterioration, particularly in individuals with chronic heart failure (CHF), where its impact is often exacerbated. A prospective study involving 112 CHF patients demonstrated that the proportion of CD4^+^ CTLs could predict outcomes in those with AF, highlighting their potential role in disease progression.^452^ HIV-infected individuals are at increased risk of developing AF, with this elevated risk potentially linked to immune system dysregulation. Existing studies have highlighted the roles of CD4^+^ CTLs in both combating HIV infection and contributing to AF pathogenesis, suggesting that CD4^+^ CTLs may serve as critical links and potential predictive factors for AF in individuals with HIV infection.^266,453^ Interestingly, recent bioinformatic analyses revealed that periodontitis shares potential genes and immune characteristics with AF, which may increase stroke risk in AF patients. CD4^+^ CTLs are capable of driving an imbalance between Th17 and Treg cells, contributing to alveolar bone resorption in periodontitis.^454,455^ These findings indicate that immune cell infiltration and inflammation are involved in the pathophysiological mechanisms of both AF and periodontitis, with CD4^+^ CTLs potentially acting as a mediator between the two conditions.

Currently, targeted therapies aimed specifically at modulating CD4^+^ CTLs in AF patients are limited in clinical practice. Interestingly, preoperative statin therapy has been associated with a reduction in CD4^+^ CTL levels and a lower incidence of postcardiac surgery AF. These findings imply that the pathogenic and cytotoxic activities of CD4^+^ CTLs may be modulated by the anti-inflammatory effects of statins administered prior to surgery, suggesting a potential therapeutic avenue.^456,457^ However, additional prospective studies are needed to fully understand the effects of commonly used anticoagulants and antiarrhythmic drugs on CD4^+^ CTLs in AF patients, particularly their long-term implications.

#### Giant cell arteritis

Giant cell arteritis (GCA), also known as Horton’s arteritis, is the most common form of vasculitis in adults and primarily affects large- and medium-sized vessels, particularly the branches of the thoracic aorta. Inflammation of the arterial wall leads to vessel damage, narrowing, aneurysm formation, or occlusion, which form the basis of the clinical manifestations of this disease.^458^ The precise etiology and pathogenesis of GCA remain elusive, with several potential factors implicated, including a history of vascular disease, smoking, low body mass index, and aging.^459^ However, a comprehensive understanding of these mechanisms is still lacking. Previous studies have highlighted the clonal expansion of T cells within vascular lesions as a central event in the pathogenesis of GCA, with T-cell dysregulation contributing significantly to the inflammatory cascade. In individuals without GCA, CD4^+^ T cells are not typically found within arterial walls. In contrast, patients with GCA exhibit a significant increase in CD4^+^ T cells within the transmural infiltrates of the affected arteries, which underscores their critical role in driving the inflammatory process and contributing to arterial wall damage.^460^ Th1 cells, identified as effector cells in GCA due to their granulomatous characteristics, are markedly overrepresented in the serum and adventitia of newly diagnosed GCA patients, where they overexpress IFN-γ, a key cytokine that contributes to the perpetuation of inflammation.^461,462^ Treg cells are essential for maintaining immune homeostasis and regulating autoimmunity, and they are traditionally considered to counterbalance inflammatory T-cell subsets in GCA. However, recent studies suggest a paradoxical role for Treg cells, as they may exacerbate vascular inflammation in GCA by promoting the production of IL-17, a proinflammatory cytokine.^463^ This finding underscores the complexity of T-cell responses in GCA, highlighting that immune regulation in this disease is far from straightforward.

Given the distinctive characteristics of CD4^+^ CTLs, it is reasonable to infer that these cells contribute significantly to the initiation of inflammation and vascular damage in GCA. In fact, CD4^+^ CTLs can be detected in GCA patients and are often identified as CD4^+^ NKG2D^+^ T cells or CD4^+^ CD28^-^ T cells, which may reflect their altered phenotype in the context of vascular inflammation. CD4^+^ NKG2D^+^ T cells have been identified as key drivers of vasculitis and exhibit NK cell-like cytotoxicity against microvascular endothelial cells in vitro, thereby contributing to vascular damage in GCA.^464^ MICA, an NKG2D ligand, has been shown to be notably enriched in the temporal arteries of patients with GCA.^79^ Immunohistochemical analyses have shown that the expression of NKG2D serves as an indicator of costimulatory activity and may represent an alternative signaling pathway involved in the pathophysiology of GCA, suggesting potential targets for therapeutic intervention.^79^ In addition, the frequency of CD4^+^ CD28^-^ T cells is strongly correlated with the extent of disease progression, serving as an indicator of the cumulative inflammatory burden and duration of the disease.^465^ Stimulation of CD28 delivers a metabolic signal that is crucial for the pathogenic effector functions of T cells in medium- and large-vessel vasculitis, underscoring its role in sustaining chronic inflammation in these conditions.^466^ However, CD4^+^ CTLs consistently lack CD28 expression, distinguishing them from other T-cell subsets and influencing their activation and function. Currently, single-cell transcriptomic analyses have clearly demonstrated an expansion of CD4^+^ CTLs in active GCA patients, with these cells exhibiting elevated expression of cytotoxic and chemotactic genes compared with both remission patients and healthy controls.^467^ These findings highlight the pivotal role of CD4^+^ CTLs in the pathogenesis of active GCA, highlighting their potential as key mediators of vascular inflammation.

Age is considered an important risk factor for GCA, which primarily affects women over 50 years of age, and the GCA incidence increases with age. The aging process influences two key biological systems that contribute to the development of GCA: the immune system and the vascular wall niche.^468^ Studies have shown that the number of CD4^+^ CTLs increases with age and is likely to support immune responses against infections and various diseases, highlighting their role in immune surveillance during aging.^50^ Therefore, CD4^+^ CTLs may provide a mechanistic explanation for why aging is a significant risk factor for the development of GCA.

The direct cytotoxic effects of macrophages and CD8^+^ T cells are widely recognized as significant contributors to vascular injury. Interestingly, CD4^+^ CTLs exhibit similar cytotoxic mechanisms to those of CD8^+^ T cells and macrophages, highlighting their potential involvement in GCA. In active GCA patients, CD4^+^ CTLs exhibit a distinct gene expression profile, with differentially expressed genes (DEGs) being significantly enriched in pathways associated with granzyme-mediated apoptosis, inflammatory responses, and immune cell recruitment. These findings suggest that CD4^+^ CTLs play pivotal roles in driving both vascular inflammation and remodeling.^467^ Similarly, in Wegener’s granulomatosis, a distinct form of arteritis, CD4^+^ CTLs are identified as a major source of proinflammatory cytokines, including IFN-γ and TNF-α. These cytokines have been implicated in driving vascular inflammation and tissue damage.^144^ It is likely that similar production of IFN-γ and TNF-α occurs in GCA, contributing to the initiation of acute inflammation and subsequent tissue damage. Moreover, IFN-γ may play an important role in the recruitment of peripheral blood mononuclear cells in GCA by inducing the production of specific chemokines and adhesion molecules, such as CXCL9, CXCL10, and ICAM-1, by cultured vascular smooth muscle cells.^469^ Additionally, the chemokine receptor CX3CR1 accumulates in the adventitia of temporal arteries, suggesting its involvement in leukocyte extravasation. This accumulation could serve as a key step in facilitating the migration of immune cells to sites of vascular inflammation, further contributing to the progression of GCA.^79^ These chemokines are expressed on CD4^+^ CTLs, facilitating their recruitment from the circulation into inflamed tissues.

Although the critical role of CD4^+^ CTLs in GCA is increasingly recognized, clinical data supporting their direct involvement in the disease remain limited. This gap in knowledge underscores the need for more targeted research to elucidate their precise contribution to GCA pathogenesis. Nevertheless, several pharmacological agents show promise in GCA treatment. For example, maraviroc, a CCR5 antagonist, has demonstrated selective efficacy in targeting CD4^+^ CTLs in patients with active GCA, but its effect is diminished in patients in remission. This selective action is likely due to the upregulation of CCL5, a CCR5 ligand, which enhances the recruitment and activation of CD4^+^ CTLs during the active phase of the disease.^467^ These findings highlight the potential of targeting chemokine pathways as an effective therapeutic strategy for GCA. Additionally, targeting the IL-21 signaling pathway has been shown to significantly reduce the secretion of proinflammatory cytokines such as IFN-γ and IL-17A while promoting the expression of FoxP3 in CD4^+^ T cells. This intervention has the potential to restore the delicate balance between Th1, Th17, and Treg cells in GCA patients, suggesting a promising strategy to modulate the immune response and mitigate disease progression.^470^ Gaining deeper insight into the immune mechanisms driving vasculitis could lay the foundation for developing more precise and potent therapeutic strategies in the future.

#### Heart transplant rejection

Recent advances in catheter-based therapies have improved acute survival in patients with ischemic heart disease and arrhythmias. However, the incidence of refractory heart failure continues to rise, posing a major challenge for long-term management. While pharmacological treatments and ventricular assist devices offer supportive care, heart transplantation remains the most effective option for end-stage heart failure, significantly extending survival and improving outcomes.^471^ Allograft rejection is a leading cause of both morbidity and mortality after transplantation.^472^ Acute rejection is characterized by the infiltration of interstitial mononuclear cells and myocyte injury, as observed in endomyocardial biopsy, with the highest incidence occurring within the first 3–6 months post-transplant, a critical period for graft survival. The inflammatory response triggered by acute rejection can result in myocyte necrosis, hemodynamic instability, increased graft loss, the development of cardiac allograft vasculopathy, and ultimately, increased mortality rates.^473–475^ Chronic rejection, driven by ongoing alloimmune responses, progressively damages the graft over time, leading to long-term complications that severely impact both graft function and patient survival.^476^ As the mechanisms of rejection become better understood and immunosuppressive agents are optimized, the incidence of allograft rejection is gradually decreasing.

CD4^+^ T cells with indirect allogeneic specificity play a major role in allograft rejection and encompass various subsets with distinct functions. Th1 and Th17 cells are considered key mediators of allograft rejection. These cells promote allograft rejection through the secretion of key proinflammatory cytokines, including IL-2, IL-12, IL-17, IFN-γ, and TNF-α, which facilitate leukocyte recruitment and activate cytotoxic T lymphocytes, exacerbating the rejection response.^477–479^ In contrast, Treg cells are essential for inducing and maintaining transplant tolerance by suppressing the activity of alloreactive Th1, Th17, and Th2 cells, as well as inhibiting alloreactive CD8^+^ T cells and B cells that produce alloantibodies, thus preventing graft rejection.^480^ Recently, a distinct subset of TCF1^hi^CD4^+^ precursor cells with stem cell-like properties was identified. These cells can self-renew and continuously replenish the pool of effector cells, sustaining the immune response and promoting transplant rejection over time.^481–483^ This discovery may open new research directions regarding the association between allograft rejection and the CD4^+^ T-cell population.

Although researchers noted over two decades ago that CD4^+^ CTLs could function as effector cells in cardiac allograft rejection, subsequent studies have not extensively explored this phenomenon.^484,485^ However, in recent years, the pivotal role of CD4^+^ CTLs across various organ transplantation contexts has been increasingly recognized, shedding light on their importance in transplant rejection. Increased levels of CD4^+^ CTLs have been correlated with both acute and chronic rejection in liver and kidney transplant recipients, highlighting their role in graft dysfunction.^486,487^ Single-cell RNA sequencing has revealed an increased frequency of CD4^+^ CTLs in patients at high risk for early acute rejection following kidney transplantation, providing potential biomarkers for early detection.^488^ In lung transplant recipients, early increases in circulating CD4^+^ CTLs have been associated with both chronic lung allograft dysfunction and acute cellular rejection, which significantly affect long-term survival. Interestingly, these CD4^+^ CTLs are selectively inhibited by the immune checkpoint molecule HLA-G, suggesting a potential avenue for therapeutic intervention.^489,490^ Furthermore, recent studies have identified a cytotoxic gene expression signature in the tissue-resident memory T cells of lung transplant recipients characterized by elevated expression of genes such as NKG7, GZMH, GZMA, GZMB, and PRF1, indicating their potential role in transplant rejection.^491^ The direct rejection of cardiac allografts by CD4^+^ CTLs is dependent on Fas expression on the graft, along with the synergistic action of perforin, granzyme B, and TNF-α, which together induce target apoptosis. Interestingly, in a mouse skin transplantation model, the administration of TNF-α monoclonal antibodies did not prevent CD4^+^ T-cell-mediated rejection in the absence of Fas and perforin signaling, indicating that other cytotoxic pathways may be involved in allograft rejection.^492,493^ These findings suggest that monitoring CD4^+^ CTLs could provide clinicians with an effective strategy for preemptively managing transplant rejection, ultimately improving patient outcomes.

Similarly, the expansion of proinflammatory and antiapoptotic CD4^+^ CTLs is closely associated with the development of CVD after heart transplantation.^494^ A variety of factors are involved in the functional acquisition and chemotactic localization of CD4^+^ CTLs, ensuring their proper physiological role in driving allograft rejection. P38, a surface molecule involved in signal transduction, plays a pivotal role in activating CD4^+^ CTLs via the p38-MAPK signaling pathway, promoting proinflammatory responses and driving allograft rejection.^495,496^ Consequently, p38 could serve as a valuable tool for assessing the role of CD4^+^ CTLs in transplant rejection. Moreover, the allogeneic activity of CD4^+^ CTLs is driven primarily by IL-15 stimulation. Costimulation with IL-15 and IL-21 enhances CD4^+^ CTL proliferation and promotes IFN-γ production, significantly amplifying their cytotoxic functions and contributing to the exacerbation of allograft rejection.^497,498^ Cell‒cell communication analysis revealed that the CXCL9/CXCL10-CXCR3 signaling axis plays a critical role in regulating the chemotaxis of CXCL10^+^Gbp2^+^ cardiac fibroblasts and facilitating immune cell recruitment. As previously summarized, CD4^+^ CTLs can migrate to peripheral tissues via CXCR3,^499,500^ making CXCR3 a potential target to prolong allograft survival. These factors include cytokines and chemokines that orchestrate the migration and activation of CD4^+^ CTLs at the site of injury or transplantation, allowing them to exert their pathogenic effects effectively.

CMV infection remains a major posttransplant complication, impacting up to 80% of heart transplant recipients and often leading to severe graft dysfunction and increased morbidity.^501^ CD4^+^ CTLs express specific tissue-homing and cytotoxic markers that target CMV-infected tissues, contributing to endothelial injury and subsequent graft dysfunction.^502^ CMV infection is also linked to elevated long-term mortality in both kidney and cardiothoracic transplant recipients, notably increasing the risk of cardiovascular-related death.^503^ Minimizing the “indirect” consequences of CMV infection, such as immune activation and endothelial dysfunction, could significantly increase long-term transplant survival and patient quality of life.

The distinctive phenotype of CD4^+^ CTLs poses considerable challenges to traditional immunosuppressive therapies, particularly in the context of transplant rejection, where these cells often bypass conventional immunosuppression mechanisms. Belatacept, which blocks the CD80/86-CD28 costimulatory pathway, effectively sustains immunosuppression in many transplant recipients, but its efficacy is compromised in certain patients because of the downregulation of CD28 on cytotoxic T cells.^494,504^ Conventional immunosuppressive agents such as tacrolimus and everolimus are ineffective at inhibiting the in vitro proliferation of CD4^+^ CTLs, whereas drugs such as mycophenolate mofetil and corticosteroids are more effective at reducing their proliferation.^494,505^ This highlights the importance of carefully considering immunosuppressant selection and implementing individualized treatment strategies while also highlighting the potential for developing novel immune-modulating agents. Statins, known for their anti-inflammatory and cardiovascular protective properties, may offer therapeutic benefits to heart transplant recipients by reducing CD4^+^ CTL proliferation, thereby potentially limiting transplant rejection and improving graft survival.^440,506^ Additionally, single-cell transcriptional profiling highlighted the role of caspase-1 in interferon-mediated inflammatory monocyte infiltration in heart transplants. Pharmacological inhibition of caspase-1 has been shown to reduce immune cell infiltration, mitigate acute graft rejection, and enhance cardiac function, suggesting a promising avenue for improving transplant outcomes.^507^ Further research is needed to validate the therapeutic benefits of targeting CD4^+^ CTLs in heart transplant recipients and to optimize strategies for clinical application.

## Therapeutic targets and clinical research progress of CD4^+^ CTLs

**Fig. 6:**
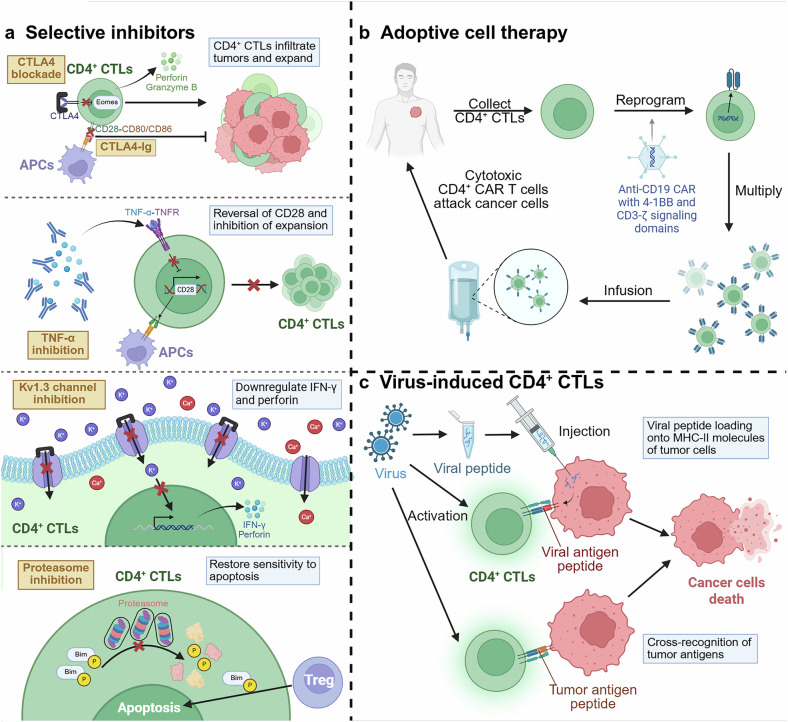
Therapeutic strategies targeting CD4^+^ CTLs. Therapeutic strategies targeting CD4^+^ CTLs can be divided into three categories: selective inhibitors (**a**), adoptive cell therapy (**b**), and virus-induced CD4^+^ CTLs (**c**). Selective inhibitors modulate the activation, proliferation, and function of CD4^+^ CTLs by targeting costimulatory molecules, cytokines, and regulatory factors associated with these cells, thereby influencing the immune balance of the body. Adoptive cell therapy, particularly CAR-T-cell therapy, involves isolating CD4^+^ CTLs from patients, editing these cells ex vivo to acquire chimeric receptors, and then reintroducing them to increase their specificity and cytotoxicity against specific antigens. Virus-induced CD4^+^ CTLs leverage the endogenous and exogenous loading of viral antigen peptides onto tumor MHC II molecules, mimicking local reinfection with a previously encountered pathogen, which triggers the reactivation of virus-specific CD4^+^ CTLs to target and eliminate tumor cells. TNFR tumor necrosis factor receptor. Created at https://BioRender.com

Although CD4^+^ cytotoxic T lymphocytes (CTLs) have been recognized for decades, the heterogeneity of this cell population has made it difficult to develop targeted therapeutic strategies, and research in this area has largely remained in the preclinical stage. However, with recent discoveries highlighting the critical role of CD4^+^ CTLs in various diseases, their therapeutic potential has gained increasing attention. As pivotal players in chronic inflammation and immune-mediated diseases, CD4^+^ CTLs hold potential as both targets and tools for therapeutic intervention. Recent clinical research has focused on several promising approaches, including the use of selective inhibitors that modulate the activity of CD4^+^ CTLs, adoptive cell therapy to increase their immunotherapeutic potential, and strategies to optimize the functional modulation of these cells. This section explores the current therapeutic strategies targeting CD4^+^ CTLs, highlighting recent progress and challenges in their clinical application (Fig. 6). Furthermore, we present a detailed summary of research efforts focused on the therapeutic implications of CD4^+^ CTLs, highlighting both clinical data and preclinical studies that shed light on their efficacy and safety^39,169,221,226,281,282,304,314–316,372,508–511^ (Table 2).

**Table 2:** Clinical trials and preclinical studies related to CD4^+^ CTLs

Studies	Conditions	Therapies	Conclusions	Study type	Trial No./Refs.
Melenhorst JJ et.	Chronic lymphocytic leukemia	Anti-CD19 CAR T cell therapy	CD4^+^ CAR T cells remained detectable more than ten years post-infusion, exhibiting cytotoxic activity, sustained functional activation, and long-term remission in both patients.	Clinical trail	NCT01029366,^39^
Patrick A Ott et.	Advanced melanoma, non-small cell lung cancer, or bladder cancer	Personalized neoantigen therapy plus Anti-PD-1	Vaccine-induced CD4^+^ and CD8^+^ T cells displayed a cytotoxic phenotype and were capable of tumor trafficking to mediate cell killing.	Clinical trail	NCT02897765,^509^
Pablo Tebas et.	HIV	CCR5-edited CD4^+^ T cells	Gene-edited CD4^+^ T cells persisted long-term and enhanced CD8^+^ CAR T cell function, with a modest delay in viral rebound relative to historical controls.	Clinical trail	NCT02388594,^281^
Luis F López-Cortés et.	HIV with SARS-COV-2 infection	BNT162b2 or mRNA-1273 vaccine	Vaccine stimulation drives CD4^+^ T cells to exhibit high functional diversity, including cytokine production, cytolytic activity, and degranulation.	Clinical trail	NCT05633927,^314^
Manuel Guerreiro et.	SARS-CoV-2	Adoptive transfer of ex vivo expanded SARS-CoV-2-specific cytotoxic lymphocytes	SARS-CoV-2-specific CD4^+^ T cells predominate in the expanded virus-specific T cell product, exhibiting cytolytic activity, with a higher proportion than CD8^+^ T cells.	Clinical trail	Universitario y Politécnico La Fe (registration number 2020-123-1),^315^
Yaya Chu et.	SARS-COV-2	Screen SARS-CoV-2-vCTLs using the CliniMACS Cytokine Capture System	SARS-CoV-2-vCTLs enriched CD4^+^ TCM and CD4^+^ TEMRA subsets, with upregulated gene expression in T cell function, interleukins, pathogen defense, and TNF superfamily pathways.	Clinical trail	NCT04896606,^316^
Zeli Zhang et.	SARS-COV-2	Moderna mRNA-1273, Pfizer/BioNTech BNT162b2, Janssen Ad26.COV2. S, and Novavax NVX-CoV2373 vaccines	At six months, all individuals developed memory CD4^+^ T cells, with cTfh and CD4^+^ CTLs prominently represented following mRNA or NVX-CoV2373 vaccination.	Clinical trail	NCT04611802,^510^
David Y Oh et.	Human bladder cancer	Anti-PD-1 immunotherapy	A gene signature of cytotoxic CD4^+^ T cells in tumors predicts a clinical response in metastatic bladder cancer patients treated with anti-PD-L1.	Clinical trail	NCT02451423,^169^
Yoshiya Tanaka et.	Rheumatoid arthritis	E6011, a humanized IgG2 monoclonal antibody against human fractalkine	E6011, a novel FKN-CX3CR1 cell trafficking inhibitor, demonstrated modest efficacy after 24 weeks in RA patients, despite not meeting the primary endpoint.	Clinical trail	NCT02960438,^372^
Hui-Nam Pak et.	Atrial fibrillation	Radio-frequency catheter ablation	The planned assays will assess the frequency of cytokine-secreting cells in CD4^+^ or CD8^+^ T cell subsets, with a focus on proinflammatory cytokines (IFN-γ, TNF-α) and cytotoxic molecules (granzyme B, perforin).	Clinical trail	NCT02421900
Thierry GUILLAUME et.	Hematologic malignancies	CTLs Anti-DP Infusion Post-hematopoietic Stem Cell Transplantation	The infusion of a third-party suicide gene-transduced T cell clone targeting HLA-DPB1*04:01 following allogeneic transplantation may be safe and provide protection against potential relapse of hematological malignancies	Clinical trail	NCT04180059
Wbeimar Aguilar-Jimenez et.	SARS-CoV-2	CoronaVac vaccine	CD4^+^ T cells exhibited a stronger virus-specific response, marked by IFN-γ/TNF-α and cytotoxic molecule production, which remained detectable even after 6 months.	Real-world study	^304^
Melenhorst JJ et.	Acute lymphoblastic leukemia	Ex-vivo expanded virus-specific donor T-cells	Virus-specific CD4^+^ and CD8^+^ CTLs with heterologous reactivity against ALL cells eliciting a GVL response without GVHD.	Case report	^508^
Minnie S.A et.	Multiple myeloma	the IL-2/IL-15 mimetic NL-201	NL-201 expanded bone marrow-resident cytotoxic memory CD8^+^ and CD4^+^ T cells resistant to exhaustion, and also promoted granzyme B production in conventional CD4⁺ T cells.	Pre-clinical study	^221^
Sean I Tracy et.	Acute B-cell leukemia	Combining nilotinib and PD-L1 blockade	Anti–PD-L1 treatment induces clonal expansion of leukemia-specific CD4^+^ T cells with a helper/cytotoxic phenotype and reduced exhaustion markers.	Pre-clinical study	^226^
Morgane Boulch et.	B cell lymphoma	Anti-CD19 CAR T cell therapy	CD4^+^ CAR T cells are more effective at host immune activation but less efficient at direct tumor killing than CD8^+^ CAR T cells.	Pre-clinical study	^511^
Colby R Maldini et.	HIV	HIV-Resistant and HIV-Specific CAR-Modified CD4^+^ T Cells	HIV-resistant CD4^+^ CAR T cells directly suppress HIV replication and enhance virus-specific CD8⁺ T cell responses.	Pre-clinical study	^282^

### Selective inhibitors

The differentiation and functional establishment of CD4^+^ CTLs is a complex process that relies on the precise coordination of various regulatory factors. In recent years, selective inhibitors targeting CD4^+^ CTLs have emerged as crucial research focuses in the field of immunotherapy. By selectively inhibiting specific functions of these cells, it is possible to effectively modulate immune responses and achieve the desired therapeutic effects in various diseases.

In addition to TCR signaling, costimulatory signals and cytokines are key factors that initiate the activation of CD4^+^ CTLs and enable them to acquire specific physiological functions. CD4^+^ CTLs typically lack CD28 expression, which increases their reliance on alternative costimulatory pathways, such as OX40 and 4-1BB. OX40 and 4-1BB are two pivotal costimulatory receptors that are critically involved not only in the initial activation of CD4^+^ CTLs but also in sustaining their functional capacity over time. These receptors ensure the persistence of the cytotoxic activity of CD4^+^ CTLs and their ability to respond to ongoing immune challenges.^59,60^ Consequently, targeted inhibition of costimulatory signals has emerged as a pivotal therapeutic strategy to modulate CD4^+^ CTL activity, particularly in disease contexts where excessive immune responses drive pathology, such as autoimmune diseases and chronic inflammatory conditions. Studies have shown that CD4^+^ CTLs exhibit excessive activation and proliferation in ACS, and the inhibition of the costimulatory receptors OX40 and 4-1BB can effectively reduce the cytotoxic response of these cells, thereby alleviating immune-mediated cardiovascular damage. Specifically, inhibiting OX40 and 4-1BB not only reduces the secretion of proinflammatory cytokines, such as IFN-γ and TNF-α but also inhibits the release of perforin, a key effector molecule involved in the cytotoxic activity of T cells, thereby reducing their potential to induce immune-mediated tissue damage. These findings suggest that selective inhibition of OX40 and 4-1BB can alter the costimulatory signaling pathways of CD4^+^ CTLs, enabling specific targeting of this cell population. Moreover, such modulation has the potential to improve survival rates in patients with ACS, highlighting its therapeutic implications.^81^

In contrast to costimulatory molecules, immune checkpoints function primarily as inhibitory receptors that downregulate T-cell activation to maintain a delicate balance between immune activation and peripheral tolerance. Well-characterized immune checkpoint molecules include PD-1, CTLA-4, and lymphocyte activation gene-3 (LAG-3), all of which play essential roles in restraining excessive immune responses and preventing autoimmunity. Recent studies have shown that CD4⁺ CTLs can express multiple immune checkpoint–related molecules on their surface and may serve as potential biomarkers for predicting patient clinical responses to ICT.^24,169,512,513^ As previously discussed, intratumoral CD4⁺ CTLs often exhibit mutually exclusive expression of inhibitory receptors such as PD-1 and CD85j. Notably, dual blockade of these molecules has been shown to significantly enhance the effector function of CD4⁺ CTLs, underscoring their potential as therapeutic targets in immune checkpoint–resistant tumors.^184^ The effector function of CD4⁺ CTLs is dependent on MHC II, allowing them to effectively circumvent ICT resistance caused by deficiencies in MHC I–mediated antigen presentation, thereby demonstrating significant therapeutic potential.^200^ In MHC-II–expressing tumors such as classical Hodgkin lymphoma, CD4⁺ T cells—rather than CD8⁺ T cells—have been shown to infiltrate the TME extensively and exhibit a marked response to PD-1 blockade. These findings suggest that CD4⁺ CTLs may serve as key effector cells that mediate ICT responses in MHC-II–dominated immune contexts. Notably, dual blockade of PD-1 and LAG-3 has demonstrated stronger antitumor activity than PD-1 inhibition alone, suggesting a promising strategy to overcome immune resistance.^210^

CTLA-4 expression is induced following the activation of CD4^+^ T cells in the blood, whereas its expression in CD8^+^ T cells is negligible in human blood and tonsils. This selective expression makes CTLA-4 a highly specific target for therapeutic strategies aimed at modulating CD4^+^ T-cell activity, offering potential for more targeted treatments in diseases involving CD4^+^ CTLs. In murine models of viral infection, CTLA-4 blockade has been shown to promote the functional reprogramming of CD4⁺ T cells, characterized by increased expression of PD-1 and the transcription factor T-bet. Notably, these CD4⁺ T cells also exhibit significant upregulation of cytotoxic effector molecules such as GzmA and GzmB, suggesting that inhibition of CTLA-4 may potentiate the cytolytic capacity of CD4⁺ T cells by driving both transcriptional and functional changes.^24^ In patients with advanced melanoma, treatment with anti–CTLA-4 monoclonal antibodies has been shown to promote Eomes–dependent expression of cytolytic granules in tumor-specific CD4⁺ CTLs. These cells acquire direct cytotoxic capacity and are capable of lysing autologous melanoma cells in an MHC class II-restricted manner, broadening the therapeutic relevance of CD4⁺ CTLs in antitumor immunity.^8^ The adoptive transfer of tumor-specific T cells has emerged as a promising immunotherapeutic approach for treating cancer. In murine models of melanoma, blockade of CTLA-4 on transferred CD4⁺ T cells led to enhanced expansion of effector T cells, a reduction in the accumulation of tumor-reactive Tregs, and—most notably—robust antitumor activity capable of inducing spontaneous tumor regression. These findings underscore the therapeutic potential of combining cytotoxic CD4⁺ T cells with immune checkpoint blockade, particularly CTLA-4 inhibition, as a novel strategy for promoting durable and effective antitumor immune responses.^166^ On the basis of the competitive binding dynamics between CTLA-4 and CD28 for the shared ligands CD80 and CD86, abatacept—a fusion protein composed of the extracellular domain of CTLA-4 linked to the Fc region of human IgG1—was specifically engineered to inhibit the costimulatory signaling pathway required for full T-cell activation. By binding with high affinity to CD80/CD86 on APCs, abatacept effectively blocks their interaction with CD28, thereby preventing the delivery of the critical second signal necessary for T-cell activation. In patients with RA, abatacept treatment has been shown to reduce the number of CD28- T cells and other effector T-cell subsets, which correlates with significant clinical improvement, particularly in terms of reducing inflammation and preventing disease progression.^369,514^

Additionally, inhibiting cytokines has emerged as a critical strategy for modulating the function of CD4^+^ CTLs. Cytokines, which are central to immune regulation, directly influence the activation, differentiation, and cytotoxic functions of these cells. TNF-α is a pivotal cytokine that regulates immune responses and plays an essential role in both maintaining the functionality and promoting the cytotoxicity of CD4^+^ CTLs. TNF-α inhibits the activity of the CD28 gene promoter, which reduces the formation of DNA‒protein complexes at the transcriptional promoter regions of CD28. This downregulation of CD28 expression on CD4^+^ T cells facilitates the acquisition of a cytotoxic phenotype, promoting the effector function of CD4^+^ CTLs.^515^ Research on patients with atherosclerosis and unstable angina has shown that CD4^+^CD28^-^ T cells continue to expand in vivo, and a higher frequency of these cells correlates with more severe clinical preatherosclerotic lesions, such as endothelial dysfunction and carotid artery wall thickening.^367,437^ Blocking TNF-α not only decreases the proliferation of CD4^+^ CTLs but also restores CD28 expression on CD4^+^ T cells, which is associated with a reduction in the risk of atherosclerosis. This therapeutic approach holds significant promise for the development of novel cardiovascular therapies that target immune-mediated inflammation in atherosclerotic diseases.

As key mediators of immune responses, interleukins play indispensable roles in regulating immune cell activation, differentiation, functional acquisition, and apoptosis. Several cytokines—including IL-2, IL-7, and IL-15—have been shown to be critical at various stages of CD4⁺ CTL development, helping to ensure the proper coordination and efficacy of the immune response.^87,88,103^ Exogenous supplementation with these cytokines has been validated in vitro as a strategy to promote CD4⁺ CTL differentiation and cytotoxic programming. However, the in vivo safety and therapeutic effectiveness of cytokine-based interventions remain to be fully elucidated, particularly in the context of chronic inflammation or cancer.^87,516^ Conversely, monoclonal antibodies targeting proinflammatory cytokines have become a cornerstone in the treatment of autoimmune diseases. These antibodies help restore immune balance by inhibiting aberrant immune responses and reducing inflammation, thus mitigating tissue damage and improving clinical outcomes.^517^ IL-21, a critical regulator of CD4^+^ CTL cytotoxicity, has been shown to drive the expansion of IL-21-producing CD4^+^ T cells in active GCA, which is positively correlated with the expansion of Th17 and Th1 cells. Selective inhibition of IL-21 has been shown to significantly reduce the secretion of proinflammatory cytokines such as IL-17A and IFN-γ while promoting the expression of the regulatory marker FoxP3. This shift not only helps restore immune balance but also plays a crucial role in mitigating the inflammatory response.^470,498^ Furthermore, monoclonal antibodies targeting IL-23, a proinflammatory cytokine that drives CD4^+^ T-cell polarization, have been shown to have high affinity and stability. In preclinical studies, these antibodies have been shown to effectively inhibit the release of cytokines from CD4^+^ T cells and suppress systemic Th1/Th17 responses. As a result, they have demonstrated potential in alleviating the severity of inflammatory conditions such as colitis, offering a promising therapeutic avenue for managing chronic autoimmune diseases.^402,403^ However, CD4^+^ CTLs may develop resistance to certain cytokine therapies, such as infliximab and ustekinumab, which target the TNF-α and IL-12/23 pathways, respectively.^379^ This resistance highlights the need for careful consideration and personalized treatment strategies to increase the long-term efficacy of such therapies.

In addition, the Kv1.3 channel, a crucial potassium ion channel in CD4^+^ CTLs, has emerged as a potential therapeutic target for modulating CD4^+^ CTL activity. This channel plays a significant role in regulating the ion fluxes that control CD4^+^ CTL activation and function. In patients with ACS, CD4^+^ CTLs express low levels of voltage-gated Kv1.3 and intermediate-conductance Ca2^+^-activated K^+^ channels (KCa3.1) under resting conditions. However, upon activation, Kv1.3 expression increases fourfold, whereas KCa3.1 levels remain relatively stable. Specific Kv1.3 channel inhibitors have been shown to effectively suppress the production of key cytotoxic molecules such as IFN-γ and perforin in CD4^+^ CTLs, thereby reducing the cytotoxic effects of these cells on target cells.^441^ This inhibition of Kv1.3 channels offers a potential therapeutic strategy to modulate excessive immune responses and tissue damage in diseases involving CD4^+^ CTLs.

In addition to targeting surface molecules, inhibiting key intracellular proteins is a promising approach for therapeutic intervention. The proteasome, a large protein complex with multiple catalytic functions, is responsible for breaking down cellular proteins into peptides in a regulated manner. This process is essential for various physiological functions, such as controlling the cell cycle, transcription, signaling pathways, and apoptosis.^518^ In CD4^+^ CTLs, the proapoptotic factor Bim is specifically degraded by the proteasome following phosphorylation, contributing to the resistance of these cells to apoptosis induced by Treg cells via Fas-ligation or ceramide. By inhibiting the proteasomal degradation of Bim, its expression can be restored in CD4^+^ CTLs, resensitizing them to apoptosis.^149^ As a key regulator of apoptosis sensitivity in CD4^+^ CTLs, Bim represents a potential therapeutic target with significant clinical implications. While early studies on selective inhibitors have shown promising efficacy in modulating immune responses, further preclinical and clinical trials are necessary to validate the long-term effectiveness and therapeutic potential of this approach, especially in confirming its safety, optimal dosing, and broader applicability.

### Adoptive cell therapy

Adoptive cell therapy (ACT) is a form of immunotherapy that involves the transfer of immune cells into a patient to combat cancer or other diseases. This approach enhances the patient’s immune system by providing a stronger and more targeted immune response. This process typically involves isolating immune cells from the patient’s body, editing and expanding these cells ex vivo, and then reinfusing them back into the patient to boost the immune response.^519^ One of the well-known forms of ACT is tumor-infiltrating lymphocyte therapy, which involves the activation and expansion of T cells that are isolated from the patient’s own tumor. These T cells inherently recognize and respond to tumor antigens, providing a direct and targeted immune response against malignancy. Additionally, genetically engineered T cells, which are often modified to express high-affinity TCRs specific to target antigens, can be utilized to enhance immune responses for the targeted elimination of tumor cells. Moreover, other receptors on the T-cell surface can be edited to further fine-tune their immune functions and enhance their effectiveness in disease eradication. Another important method is chimeric antigen receptor T (CAR-T) therapy, which involves the use of genetically engineered T cells that express chimeric antigen receptors capable of specifically recognizing tumor antigens. These engineered T cells are designed to directly target and eliminate tumor cells, resulting in a highly specific and potent immune response.^520,521^

Targeting CD4^+^ CTLs in ACT has shown substantial therapeutic potential and dynamic efficacy and has emerged as a promising new strategy for treating a variety of immune-related diseases. Typically, CD4^+^ CTLs are present in low numbers in healthy individuals but expand significantly after prolonged antigen exposure, which usually occurs later in the disease to compensate for the depletion of other immune cells. Isolation, expansion in vitro, and reinfusion of these cells at an early stage may significantly increase the ability of the host to clear pathogens, thereby providing more effective immune protection. Studies on SARS-CoV-2 have revealed that virus-specific T cells are more abundant in individuals with symptomatic infections or positive serology than in uninfected or asymptomatic individuals. These cells, primarily CD4^+^ T cells, display cytotoxic potential. Ex vivo, these viruses can survive for extended periods and expand through cultivation, exhibiting oligoclonal expansion, with specific recognition of SARS-CoV-2 viral proteins. This finding presents a promising and feasible approach for obtaining SARS-CoV-2-specific cells from seropositive donors, which can be used for adoptive transfer to immunocompromised patients who are at increased risk of severe infections.^315^ To further validate this approach, the CliniMACS cytokine capture system was employed to isolate and expand SARS-CoV-2-specific cytotoxic T lymphocytes (SARS-CoV-2-vCTLs) effectively from convalescent COVID-19 patients. After 72 hours of storage, the enriched SARS-CoV-2-vCTLs demonstrated stable survival rates, preserved CD3^+^ T-cell counts, sustained proliferative capacity, and maintained robust cytotoxic potential, indicating that the cells were viable for extended use in therapeutic applications. The enriched SARS-CoV-2-vCTLs presented a highly diverse T-cell receptor repertoire, with a marked enhancement in both CD8^+^ and CD4^+^ memory T-cell responses, underscoring the breadth of the immune response generated against SARS-CoV-2. Moreover, these cells displayed polyfunctional capabilities, with significantly increased gene expression related to critical T-cell functions, interleukins, pathogen defense mechanisms, and tumor necrosis factor pathways, indicating their robust functional capacity for therapeutic intervention.^316,522^ In clinical trials, SARS-CoV-2-vCTLs were administered to high-risk patients with progressive SARS-CoV-2 infection. Among the four patients treated, three experienced complete resolution of symptoms, whereas one maintained disease control. Importantly, none of the patients exhibited clinically significant alloimmune responses.^523^ These results highlight both the safety and therapeutic efficacy of SARS-CoV-2-vCTL-based ACT, providing a promising foundation for further exploration and clinical application in high-risk populations.

The specificity and function of gene-edited T cells are typically enhanced through genetic engineering methods. This is often achieved by editing the TCR, allowing modified T cells to efficiently and specifically recognize target antigens and cells, thus performing targeted clearance.^524^ In addition to modifying the TCR, altering key regulatory factors that govern T-cell development, differentiation, and activation has been proven to significantly influence T-cell function. This approach helps restore immune balance. A notable example is the combination of CD4^+^ T cells with leukemia cell-derived exosomes (LEXs) that have been genetically modified with costimulatory molecules. LEXs, which contain upregulated CD80 and CD86, were used to construct a novel LEX-based vaccine. This strategy effectively inhibited tumor growth and significantly extended survival in mouse models, outperforming other conventional therapies and thus demonstrating its superior therapeutic potential.^525^ Moreover, CCR5-edited CD4^+^ T cells have demonstrated encouraging results in HIV treatment by enhancing the host immune response specifically against HIV, leading to transient control of the virus. This modification targets the CCR5 receptor, a key entry point for HIV, improving the ability of cells to resist infection and modulating the immune system’s response to the virus. Remarkably, these CCR5-edited CD4^+^ T cells displayed impressive long-term persistence, with some patients showing a durable presence of these modified cells for up to five years. Despite the impressive persistence of CCR5-edited CD4^+^ T cells, HIV relapse has been observed in some patients, likely due to antigenic variation and inadequate CD8^+^ T-cell responses.^281^ This underscores the need for further clinical studies to optimize gene-editing strategies, enhance immune responses, and fully assess the long-term feasibility and safety of this approach.

CAR-T-cell therapy has gained widespread attention in recent years, largely because of its groundbreaking success in treating hematologic cancers, particularly patients with refractory or relapsed leukemia and lymphoma. These cells are genetically engineered to express a special CAR. The receptor consists of an extracellular domain that allows antigen recognition through a single-chain variable fragment, a transmembrane domain that connects the extracellular part to the intracellular portion, and an intracellular domain that includes a CD3 complex for activating downstream signaling pathways. Additionally, it typically incorporates costimulatory domains, such as CD28 and/or 4-1BB, which further enhance T-cell activation and improve the ability of T cells to recognize and kill tumor cells. Recent laboratory studies using advanced high-throughput single-cell techniques, such as nanopore grid-time imaging microscopy, have revealed that CD4⁺ CAR-T cells can simultaneously conjugate with multiple types of tumor cells and engage in serial or multikilling, demonstrating their versatility in targeting and eliminating cancer cells. Interestingly, compared with CD8^+^ CAR-T cells, CD4^+^ CAR-T cells exhibit greater resistance to activation-induced cell death, which suggests that they could provide additional therapeutic benefits, particularly in settings where prolonged immune responses are necessary.^229^ Further validation using mouse models has shown that CD4-targeted lentiviral vectors can successfully generate CD4^+^ CAR-T cells, which display Th1/Th2 phenotypes, in vivo. These cells are particularly effective at killing tumor cells, especially when the antigen load is high, likely due to the increased susceptibility of CD8^+^ T cells to exhaustion under such conditions.^228^ In clinical trials involving patients with chronic lymphocytic leukemia treated with CD19 CAR-T cells, researchers have reported that, over time, CD4^+^ T cells become increasingly activated. These highly activated CD4^+^ T cells eventually take over the CAR-T-cell response, particularly at later stages of treatment, suggesting that CD4^+^ T cells may play a crucial role in maintaining the immune response during prolonged treatment. These CD4^+^ CAR-T cells have demonstrated sustained cytotoxic activity, continuous functional activation, and proliferation, indicating that they may provide longer-lasting immune responses and more effective disease control, especially in comparison to CD8^+^ CAR-T cells, which often exhibit a higher rate of exhaustion and a shorter duration of activity.^39^

In patients with HIV, CD4^+^ CAR-T cells have been shown to directly inhibit HIV replication in vitro and effectively clear HIV-infected cells. Furthermore, these CD4^+^ CAR-T cells can enhance the in vivo persistence and effectiveness of HIV-specific CAR-modified CD8^+^ T cells, which are known to have limited longevity, thus improving overall immune response durability.^282^ Despite their promising therapeutic potential, CD4^+^ CAR-T cells do not always outperform CD8^+^ CAR-T cells, particularly in certain types of cancer where CD8^+^ T cells may offer more immediate and potent cytotoxic effects. In multiple myeloma patients who have a short treatment response, cytotoxic CD4^+^ CAR-T cells are initially abundant after infusion. However, these cells soon express exhaustion markers and are unable to maintain sustained immune activity or long-term survival, highlighting the challenge of sustaining CD4^+^ CAR-T-cell efficacy.^230^ To address this limitation, the coexpression of cytokines in CAR-T cells has emerged as a promising strategy to improve their immunological activity and persistence. In preclinical models, Glypican-3-specific CAR-T cells coexpressing IL-15 and IL-21 have demonstrated superior expansion and persistence both in vitro and in vivo, along with potent tumor-clearing capacity.^526^ These findings have also been validated in clinical settings, where Glypican-3-specific CAR-T cells coexpressing IL-15 significantly improved antitumor response rates and in vivo expansion in patients with solid tumors, outperforming conventional CAR-T cells.^527^ Moreover, the combination of engineered long-acting IL-7 with CAR-T-cell therapy has emerged as a promising strategy to prevent T-cell exhaustion and enhance antitumor responses. Recent studies have demonstrated that this combinatorial approach significantly promotes the expansion, infiltration, and persistence of CD4⁺ CAR-T cells within the tumor microenvironment, thereby leading to the effective regression of solid tumors. Mechanistically, this effect appears to be closely associated with sustained activation of the STAT5 signaling pathway.^528^ These findings emphasize the need for additional research to better understand the role of CD4^+^ CAR-T cells in disease progression after treatment, particularly with a focus on overcoming exhaustion and improving long-term immune function.

### Virus-induced CD4^+^ CTLs

Viral infections serve as significant triggers for the activation and proliferation of CD4^+^ CTLs. Upon encountering viral antigens, CD4^+^ CTLs not only expand rapidly but also acquire a memory T-cell phenotype, allowing them to persist long-term and contribute to both immediate immune responses and long-term immune memory.^265,274^ This characteristic makes CD4^+^ CTLs important players in combating viral infections. On this basis, a novel therapeutic approach focuses on reactivating T cells specific to viral antigens in the body, enabling their long-term presence and the formation of lasting functional memory.

Vaccines are a primary example of this approach. An exemplary application of T-cell-targeted vaccines involves the use of EBNA1, a protein produced during latent Epstein–Barr virus (EBV) infection, which serves as a potent stimulator of CD4^+^ T-cell responses. To harness this potential, a recombinant MVA-EL vaccine has been developed that expresses the C-terminal domain of EBNA1, which is rich in CD4^+^ T-cell epitopes, fused with the full-length latent membrane protein LMP2. This vaccine has been shown to effectively reactivate CD4^+^ memory T-cell responses in vitro, highlighting its potential for increasing CD4^+^ T-cell-mediated immunity in EBV-related diseases.^529,530^ Furthermore, to optimize the endogenous presentation of class II-restricted CTL epitopes, a polyepitope protein can be specifically targeted to class II processing compartments through endosomal or lysosomal pathways. APCs expressing this fusion protein are highly effective at activating virus-specific CD4^+^ CTLs. This approach enhances the ability of APCs to present viral antigens and stimulates more robust and focused immune activation.^531^ For HIV patients, CD4^+^ T cells are not only the target cells for HIV infection but also one of the critical mechanisms for clearing infection. A GP120-specific exosome-targeted T-cell vaccine has been demonstrated to effectively stimulate GP120-specific CD4^+^ CTL responses both in vitro and in vivo, potentially serving as an alternative to highly active antiretroviral therapy.^532^

This principle can also be applied to the expansion of CAR-T cells in vivo, whose function is often limited by a lack of T-cell expansion and persistence. By isolating and genetically modifying varicella-zoster virus-reactive T cells into varicella-zoster virus-specific CAR-T cells, these engineered cells can undergo significant expansion when exposed to the virus or after vaccination. They exhibit a CD4^+^ CTL phenotype and are capable of targeting and eliminating tumor cells in an MHC-independent fashion, offering a promising avenue for cancer immunotherapy.^533^

The discovery of the cytotoxic effector function and long-term memory phenotype of virus-specific CD4^+^ T cells indicates their potential for broader applications. These cells could be leveraged in cancer immunotherapy by exploiting their ability to control persistent antiviral immunity, particularly if virus-specific T cells can be redirected to target tumor cells. Indeed, studies on tumor-infiltrating CD4^+^ T-cell clones in glioblastoma have shown that they not only respond to glioblastoma-related tumor antigens but can also recognize peptides from pathogenic bacteria and the gut microbiota. Upon stimulation, they subsequently respond to tumor-derived target peptides, demonstrating T-cell cross-reactivity to both pathogen and tumor antigens.^534^ Similar observations have been made in melanoma, where tumor-infiltrating lymphocytes demonstrate specificity for viral antigens, including those derived from CMV, EBV, and influenza A.^535^ These findings suggest that pathogen-driven activation of CD4^+^ CTLs could play a crucial role in enhancing tumor-specific immune responses. The ability of CD4^+^ CTLs to target both pathogen and tumor antigens simultaneously opens up exciting possibilities for therapeutic interventions.

Beyond harnessing cross-reactivity, the in situ delivery of pathogen-derived peptides to mimic localized reinfection—thereby exploiting the immune activation potential of virus-induced memory T cells, a strategy known as peptide-alarm therapy—represents another promising therapeutic approach. In preclinical glioblastoma models, the direct delivery of virus-derived peptides, such as CMV, EBV, or influenza A, without the use of adjuvants has been proven to effectively reactivate virus-specific memory T cells, resulting in enhanced antitumor responses and improved survival outcomes in glioblastoma-bearing mice.^536^ Furthermore, the injection of CMV-derived T-cell epitopes into tumors leads to both local and systemic expansion of CMV-specific T cells. Furthermore, the combination of CMV CD4^+^ T-cell epitopes with poly(I:C) enhances the preferential activation of tumor-specific CD8^+^ T cells, which not only aids in tumor elimination but also offers sustained protective immunity against tumor recurrence.^537^ These results offer novel insights into how viral immunity can be leveraged to enhance tumor immunity, providing new therapeutic strategies for the eradication of solid tumors.

On the other hand, antibody-targeted pathogen-derived peptides (ATPPs) are designed by covalently attaching tumor antigen-specific antibodies expressed on the cell surface to MHC molecules. This unique combination allows ATPPs to be internalized by tumor cells, where they present antigenic peptides directly to MHC molecules without the need for traditional processing, ensuring a more direct and efficient immune response. Systemic injection of ATPPs into tumor-bearing mice has been shown to significantly increase the recruitment of virus-specific T cells to the tumor site. When combined with immune checkpoint blockade, this approach has the potential to substantially reduce tumor growth, demonstrating a synergistic effect between targeted therapy and immune modulation.^538^ Although these studies are currently focused on CD8^+^ CTLs, CD4^+^ CTLs are also capable of infiltrating tumor cells and demonstrating immune clearance capabilities similar to those of CD8^+^ CTLs. These strategies are expected to be highly effective when applied to this subset of T cells, further expanding the therapeutic potential of immune-based cancer treatments.

## Conclusion and future directions

Despite the identification of the unique subset of CD4^+^ CTLs nearly half a century ago, our understanding of these cells lags behind that of other CD4^+^ T-cell subsets owing to historical limitations in experimental techniques and immunological knowledge. The recent advent of high-dimensional technologies—such as single-cell RNA sequencing, high-parameter flow cytometry, and mass cytometry—has facilitated significant advances in defining the phenotypic features, transcriptional profiles, and developmental trajectories of CD4⁺ CTLs in various disease contexts. These tools have revealed considerable heterogeneity within CD4⁺ CTLs and highlighted their potential roles in chronic infections, cancer, autoimmune diseases, and transplantation immunity. However, a set of definitive and specific surface or transcriptional markers that reliably distinguish CD4⁺ CTLs from other CD4⁺ T-cell subsets remains elusive. Furthermore, the plasticity and lineage stability of CD4⁺ CTLs under different inflammatory or immunosuppressive conditions are still poorly understood. Further research is essential to elucidate the identification and lineage stability of CD4^+^ CTLs and the mechanisms underlying their induction and maintenance.

CD4⁺ CTLs play pivotal roles in both physiological and pathological immune responses. Under homeostatic conditions, they contribute to immune regulation and host defense against pathogens. In pathological contexts, they are implicated in a wide range of disease processes, including chronic infections, autoimmune disorders, and malignancies. Their ability to mediate MHC class II–restricted cytotoxicity, coupled with robust cytokine secretion, positions them as key effectors in both protective and deleterious immune responses. However, dysregulation of CD4⁺ CTL activity can lead to immune-mediated tissue injury, as observed in autoimmune diseases and conditions such as ACS. These dual roles underscore the importance of elucidating the mechanisms that govern their activation, differentiation, and effector function. To fully understand the protective versus pathogenic roles of CD4⁺ CTLs in disease progression, future studies could employ in vivo functional approaches, including genetic fate mapping, adoptive transfer experiments, and disease-specific animal models. A deeper understanding of these processes is essential for leveraging CD4⁺ CTLs in therapeutic interventions while minimizing their pathological consequences.

The quantification and functional assessment of CD4⁺ CTLs isolated from peripheral blood or specific tissues have been proposed as promising tools for predicting therapeutic response and clinical prognosis. For example, CD4⁺ CTL proportions are associated with the risk of CAD, providing a basis for early intervention and postoperative management. Moreover, the enrichment of CD4⁺ CTLs has been correlated with improved clinical outcomes in cancer patients receiving ICT, suggesting their utility in predicting therapeutic responsiveness. However, owing to the substantial heterogeneity of CD4⁺ CTLs across disease settings, a universal biomarker profile may not be sufficient to reliably identify this subset under all pathological conditions. Instead, context-specific combinations of surface markers, transcription factors, and functional molecules may be required for accurate detection and characterization. In addition, longitudinal analyses of the TCR repertoire across different tissues and T-cell subsets could offer valuable insights into the clonal origin and maintenance mechanisms of CD4⁺ CTLs, aiding in the identification of shared or tissue-specific clones. Such approaches may enhance our understanding of their developmental dynamics and inform future biomarker discovery. Future studies should integrate multiomics approaches and machine learning–assisted biomarker discovery to facilitate the clinical translation of CD4⁺ CTLs, expanding their potential as predictive biomarkers and therapeutic targets.

The clinical value and translational potential of CD4^+^ CTLs are increasingly recognized in the development of novel immunotherapies. Selective inhibitors targeting CD4^+^ CTLs have garnered significant attention because of their ability to modulate immune responses. Current strategies for targeting CD4^+^ CTLs include interventions aimed at costimulatory molecules, cytokines, and ion channels. These approaches can fine-tune immune responses, reduce excessive inflammation, and significantly affect the control of chronic inflammation and tissue damage associated with immune dysregulation, such as in atherosclerosis and autoimmune diseases. Additionally, through specific stimulation, genetic modification, or gene editing, the targeting and cytotoxic capabilities of CD4^+^ CTLs can be substantially enhanced. This makes CD4^+^ CTLs a critical component of ACT, as they offer more precise and effective immune interventions than conventional therapies do. Although current research has demonstrated the efficacy of CD4^+^ CTLs in clinical settings, significant challenges remain in improving their persistence and functionality, especially in chronic diseases and tumor environments. In these contexts, CD4^+^ CTLs may undergo exhaustion, limiting their long-term effectiveness. Therefore, future research should focus on optimizing the long-term survival, activation pathways, and specificity of CD4^+^ CTLs to tumors or pathogen-derived antigens. Further exploration of the role of CD4^+^ CTLs in disease progression and their potential in combination therapies will provide valuable insights into how these cells can be more effectively utilized. Combining CD4^+^ CTL-based therapies with other modalities, such as checkpoint inhibitors or targeted therapies, may offer stronger and more sustained therapeutic effects for cancers and chronic infections.

In summary, CD4^+^ CTLs represent a unique subset of CD4^+^ T cells characterized by cytotoxic functions. Harnessing the distinctive properties of CD4^+^ CTLs, such as their MHC class II specificity and immune-regulatory potential, presents exciting therapeutic possibilities. To fully elucidate this potential, a deeper understanding of CD4^+^ CTL development and plasticity is essential. Integrating CD4^+^ CTL-targeted therapies into clinical strategies, including innovative approaches such as combining them with existing therapies and vaccine design, holds promising potential. By advancing our knowledge of the biological roles and possible pathogenic involvement of CD4^+^ CTLs in various diseases, we can strengthen our arsenals for combating these conditions.
